# Dynamical boson stars

**DOI:** 10.1007/s41114-017-0007-y

**Published:** 2017-11-13

**Authors:** Steven L. Liebling, Carlos Palenzuela

**Affiliations:** 1 Long Island University, Brookville, NY 11548 USA; 2 Universitat de les Illes Balears, 07122 Palma de Mallorca, Baleares Spain

**Keywords:** Numerical relativity, Boson stars, Solitons

## Abstract

The idea of stable, localized bundles of energy has strong appeal as a model for particles. In the 1950s, John Wheeler envisioned such bundles as smooth configurations of electromagnetic energy that he called *geons*, but none were found. Instead, particle-like solutions were found in the late 1960s with the addition of a scalar field, and these were given the name *boson stars*. Since then, boson stars find use in a wide variety of models as sources of dark matter, as black hole mimickers, in simple models of binary systems, and as a tool in finding black holes in higher dimensions with only a single Killing vector. We discuss important varieties of boson stars, their dynamic properties, and some of their uses, concentrating on recent efforts.

## Introduction

Particle-like objects have a very long and broad history in science, arising long before Newton’s corpuscles of light, and spanning the range from fundamental to astronomical. In the mid-1950s, John Wheeler sought to construct stable, particle-like solutions from only the smooth, classical fields of electromagnetism coupled to general relativity (Wheeler 1955; Power and Wheeler 1957). Such solutions would represent something of a “gravitational atom”, but the solutions Wheeler found, which he called *geons*, were unstable (but see the discussion of geons in AdS in Sect. 6.3). However, in the following decade, Kaup replaced electromagnetism with a complex scalar field (Kaup 1968), and found *Klein–Gordon geons* that, in all their guises, have become well-known as today’s *boson stars* (see Sect. II of Schunck and Mielke 2003 for a discussion of the naming history of boson stars).

As compact, stationary configurations of scalar field bound by gravity, boson stars are called upon to fill a number of different roles. Most obviously, could such solutions actually represent astrophysical objects, either observed directly or indirectly through its gravity? Instead, if constructed larger than a galaxy, could a boson star serve as the dark matter halo that explains the flat rotation curve observed for most galaxies?

The equations describing boson stars are relatively simple, and so even if they do not exist in nature, they still serve as a simple and important model for compact objects, ranging from particles to stars and galaxies. In all these cases, boson stars represent a balance between the dispersive nature of the scalar field and the attraction of gravity holding it together.

This review is organized as follows. The rest of this section describes some general features about boson stars. The system of equations describing the evolution of the scalar field and gravity (i.e., the Einstein–Klein–Gordon equations) are presented in Sect. 2. These equations are restricted to the spherical symmetric case (with a harmonic ansatz for the complex scalar field and a simple massive potential) to obtain a boson-star family of solutions. To accommodate all their possible uses, a large variety of boson-star types have come into existence, many of which are described in more detail in Sect. 3. For example, one can vary the form of the scalar field potential to achieve a larger range of masses and compactnesses than with just a mass term in the potential. Certain types of potential admit soliton-like solutions even in the absence of gravity, leading to so-called Q-stars. One can adopt Newtonian gravity instead of general relativity, or construct solutions from a real scalar field instead of a complex one. It is also possible to find solutions coupled to an electromagnetic field or a perfect fluid, leading respectively to charged boson stars and fermion–boson stars. Rotating boson stars are found to have an angular momentum which is not arbitrary, but instead quantized, and can even coexist with a Kerr black hole. Multi-state boson stars with more than one complex scalar field are also considered. Recently, stars made of a massive vector field have been constructed which more closely match the original geon proposal because such a field has the same unit spin as Maxwell.

We discuss the dynamics of boson stars in Sect. 4. Arguably, the most important property of boson-star dynamics concerns their stability. Approaches to analyzing their stability include linear perturbation analysis, catastrophe theory, and fully non-linear, numerical evolutions. The latter option allows for the study of the final state of perturbed stars. Possible endstates include dispersion to infinity of the scalar field, migration from unstable to stable configurations, and collapse to a black hole. There is also the question of formation of boson stars. Full numerical evolutions in 3D allow for the merger of binary boson stars, which display a large range of different behaviors as well producing distinct gravitational-wave signatures.

Finally, we review the impact of boson stars in astronomy in Sect. 5 (as astrophysical objects, black hole mimickers, gravitational-wave sources, and sources of dark matter) and in mathematics in Sect. 6 (appearing in critical behavior, the Hoop conjecture, other dimensions and anti-de Sitter spacetimes, and gravitational analogs). We conclude with some remarks and future directions.

### The nature of a boson star

Boson stars (BS) are constructed with a complex scalar field coupled to gravity (as described in Sect. 2). A complex scalar field $\phi (t,\mathbf {r})$ can be decomposed into two real scalar fields $\phi _{\mathrm {R}}$ and $\phi _{\mathrm {I}}$ mapping every spacetime event to the complex plane

1 $$\begin{aligned} \phi (t,\mathbf {r}) \equiv \phi _{\mathrm {R}}(t,\mathbf {r}) + i \phi _{\mathrm {I}}(t,\mathbf {r}). \end{aligned}$$

Such a field possesses energy because of its spatial gradients and time derivatives, and this energy gravitates holding the star together. Less clear is what supports the star against the force of gravity. Its constituent scalar field obeys a Klein–Gordon wave equation which tends to disperse fields. This is the same dispersion which underlies the Heisenberg uncertainty principle. Indeed, Kaup’s original work (Kaup 1968) found energy eigenstates for a semi-classical, complex scalar field, discovering that gravitational collapse was not inevitable. Ruffini and Bonazzola (1969) followed up on this work by quantizing a real scalar field representing some number of bosons and they found the same field equations.

**Fig. 1:**
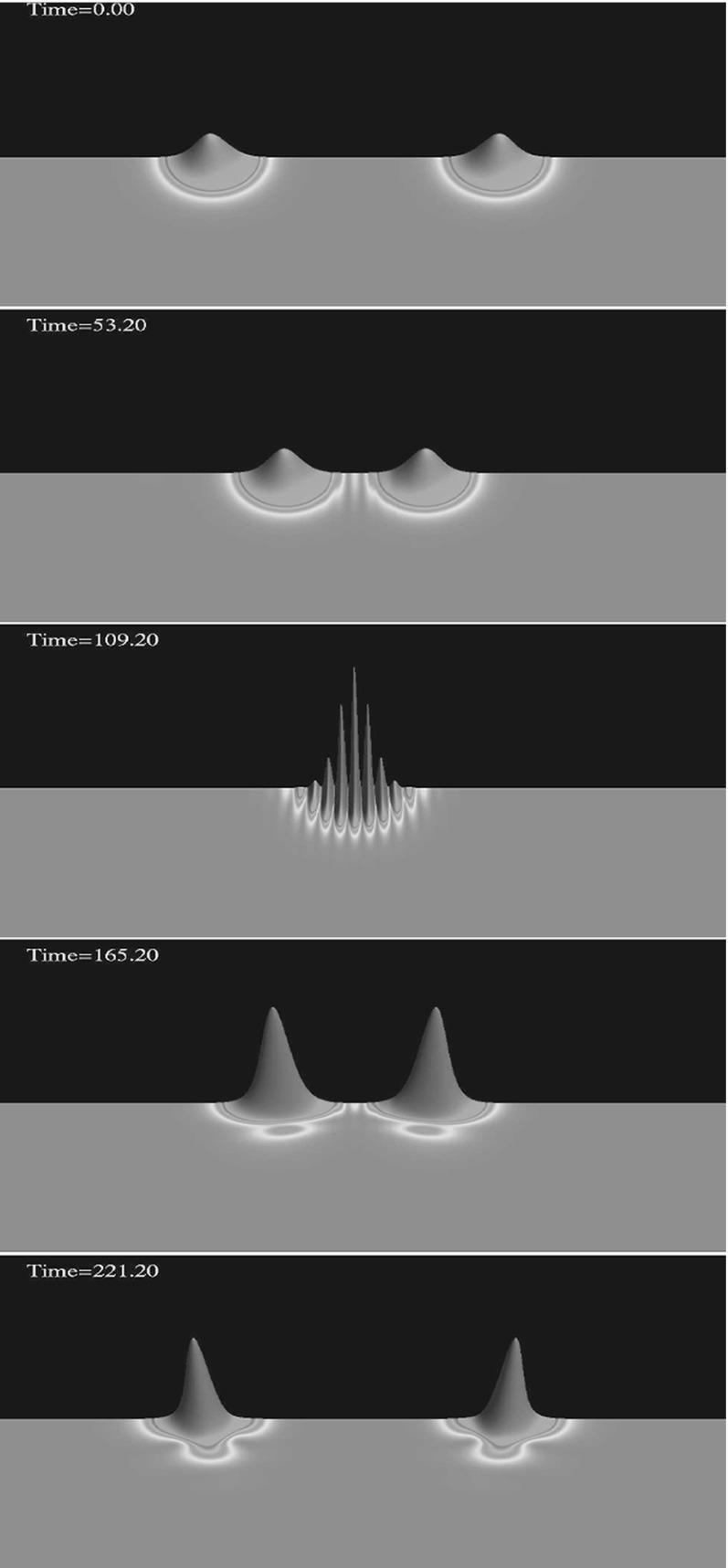
Demonstration of the solitonic nature of the (mini-)boson star. Shown are snapshots of the magnitude squared of the complex scalar field for a head-on collision of two identical mini-boson stars. The interacting stars display an interference pattern as they pass through each other, recovering their individual identities after the collision. However, note that the BSs have a larger amplitude after their interaction and so are not true solitons. The collision can therefore be considered *inelastic*. Reprinted with permission from Choi et al. (2009). See also Lai (2004) (e.g., Figure 5.12)

None of this guarantees that such solutions balancing dispersion against gravitational attraction exist. In fact, a widely known theorem, *Derrick’s theorem* (Derrick 1964) (see also Rosen 1966 and its extension to the case of a general non-canonical scalar field Diez-Tejedor and Gonzalez-Morales 2013), uses a clever scaling argument to show that no regular, static, nontopological localized scalar field solutions are stable in three (spatial) dimensional flat space. This constraint is avoided by adopting a harmonic ansatz for the complex scalar field

2 $$\begin{aligned} \phi ( \mathbf {r},t) = \phi _0(\mathbf {r}) e^{i \omega t} \end{aligned}$$

and by working with gravity. Although the field is no longer static, as shown in Sect. 2 the spacetime remains static. The star itself is a stationary, soliton-like solution as demonstrated in Fig. 1.

There are, of course, many other soliton and soliton-like solutions in three dimensions finding a variety of ways to evade Derrick’s theorem. For example, the field-theory monopole of ’t Hooft and Polyakov is a localized solution of a properly gauged triplet scalar field. Such a solution is a topological soliton because the monopole possesses false vacuum energy which is topologically trapped. The monopole is one among a number of different topological defects that requires an infinite amount of energy to “unwind” the potential energy trapped within (see Vilenkin and Shellard 1994 for a general introduction to defects and the introduction of Ryder 1996 for a discussion of relevant classical field theory concepts).

In Sect. 2, we present the underlying equations and mathematical solutions, but here we are concerned with the physical nature of these boson stars. When searching for an actual boson star, we look not for a quantized wave function or even a semiclassical one. Instead, we look to a fundamental scalar to provide the bosonic material of the star. Only recently has a scalar particle been experimentally found with the discovery by the Large Hadron Collider (LHC) of the standard model Higgs boson with a mass roughly $125\mathrm {\ GeV}/c^2$ (Aad 2012; Chatrchyan 2012; Khachatryan et al. 2015). Of course, other proposed bosonic candidates remain, such as the axion particle.

Boson stars are then either a collection of stable fundamental bosonic particles bound by gravity, or else a collection of unstable particles that, with the gravitational binding, have an inverse process efficient enough to reach an equilibrium. They can thus be considered a Bose–Einstein condensate (BEC), although boson stars can also exist in an excited state as well.

Indeed, applying the uncertainty principle to a boson star by assuming it to be a macroscopic quantum state results in an excellent estimate for the maximum mass of a BS. One begins with the Heisenberg uncertainty principle of quantum mechanics

3 $$\begin{aligned} \varDelta p\, \varDelta x \ge \hbar \end{aligned}$$

and assumes the BS is confined within some radius $\varDelta x = R$ with a maximum momentum of $\varDelta p = mc$ where *m* is the mass of the constituent particle

4 $$\begin{aligned} m c R \ge \hbar . \end{aligned}$$

This inequality is consistent with the star being described by a Compton wavelength of $\lambda _{\mathrm {C}} = h/(mc)$. We look for the maximum possible mass $M_{\max }$ for the boson star which will saturate the uncertainty bound and drive the radius of the star towards its Schwarzschild radius $R_{\mathrm {S}} \equiv 2\,GM/c^2$. Substituting yields

5 $$\begin{aligned} \frac{2\,G m\,M_{\max }}{c} = \hbar , \end{aligned}$$

which gives an expression for the maximum mass

6 $$\begin{aligned} M_{\max } = \frac{1}{2} \frac{\hbar c}{Gm}. \end{aligned}$$

Recognizing the Planck mass $M_{\mathrm {Planck}} \equiv \sqrt{\hbar c/G}$, we obtain the estimate of $M_{\max } = 0.5\,M^2_{\mathrm {Planck}}/m$. This simple estimate indicates that the maximum mass of the BS is inversely related to the mass of the constituent scalar field. We will see below in Sect. 2 that this inverse relationship continues to hold with the explicit solution of the differential equations for a simple mass term in the potential, but can vary with the addition of self-interaction terms. Indeed depending on the strength of the coupling *m* and the other parameters of the self-interaction potential, the size and mass of the boson stars can vary from atomic to astrophysical scales.

Despite their connection to fundamental physics, one can also view boson stars in analogy with models of neutron stars. In particular, as we discuss in the following sections, both types of stars demonstrate somewhat similar mass versus radius curves for their solutions with a transition in stability at local maxima of the mass. There is also a correspondence between (massless) scalar fields and a stiff, perfect fluid (see Sect. 2.1 and Appendix A of Brady et al. 2002), but the correspondence does not mean that the two are equivalent (Faraoni 2012). More than just an analogy, boson stars can serve as a very useful model of a compact star, having certain advantages over a fluid neutron star model: (i) the equations governing its dynamics avoid developing discontinuities, in particular there is no sharp stellar surface, (ii) there is no concern about resolving turbulence, and (iii) one avoids uncertainties in the equation of state.

### Other reviews

A number of other reviews of boson stars have appeared. Most recently, Schunck and Mielke (2003) concentrate on the possibility of detecting BS, extending their previous reviews (Mielke and Schunck 1999, 2002). In 1992, a number of reviews appeared: Jetzer (1992) concentrates on the astrophysical relevance of BS (in particular their relevance for explaining dark matter) while Liddle and Madsen (1992) focus on their formation. Other reviews include Straumann (1992); Lee and Pang (1992). Most recently, Mielke (2016) reviewed rotating boson stars, while Herdeiro and Radu (2015a) reviewed Kerr black holes with scalar hair.

## Solving for boson stars

In this section, we present the equations governing boson-star solutions, namely the Einstein equations for the geometry description and the Klein–Gordon equation to represent the (complex) scalar field. We refer to this coupled system as the Einstein–Klein–Gordon (EKG) equations.

The covariant equations describing boson stars are presented in Sect. 2.2, which is followed by choosing particular coordinates consistent with a $3+1$ decomposition in Sect. 2.3. A form for the potential of the scalar field is then chosen and solutions are presented in Sect. 2.4.

### Conventions

Throughout this review, Roman letters from the beginning of the alphabet $a,b,c,\ldots$ denote spacetime indices ranging from 0 to 3, while letters near the middle $i,j,k,\ldots$ range from 1 to 3, denoting spatial indices. Unless otherwise stated, we use units such that $\hbar =c=1$ so that the Planck mass becomes $M_{\mathrm {Planck}} = G^{-1/2}$. We also use the signature convention $(-,+,+,+)$ for the metric.

### The Lagrangian, evolution equations and conserved quantities

The EKG evolution equations can be derived from the action (Wald 1984)

7 $$\begin{aligned} \mathcal{S} = \int \left( \frac{1}{16 \pi G} R + \mathcal{L_M} \right) \sqrt{-g}\, d^4x \end{aligned}$$

where *R* is the Ricci scalar of the spacetime represented by the metric $g_{ab}$, and its determinant $\sqrt{-g}$. The term $\mathcal{L_M}$ describes the matter, which here is that of a complex scalar field, $\phi$

8 $$\begin{aligned} \mathcal{L_M} = - \frac{1}{2} \left[ g^{ab} \nabla _a \bar{\phi }\, \nabla _b \phi + V\left( \left| \phi \right| ^2\right) \right] , \end{aligned}$$

where $\bar{\phi }$ is the complex conjugate of the field and $V(|\phi |^2)$ is the potential depending only on the magnitude of the scalar field, consistent with the *U*(1) invariance of the field in the complex plane.

Variation of the action in Eq. (7) with respect to the metric $g^{ab}$ leads to the well-known Einstein equations

9 $$\begin{aligned}&R_{ab} - \frac{R}{2} g_{ab} = 8 \pi G T_{ab} \end{aligned}$$

10 $$\begin{aligned}&T_{ab} = \frac{1}{2} \left[ \nabla _a \bar{\phi } \, \nabla _b \phi + \nabla _a \phi \, \nabla _b \bar{\phi } \right] - \frac{1}{2} g_{ab} \left[ g^{cd} \nabla _c \bar{\phi } \, \nabla _d \phi + V\left( |\phi |^2\right) \right] ,\qquad \end{aligned}$$

where $R_{ab}$ is the Ricci tensor and $T_{ab}$ is the real stress–energy tensor. Equation (9) form a system of 10 non-linear partial differential equations for the spacetime metric components $g_{ab}$ coupled to the scalar field via the stress–energy tensor given in Eq. (10).

On the other hand, the variation of the action in Eq. (7) with respect to the scalar field $\phi$, leads to the Klein–Gordon (KG) equation

11 $$\begin{aligned} g^{ab} \nabla _a \nabla _b \phi = \frac{d V}{d |\phi |^2} \phi . \end{aligned}$$

An equivalent equation is obtained when varying the action with respect to the complex conjugate $\bar{\phi }$. The simplest potential leading to boson stars is the so-called free field case, where the potential takes the form

12 $$\begin{aligned} V( |\phi |^2 ) = m^2~ |\phi |^2, \end{aligned}$$

with *m* a parameter that can be identified with the bare mass of the field theory.

According to Noether’s theorem, the invariance of the Klein–Gordon Lagrangian in Eq. (8) under global *U*(1) transformations $\phi \rightarrow \phi e^{i \varphi }$ (such that $\delta \phi = i \phi$) implies the existence of a conserved current

13 $$\begin{aligned} J^{a} = \frac{\partial \mathcal{L_M}}{\partial (\nabla _{a} \phi )} \delta \phi + \frac{\partial \mathcal{L_M}}{\partial (\nabla _{a} \bar{\phi })} \delta \bar{\phi } = \frac{i}{2} g^{ab} \left( \bar{\phi } \, \nabla _{b} \phi - \phi \, \nabla _{b} \bar{\phi } \right) , \end{aligned}$$

satisfying the conservation law

14 $$\begin{aligned} \nabla _a J^{a} = \frac{1}{\sqrt{-g}} \partial _a \left( \sqrt{-g}\, J^a \right) = 0. \end{aligned}$$

The spatial integral of the time component of this current defines the conserved Noether charge, given by

15 $$\begin{aligned} N = \int J^0 \sqrt{-g} \, dx^3, \end{aligned}$$

which can be associated with the total number of bosonic particles (Ruffini and Bonazzola 1969). If one neglects the binding energy of the star, then the total mass can be expressed simply in terms of the bare mass as *mN*.

### The $3+1$ decomposition of the spacetime

Although the spacetime description of general relativity is very elegant, the covariant form of Einstein equations is not suitable to describe how an initial configuration evolves towards the future. It is, therefore, more intuitive to instead consider a succession of spacetime geometries, where the evolution of a given slice is given by the Einstein equations (for more detailed treatments see Alcubierre 2008; Baumgarte and Shapiro 2010; Bona et al. 2009; Gourgoulhon 2012). In order to convert the four-dimensional, covariant Einstein equations to a more intuitive “space+time” or $3+1$ decomposition, the following steps are taken:

- *specify the choice of coordinates* The spacetime is foliated by a family of spacelike hypersurfaces, which are crossed by a congruence of time lines that will determine our observers (i.e., coordinates). This congruence is described by the vector field $t^a = \alpha n^a +\beta ^a$, where $\alpha$ is the lapse function which measures the proper time of the observers, $\beta ^a$ is the shift vector that measures the displacement of the observers between consecutive hypersurfaces and $n^a$ is the timelike unit vector normal to the spacelike hypersurfaces.
- *decompose every 4D object into its 3 + 1 components* The choice of coordinates allows for the definition of a projection tensor ${\gamma ^a}_b \equiv \delta ^a_b + n^a\, n_b$. Any four-dimensional tensor can be decomposed into $3+1$ pieces using the spatial projector to obtain the spatial components, or contracting with $n^a$ for the time components. For instance, the line element can be written in a general form as
  16 $$\begin{aligned} ds^2 = - \alpha ^2\, dt^2 + \gamma _{ij} (dx^i + \beta ^i dt)\, (dx^j + \beta ^j dt). \end{aligned}$$
  The stress–energy tensor can then be decomposed into its various components as
  17 $$\begin{aligned} \tau \equiv T^{ab}\, n_a\, n_b, \quad S_i \equiv T_{ab}\, n^a\,{\gamma ^a}_i, \quad S_{ij} \equiv T_{ab}\, {\gamma ^a}_i\, {\gamma ^b}_j. \end{aligned}$$
- *write down the field equations in terms of the 3 + 1 components* Within the framework outlined here, the induced (or equivalently, the spatial 3D) metric $\gamma _{ij}$ and the scalar field $\phi$ are as yet still unknown (remember that the lapse and the shift just describe our choice of coordinates). In the original $3+1$ decomposition (ADM formulation Arnowitt et al. 1962) an additional geometrical tensor $K_{ij} \equiv -\left( 1/2 \right) \mathcal{L}_{\mathbf {n}} \gamma _{ij} = -1/\left( 2\alpha \right) \left( \partial _t-\mathcal{L}_\beta \right) \gamma _{ij}$ is introduced to describe the change of the induced metric along the congruence of observers. Loosely speaking, one can view the determination of $\gamma _{ij}$ and $K_{ij}$ as akin to the specification of a position and velocity for projectile motion. In terms of the extrinsic curvature and its trace, $\mathrm {trK} \equiv {K_i}^i$, the Einstein equations can be written as
  18 $$\begin{aligned}&{R_i}^i + \left( \mathrm {trK}\right) ^2 - {K_i}^j\, {K_j}^i = 16\, \pi \, G\, \tau \, \end{aligned}$$
  19 $$\begin{aligned}&\nabla _j\;\left( {K_i}^j - \mathrm {trK}\;{\delta _i}^j \right) = 8\, \pi \, G\, S_i \end{aligned}$$
  20 $$\begin{aligned}&\left( \partial _t - \mathcal {L}_\beta \right) K_{ij} = -\, \nabla _i \nabla _j \alpha + \alpha \nonumber \\&\quad \left( R_{ij} -2{K_i}^k {K_{jk}} + \mathrm {trK}\,K_{ij} - 8\pi G \left[ S_{ij}-{\frac{\gamma _{ij}}{2}}\left( \mathrm {trS} - \tau \right) \right] \right) \qquad \end{aligned}$$
  In a similar fashion, one can introduce a quantity $Q \equiv - \mathcal{L}_{\mathbf {n}} \phi$ for the Klein–Gordon equation which reduces it to an equation first order in time, second order in space
  21 $$\begin{aligned} \partial _t (\sqrt{\gamma }\, Q) - \partial _i (\beta ^i \sqrt{\gamma } Q) + \partial _i (\alpha \, \sqrt{\gamma }\, \gamma ^{ij}\, \partial _j \phi ) = \alpha \, \sqrt{\gamma }\, \frac{d V}{d |\phi |^2} \phi . \end{aligned}$$
- *enforce any assumed symmetries* Although the boson star is found by a harmonic ansatz for the time dependence, here we choose to retain the full time-dependence. However, a considerably simplification is provided by assuming that the spacetime is spherically symmetric. Following Lai (2004), the most general metric in this case can be written in terms of spherical coordinates as
  22 $$\begin{aligned} ds^2 = \left( - \alpha ^2 + a^2\, \beta ^2 \right) dt^2 + 2\,a^2\,\beta \,dt\,dr + a^2\, dr^2 + r^2\, b^2\, d\varOmega ^2, \end{aligned}$$
  where $\alpha (t,r)$ is the lapse function, $\beta (t,r)$ is the radial component of the shift vector and *a*(*t*, *r*), *b*(*t*, *r*) represent components of the spatial metric, with $d\varOmega ^2$ the metric of a unit two-sphere. With this metric, the extrinsic curvature only has two independent components $K^i_j = {\mathrm {diag}} \left( {K^r}_r, {K^{\theta }}_{\theta }, {K^{\theta }}_{\theta } \right)$. The constraint equations, Eqs. (18) and (19), can now be written as
  23 $$\begin{aligned}&-\frac{2}{a r b} \left\{ \partial _r \left[ \frac{\partial _r (r b)}{a} \right] + \frac{1}{r b} \left[ \partial _r \left( \frac{r b}{a} \partial _r \left( r b\right) \right) - a \right] \right\} + 4 {K^r}_r\, {K^{\theta }}_{\theta } + 2 {K^{\theta }}_{\theta }\, {K^{\theta }}_{\theta }\nonumber \\\end{aligned}$$
  24 $$\begin{aligned}&\quad = \frac{8 \pi G}{a^2} \left[ \left| \varPhi \right| ^2 + \left| \varPi \right| ^2 + a^2 V\left( |\phi |^2\right) \right] \end{aligned}$$
  25 $$\begin{aligned}&\partial _r {K^{\theta }}_{\theta } + \frac{\partial _r\left( r b\right) }{r b} \left( {K^{\theta }}_{\theta } - {K^{r}}_{r}\right) = \frac{2 \pi G}{a} \left( \bar{\varPi } \varPhi + \varPi \bar{\varPhi } \right) , \end{aligned}$$
  where we have defined the auxiliary scalar-field variables
  26 $$\begin{aligned} \varPhi \equiv \partial _r \phi , \quad \varPi \equiv \frac{a}{\alpha } \left( \partial _t \phi - \beta \partial _r \phi \right) . \end{aligned}$$
  The evolution equations for the metric and extrinsic curvature components reduce to
  27 $$\begin{aligned}&\partial _t a = \partial _r (a \beta ) - \alpha a {K^{r}}_{r} \end{aligned}$$
  28 $$\begin{aligned}&\partial _t b = \frac{\beta }{r} \partial _r (r b) - \alpha b {K^{\theta }}_{\theta } \end{aligned}$$
  29 $$\begin{aligned}&\partial _t {K^{r}}_{r} - \beta \partial _r {K^{r}}_{r} = - \frac{1}{a} \partial _r \left( \frac{\partial _r \alpha }{a} \right) \nonumber \\&\quad + \,\alpha \left\{ - \frac{2}{a r b} \partial _r \left[ \frac{\partial _r (r b)}{a} \right] + \mathrm {trK}\, {K^r}_r - \frac{4\pi \,G}{a^2} \left[ 2 |\varPhi |^2 + a^2 V(|\phi |^2) \right] \right\} \nonumber \\&\partial _t {K^{\theta }}_{\theta } - \beta \partial _r {K^{\theta }}_{\theta } = \frac{\alpha }{(r b)^2} - \frac{1}{a (r b)^2} \partial _r \left[ \frac{\alpha r b}{a} \partial _r (r b) \right] \nonumber \\&+ \,\alpha \left[ \mathrm {trK}\, {K^{\theta }}_{\theta } - 4\pi \,G V(|\phi |^2) \right] . \end{aligned}$$
  Similarly, the reduction of the Klein–Gordon equation to first order in time and space leads to the following set of evolution equations
  30 $$\begin{aligned} \partial _t \phi= & {} \beta \varPhi + \frac{\alpha }{a} \varPi \end{aligned}$$
  31 $$\begin{aligned} \partial _t \varPhi= & {} \partial _r \left( \beta \varPhi + \frac{\alpha }{a} \varPi \right) \end{aligned}$$
  32 $$\begin{aligned} \partial _t \varPi= & {} \frac{1}{(r b)^2} \partial _r \left[ (r b)^2 \left( \beta \varPi + \frac{\alpha }{a} \varPhi \right) \right] + 2 \left[ \alpha {K^{\theta }}_{\theta } - \beta \frac{\partial _r (r b)}{r b} \right] \varPi - \alpha a \frac{d V}{d |\phi |^2} \phi .\nonumber \\ \end{aligned}$$
  This set of equations, Eqs. (23)–(32), describes general, time-dependent, spherically symmetric solutions of a gravitationally-coupled complex scalar field. In the next section, we proceed to solve for the specific case of a boson star.

### Mini-boson stars

The concept of a star entails a configuration of matter which remains localized. One, therefore, looks for a localized and time-independent matter configuration such that the gravitational field is stationary and regular everywhere. As shown in Friedberg et al. (1987a), such a configuration does not exist for a real scalar field. But since the stress–energy tensor depends only on the modulus of the scalar field and its gradients, one can relax the assumption of time-independence of the scalar while retaining a time-independent gravitational field. The key is to assume a harmonic ansatz for the scalar field

33 $$\begin{aligned} \phi ( \mathbf {r},t) = \phi _0(\mathbf {r}) e^{i \omega t}, \end{aligned}$$

where $\phi _0$ is a real scalar which is the profile of the star and $\omega$ is a real constant denoting the angular frequency of the phase of the field in the complex plane.

We consider spherically symmetric, equilibrium configurations corresponding to minimal energy solutions while requiring the spacetime to be static. In Schwarzschild-like coordinates, the general, spherically symmetric, static metric can be written as

34 $$\begin{aligned} ds^2 = - \alpha \left( r\right) ^2 dt^2 + a\left( r\right) ^2 dr^2 + r^2 d\varOmega ^2, \end{aligned}$$

in terms of two real metric functions, $\alpha$ and *a*. The coordinate *r* is an areal radius such that spheres of constant *r* have surface area $4\pi r^2$. For this reason, these coordinates are often called polar-areal coordinates.

The equilibrium equations are obtained by substituting the metric of Eq. (34) and the harmonic ansatz of Eq. (33) into the spherically symmetric EKG system of Eqs. (27–32) with $\beta =0,b=1$, resulting in three first order partial differential equations (PDEs)

35 $$\begin{aligned} \partial _r a= & {} -\frac{a}{2r} \left( a^2 -1\right) + 4\pi \,G r a^3 \tau \end{aligned}$$

36 $$\begin{aligned} \partial _r \alpha= & {} \frac{\alpha }{2r} \left( a^2 -1\right) + 4\pi \,G r \alpha a^2 {S^r}_r \end{aligned}$$

37 $$\begin{aligned} \partial _r \varPhi= & {} - \left[ 1 + a^2 + 4\pi \,G r^2 a^2 \left( S^r_r - \tau \right) \right] \frac{\varPhi }{r} - \left( \frac{ \omega ^2 }{\alpha ^2} - \frac{d V}{d |\phi |^2} \right) a^2\, \phi _0.\quad \end{aligned}$$

Notice that these equations hold for any stress–energy contributions and for a generic type of self-potentials $V(|\phi |^2)$. In order to close the system of Eqs. (35–37), we still have to prescribe this potential. The simplest case admitting localized solutions is the free field case of Eq. (12) for which the potential describes a field with mass *m* and for which the equations can be written as

38 $$\begin{aligned} \partial _r a= & {} \frac{a}{2} \left\{ - \frac{a^2-1}{r} \right. \left. + 4\pi \,G r \left[ \left( \frac{\omega ^2}{\alpha ^2} + m^2\right) a^2 \phi _0^2 + \varPhi ^2 \right] \right\} , \end{aligned}$$

39 $$\begin{aligned} \partial _r \alpha= & {} \frac{\alpha }{2}\left\{ \ \frac{a^2-1}{r} \right. \left. + 4\pi \,G r \left[ \left( \frac{\omega ^2}{\alpha ^2}-m^2\right) a^2 \phi _0^2 + \varPhi ^2 \right] \right\} , \end{aligned}$$

40 $$\begin{aligned} \partial _r \varPhi= & {} - \left\{ 1 + a^2 - 4\pi \,G r^2 a^2 m^2 \phi _0^2 \right\} \frac{\varPhi }{r} - \left( \frac{\omega ^2}{\alpha ^2} - m^2 \right) \phi _0\, a^2. \end{aligned}$$

In order to obtain a physical solution of this system, we have to impose the following boundary conditions,

41 $$\begin{aligned} \phi _0\left( 0 \right)= & {} \phi _{c}, \end{aligned}$$

42 $$\begin{aligned} \varPhi \left( 0 \right)= & {} 0, \end{aligned}$$

43 $$\begin{aligned} a\left( 0 \right)= & {} 1, \end{aligned}$$

44 $$\begin{aligned} \lim _{r\rightarrow \infty }\phi _0 \left( r \right)= & {} 0, \end{aligned}$$

45 $$\begin{aligned} \lim _{r\rightarrow \infty }\alpha \left( r \right)= & {} \lim _{r\rightarrow \infty } \frac{1}{a(r)}, \end{aligned}$$

which guarantee regularity at the origin and asymptotic flatness. For a given central value of the field $\{\phi _{c}\}$, we need only to adjust the eigenvalue $\{\omega \}$ to find a solution which matches the asymptotic behavior of Eqs. (44–45). This system can be solved as a shooting problem by integrating from $r=0$ towards the outer boundary $r=r_{\mathrm {out}}$ (see Dias et al. 2016 for a review on numerical methods to find stationary gravitational solutions). Equation (39) is linear and homogeneous in $\alpha$ and one is therefore able to rescale the field consistent with Eq. (45). We can get rid of the constants in the equations by re-scaling the variables in the following manner

46 $$\begin{aligned} {\tilde{\phi }_0} \equiv \sqrt{4\pi \,G} \phi _0, \quad {\tilde{r}} \equiv m\,r, \quad {\tilde{t}} \equiv \omega \, t, \quad {\tilde{\alpha }} \equiv (m/\omega ) \alpha . \end{aligned}$$

Notice that the form of the metric in Eq. (34) resembles Schwarzschild allowing the association $a^2 \equiv (1 - 2\, M/r)^{-1}$, where *M* is the ADM mass of the spacetime. This allow us to define a more general mass aspect function

47 $$\begin{aligned} M(r,t) = \frac{r}{2} \left( 1 - \frac{1}{a^2(r,t)}\right) , \end{aligned}$$

which measures the total mass contained in a coordinate sphere of radius *r* at time *t*.

In isotropic coordinates, the spherically symmetric metric can be written as

48 $$\begin{aligned} ds^2 = - \alpha \left( R\right) ^2 dt^2 + \psi \left( R\right) ^4 \left( dR^2 + R^2 d\varOmega ^2 \right) , \end{aligned}$$

where $\psi$ is the conformal factor. A change of the radial coordinate $R=R(r)$ can transform the solution obtained in Schwarzschild coordinates into isotropic ones, in particular

49 $$\begin{aligned} R (r_{\max })= & {} \left[ \left( \frac{1 + \sqrt{a}}{2} \right) ^2 \frac{r}{a} \right] _{r_{\max }} \end{aligned}$$

50 $$\begin{aligned} \frac{dR}{dr}= & {} a \, \frac{R}{r}, \end{aligned}$$

where the first condition is the initial value to integrate the second equation backwards, obtained by imposing that far away from the boson star the spacetime resembles Schwarzschild solution. By comparing the angular metric coefficients, we also find that $\psi = \sqrt{r/R}$. Further details can be found in Appendix D of Lai’s thesis (Lai 2004).

As above, boson stars are spherically symmetric solutions of the Eqs. (38–40) with asymptotic behavior given by Eqs. (41–45). For a given value of the central amplitude of the scalar field $\phi _0(r=0) =\phi _c$, there exist configurations with some effective radius and a given mass satisfying the previous conditions for a different set of *n* discrete eigenvalues $\omega ^{(n)}$. As *n* increases, one obtains solutions with an increasing number of nodes in $\phi _0$. The configuration without nodes is the *ground state*, while all those with any nodes are excited states. As the number of nodes increases, the distribution of the mass as a function of the radius becomes more homogeneous.

As the amplitude $\phi _c$ increases, the stable configuration has a larger mass while its effective radius decreases. This trend indicates that the compactness of the boson star increases. However, at some point the mass instead decreases with increasing central amplitude. Similar to models of neutron stars (see Sect. 4 of Cook 2000), this turnaround implies a maximum allowed mass for a boson star in the ground state, which numerically was found to be $M_{\max } = 0.633\,M^2_{\mathrm {Planck}}/m$. The existence of a maximum mass for boson stars is a relativistic effect, which is not present in the Newtonian limit, while the maximum of baryonic stars is an intrinsic property.

**Fig. 2:**
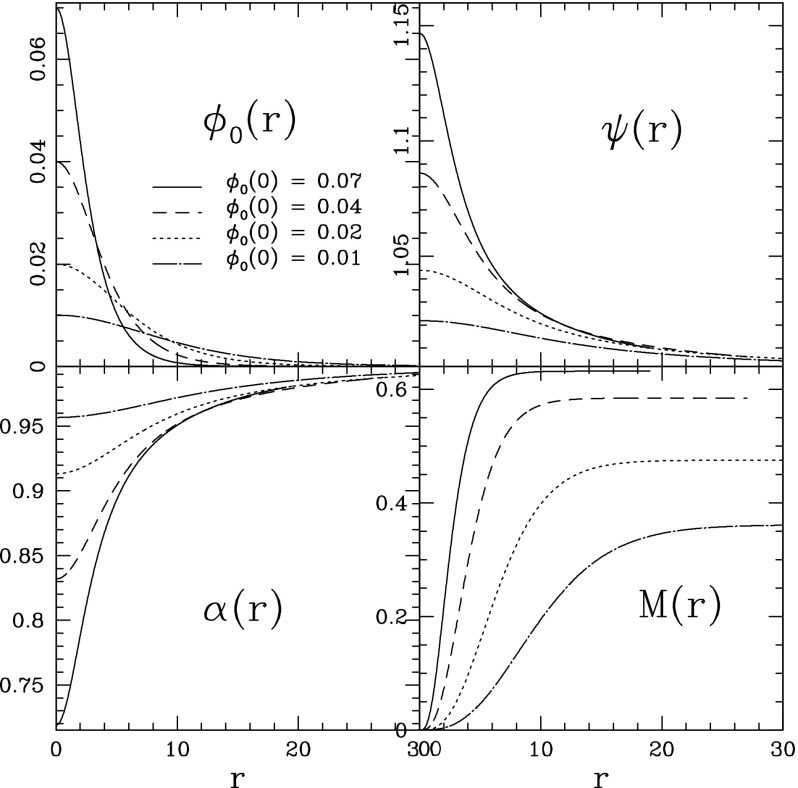
Profiles characterizing static, spherically symmetric boson stars with a few different values of the central scalar field (top left). Reprinted with permission from Lai (2004)

Solutions for a few representative boson stars in the ground state are shown in Fig. 2 in isotropic coordinates. The boson stars becomes more compact for higher values of $\phi _c$, implying narrower profiles for the scalar field, larger conformal factors, and smaller lapse functions, as the total mass increases.

## Varieties of boson stars

Quite a number of different flavours of boson stars are present in the literature. They can have charge, a fermionic component, or rotation. They can be constructed with various potentials for the scalar field. The form of gravity which holds them together can even be modified to, say, Newtonian gravity or even no gravity at all (Q-balls). To a certain extent, such modifications are akin to varying the equation of state of a normal, fermionic star. Here we briefly review some of these variations, paying particular attention to recent work.

### Self-interaction potentials

Originally, boson stars were constructed with a free-field potential without any kind of self-interaction, obtaining a maximum mass with a dependence $M \approx M^2_{\mathrm {Planck}}/m$. This mass, for typical masses of bosonic particle candidates, is much smaller than the Chandrasekhar mass $M_{\mathrm {Ch}} \approx M^3_{\mathrm {Planck}}/m^2$ obtained for fermionic stars, and so they were known as mini-boson stars. In order to extend this limit and reach astrophysical masses comparable to the Chandrasekhar mass, the potential was generalized to include a self-interaction term that provided an extra pressure against gravitational collapse. To preserve the global *U*(1) invariance, and hence to retain a conserved particle number, such a potential should be a function of $|\phi |$.

**Fig. 3:**
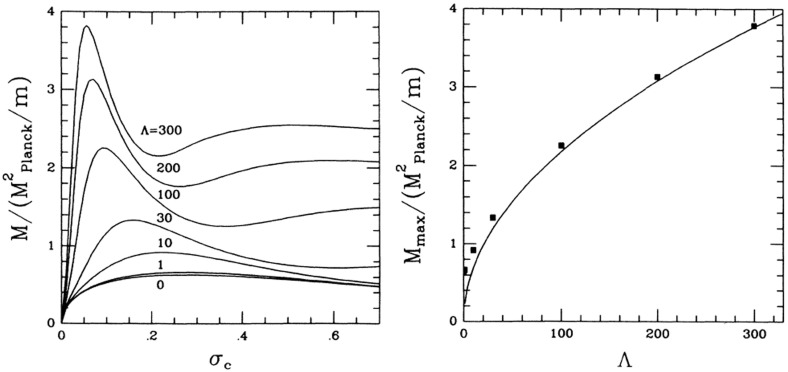
Left: The mass of the boson star as a function of the central value of the scalar field in adimensional units $\sigma _c = \sqrt{4\pi \,G} \phi _c$. Right: Maximum mass as a function of $\varLambda$ (squares) and the asymptotic $\varLambda \rightarrow \infty$ relation of Eq. (52) (solid curve). Reprinted with permission from Colpi et al. (1986); copyright by APS

**Fig. 4:**
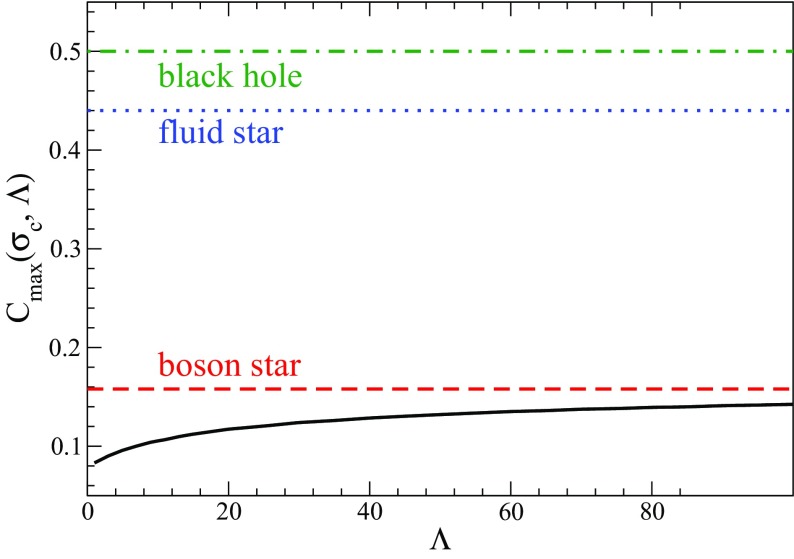
The compactness of a stable boson star (black solid line) as a function of the adimensional self-interaction parameter $\varLambda \equiv \lambda / \left( 4\pi \,G m^2\right)$. The compactness is shown for the most massive stable star (the most compact BS is unstable). This compactness asymptotes for $\varLambda \rightarrow \infty$ to the value indicated by the red, dashed line. Also shown for comparison is the compactness of a Schwarzschild BH (green dot-dashed line), and the maximum compactness of a non-spinning neutron star (blue dotted line). Reprinted with permission from Amaro-Seoane et al. (2010); copyright by IOP

Although the first expansion to nonlinear potentials was considered in Mielke and Scherzer (1981) including fourth and sixth power $|\phi |$-terms, a deeper analysis was performed later considering a potential with only the quartic term Colpi et al. (1986)

51 $$\begin{aligned} V\left( \left| \phi \right| ^2 \right) = m^2 \left| \phi \right| ^2 \, + \frac{\lambda }{2} \left| \phi \right| ^4, \end{aligned}$$

with $\lambda$ a dimensionless coupling constant. Written in terms of a general potential, the EKG equations remain the same. The families of gravitational equilibrium can be parametrized by the single dimensionless quantity $\varLambda \equiv \lambda / \left( 4\pi \,G m^2\right)$. The potential of Eq. (51) results in a maximum boson-star mass that now scales as

52 $$\begin{aligned} M_{\max } \approx 0.22 \varLambda ^{1/2} M_{\mathrm {Planck}}/m = \left( 0.1\mathrm {\ GeV}^2\right) M_{\odot } \lambda ^{1/2}/m^2 \end{aligned}$$

which is comparable to the Chandrasekhar mass for fermions with mass $m_{\mathrm {fermion}} \sim m/\lambda ^{1/4}$ (Colpi et al. 1986). This self-interaction, therefore, allows much larger masses than the mini-boson stars as long as $\varLambda \gg 1$, an inequality that may be satisfied even when $\lambda \ll 1$ for reasonable scalar boson masses. The maximum mass as a function of the central value of the scalar field is shown in Fig. 3 for different values of $\varLambda$. The compactness of the most massive stable stars was studied in Amaro-Seoane et al. (2010), finding an upper bound $M/R \lesssim 0.16$ for $\varLambda \gg 1$. Figure 4 displays this compactness as a function of $\varLambda$ along with the compactness of a Schwarzschild black hole (BH) and non-spinning neutron star for comparison. The effect of repulsive ($\lambda >0$) and attractive ($\lambda <0$) quartic terms in the self-interaction potential have been studied in Eby et al. (2016).

Many subsequent papers further analyze the EKG solutions with polynomial, or even more general non-polynomial, potentials. One work in particular (Schunck and Torres 2000) studied the properties of the galactic dark matter halos modeled with these boson stars. They found that a necessary condition to obtain stable, compact solutions with an exponential decrease of the scalar field, the series expansion of these potentials must contain the usual mass term $m^2|\phi |^2$.

More exotic ideas similarly try to include a pressure to increase the mass of BSs. Agnihotri et al. (2009) considers a form of repulsive self-interaction mediated by vector mesons within the mean-field approximation. However, the authors leave the solution of the fully nonlinear system of the Klein–Gordon and Proca equations to future work. Barranco and Bernal (2011) models stars made from the condensation of axions, using the semi-relativistic approach with two different potentials. Mathematically this approach involves averages such that the equations are equivalent to assuming the axion is constituted by a complex scalar field with harmonic time dependence.

Other generalizations of the potential allow for the presence of *nontopological soliton* solutions even in the absence of gravity, with characteristics quite different than those of the mini-boson stars. In order to obtain these solutions the potential must satisfy two conditions. First, it must be a function of $|\phi |^2$ to preserve the global *U*(1) invariance. Second, the potential should have an attractive term, bounded from below and positive for $|\phi | \rightarrow \infty$. These conditions imply a potential of at least sixth order, a condition that is satisfied by the typical degenerate vacuum form (Lee 1987; Friedberg et al. 1987b)

53 $$\begin{aligned} V\left( \left| \phi \right| ^2 \right) = m^2~ \left| \phi \right| ^2 \, \left( 1 - \frac{\left| \phi \right| ^2}{ \phi ^2_0} \right) ^2, \end{aligned}$$

for which the potential has two degenerate minima at ± $\phi _0$. The case $|\phi |=0$ corresponds to the true vacuum state, while $|\phi |=\phi _0$ represents the degenerate vacuum state.

The resulting soliton solution can be split in three different regions. When gravity is negligible, the interior solution satisfies $\phi \approx \phi _0$, followed by a shell of width 1/*m* over which $\phi$ changes from $\phi _0$ to zero, and an exterior that is essentially vacuum. This potential leads to a different scaling of the mass and radius than that of the ground state (Lee and Pang 1992)54There is another type of non-topological *soliton* star, called Q-stars (Lynn 1989), which also admits soliton solutions in the absence of gravity (i.e., Q-balls Coleman 1985; Lee and Pang 1992). The potential, besides being also a function of $|\phi |$, must satisfy the following conditions: it must behave like $\approx |\phi |^2$ near $\phi =0$, it has to be bounded $< |\phi |^2$ in an intermediate region and must be larger $> |\phi |^2$ for $|\phi | \rightarrow \infty$. The Q-stars also have three regions; an interior solution of radius $R \approx M_{\mathrm {Planck}}/\phi _0^2$, (i.e., $\phi _0 \approx m$ is the free particle inverse Compton wavelength) a very thin surface region of thickness $1/\phi _0$, and finally the exterior solution without matter, which reduces to Schwarzschild in spherical symmetry. The mass of these Q-stars scales now as $M^3_{\mathrm {Planck}}/ \phi _0^2$, and for some choices of the sixth order self-interaction potential the compactness of the boson star (defined with the expected value of *R* or $R^2$) can approach the black hole limit (Kleihaus et al. 2012). The stability of these Q-stars has been studied recently using catastrophe theory, such as Tamaki and Sakai (2010); Kleihaus et al. (2012). Rotating, axisymmetric Q-balls were constructed in Kleihaus et al. (2005, 2008). Related, rotating solutions in $2+1$ with the signum-Gordon equation instead of the KG equation are found in Arodź et al. (2009). Other interesting works have studied the formation of Q-balls by the Affleck–Dine mechanism (Kasuya and Kawasaki 2000), their dynamics in one, two and three spatial dimensions (Battye and Sutcliffe 2000), and their viability as a self-interacting dark matter candidate (Kusenko and Steinhardt 2001).

It has been shown recently that very compact boson stars can also be found by using a V-shaped potential proportional to $|\phi |$ (Hartmann et al. 2012). The same V-shaped potential with an additional quadratic massive term has been considered in Kumar et al. (2015)

Bhatt and Sreekanth (2009) considers a chemical potential to construct BSs, arguing that the effect of the chemical potential is to reduce the parameter space of stable solutions. Boson stars with a thermodynamically consistent equation of state, leading to an isotropic pressure, were considered in Chavanis and Harko (2012). The solutions, obtained by integrating the TOV equations, reached compactness smaller (but comparable) to neutron stars. The extension to boson stars with finite temperature was considered in Latifah et al. (2014).

Related work modifies the kinetic term of the action instead of the potential. Adam et al. (2010) studies the resulting BSs for a class of *K field theories*, finding solutions of two types: (i) compact balls possessing a naked singularity at their center and (ii) compact shells with a singular inner boundary which resemble black holes. Akhoury and Gauthier (2008) considers coherent states of a scalar field instead of a BS within *k-essence* in the context of explaining dark matter. Dzhunushaliev et al. (2008) modifies the kinetic term with just a minus sign to convert the scalar field to a *phantom field*. Although, a regular real scalar field has no spherically symmetric, local static solutions, they find such solutions with a real phantom scalar field.

### Newtonian boson stars

The Newtonian limit of the Einstein–Klein–Gordon Eqs. (9–11) can be derived by assuming that the spacetime metric in the weak field approximation can be written as

55 $$\begin{aligned} g_{00}= -(1+2\,V), \quad g_{ii} = 1 + 2\,V, \quad g_{ij} = 0 \quad \mathrm {for} \quad i \ne j, \end{aligned}$$

where *V* is the Newtonian gravitational potential. In this limit, the Einstein equations reduce to the Poisson equation

56 $$\begin{aligned} \nabla ^2 V= 4\pi \,G T^{00} = 4\pi \,G m^2 \phi \bar{\phi }. \end{aligned}$$

Conversely, by assuming that

57 $$\begin{aligned} \phi (x,t) \equiv \varPhi (x,t) e^{i m t}, \end{aligned}$$

in addition to the weak limit of Eq. (55), the Klein–Gordon equation reduces to

58 $$\begin{aligned} i \partial _t \varPhi = -\frac{1}{2\,m} \nabla ^2 \varPhi + m\,V\,\varPhi , \end{aligned}$$

which is just the Schrödinger equation with $\hbar =1$. Therefore, the EKG system is reduced in the Newtonian limit to the Schrödinger–Poisson (SP) system (Guenther 1995).

The initial data is obtained by solving an eigenvalue problem very similar to the one for boson stars, with similar assumptions and boundary conditions. The solutions also share similar features and display a similar behavior. A nice property of the Newtonian limit is that all the solutions can be obtained by rescaling from one known solution (Guenther 1995),

59 $$\begin{aligned} \phi _2 = \phi _1 \left( \frac{N_2}{N_1}\right) ^2, \quad \omega _2 = \omega _1 \left( \frac{N_2}{N_1}\right) ^2, \quad r_2 = r_1 \left( \frac{N_1}{N_2} \right) , \end{aligned}$$

where $N\equiv m \int dx^3 \phi \bar{\phi }$ is the Newtonian number of particles.

The possibility of including self-interaction terms in the potential was considered in Guzmán and Ureña-López (2006), studying also the gravitational cooling (i.e., the relaxation and virialization through the emission of scalar field bursts) of spherical perturbations. Non-spherical perturbations were further studied in Bernal and Guzmán (2006b), showing that the final state is a spherically symmetric configuration. Single Newtonian boson stars were studied in Guenther (1995), either when they are boosted with/without an external central potential. Rotating stars were first successfully constructed in Silveira and Sousa (1995) within the Newtonian approach, and, more recently, analytical approximate solutions for rotating boson stars in four and five dimensions has been achieved (Kan and Shiraishi 2016). Numerical evolutions of binary boson stars in Newtonian gravity are discussed in Sect. 4.2.

Recent work by Chavanis with Newtonian gravity solves the Gross–Pitaevskii equation, a variant of Eq. (58) which involves a pseudo-potential for a Bose–Einstein condensate, to model either dark matter or compact alternatives to neutron stars (Chavanis 2011, 2012, 2015; Chavanis and Harko 2012; Chavanis and Matos 2017). However, see a rebuttal to some of this work (Mukherjee et al. 2015).

Much recent work considers boson stars from a quantum perspective as a Bose–Einstein condensate involving some number, *P*, of scalar fields. Michelangeli and Schlein (2012) studies the collapse of boson stars mathematically in the mean field limit in which $P \rightarrow \infty$. Kiessling (2009) argues for the existence of *bosonic atoms* instead of stars. Bao and Dong (2011) uses numerical methods to study the mean field dynamics of BSs.

### Charged boson stars

Charged boson stars result from the coupling of the boson field to the electromagnetic field (Jetzer and van der Bij 1989). The coupling between gravity and a complex scalar field with a *U*(1) charge arises by considering the action of Eq. 7 with the following matter Lagrangian density

60 $$\begin{aligned} \mathcal{L_M} = - \frac{1}{2} \left[ g^{ab} \left( \nabla _a \bar{\phi } + i\,e\,A_a\,\bar{\phi } \right) \left( \nabla _b \phi - i\,e\,A_b\,\phi \right) + V\left( \left| \phi \right| ^2\right) \right] - \frac{1}{4} F_{ab} F^{ab}, \end{aligned}$$

where *e* is the gauge coupling constant. The Maxwell tensor $F_{ab}$ can be decomposed in terms of the vector potential $A_a$

61 $$\begin{aligned} F_{ab} = \nabla _a A_b - \nabla _b A_a. \end{aligned}$$

The system of equations obtained by performing the variations on the action forms the Einstein–Maxwell–Klein–Gordon system, which contains the evolution equations for the complex scalar field $\phi$, the vector potential $A_a$ and the spacetime metric $g_{ab}$ (Petryk 2006).

Because a charged BS may be relevant for a variety of scenarios, we detail the resulting equations. For example, *cosmic strings* are also constructed from a charged, complex scalar field and obeys these same equations. It is only when we choose the harmonic time dependence of the scalar field that we distinguish from the harmonic azimuth of the cosmic string (Vilenkin and Shellard 1994). The evolution equations for the scalar field and for the Maxwell tensor are

62 $$\begin{aligned}&g^{ab} \nabla _a \nabla _b \phi - 2\,i\,e A^a \nabla _a \phi - e^2\,\phi A_a A^a - i\, e\, \phi \, \nabla _a A^a = \frac{d V}{d |\phi |^2} \phi \end{aligned}$$

63 $$\begin{aligned}&\nabla _a F^{ab} = -J^b = i\,e\, (\bar{\phi } \nabla _b \phi - \phi \nabla _b \bar{\phi }) + 2\, e^2\, \phi \, \bar{\phi } A^b. \end{aligned}$$

Notice that the vector potential is not unique; we can still add any curl-free components without changing the Maxwell equations. The gauge freedom can be fixed by choosing, for instance, the Lorentz gauge $\nabla _a A^a=0$. Within this choice, which sets the first time derivative of the time component $A_0$, the Maxwell equations reduce to a set of wave equations in a curved background with a non-linear current. This gauge choice resembles the *harmonic gauge* condition, which casts the Einstein equations as a system of non-linear, wave equations (Wald 1984).

Either from Noether’s theorem or by taking an additional covariant derivative of Eq. (63), one obtains that the electric current $J^a$ follows a conservation law. The spatial integral of the time component of this current, which can be identified with the total charge *Q*, is conserved. This charge is proportional to the number of particles, $Q=e\,N$. The mass *M* and the total charge *Q* can be calculated by associating the asymptotic behavior of the metric with that of Reissner–Nordström metric,

64 $$\begin{aligned} g_{rr} =\left( 1 - \frac{2\,G\,M}{r} + \frac{G\,Q^2}{4\,\pi \,r^2} \right) ^{-1} \quad \mathrm {for} \quad r \rightarrow \infty , \end{aligned}$$

which is the unique solution at large distances for a scalar field with compact support.

We look for a time independent metric by first assuming a harmonically varying scalar field as in Eq. (33). We work in spherical coordinates and assume spherical symmetry. With a proper gauge choice, the vector potential takes a particularly simple form with only a single, non-trivial component $A_a=\left( A_0(r),0,0,0\right)$. This choice implies an everywhere vanishing magnetic field so that the electromagnetic field is purely electric. The boundary conditions for the vector potential are obtained by requiring that the electric field vanishes at the origin because of regularity, $\partial _r A_0 (r=0) = 0$. Because the electromagnetic field depends only on derivatives of the potential, we can use this freedom to set $A_0 (\infty ) = 0$ (Jetzer and van der Bij 1989).

With these conditions, it is possible to find numerical solutions in equilibrium as described in Jetzer and van der Bij (1989). It was shown that bound stable configurations exist only for values of the coupling constant less than or equal to a certain critical value, such that solutions are found for ${\tilde{e}}^2 \equiv e^2\,M^2_{\mathrm {Planck}}/(8\,\pi \,m^2)<1/2$. For ${\tilde{e}}^2 >1/2$ the repulsive Coulomb force is bigger than the gravitational attraction and no solutions were found, although it has been shown recently that, due to the binding energy, solutions with ${\tilde{e}}^2 =1/2$ and even slightly higher are also allowed (Pugliese et al. 2013). This bound on the BS charge in terms of its mass ensures that one cannot construct an *overcharged* BS, in analogy to the overcharged monopoles of Lue and Weinberg (2000). An overcharged monopole is one with more charge than mass and is, therefore, susceptible to gravitational collapse by accreting sufficient (neutral) mass. However, because its charge is higher than its mass, such collapse might lead to an extremal Reissner–Nordström BH, but BSs do not appear to allow for this possibility. Interestingly, Sakai and Tamaki (2012) finds that if one removes gravity, the obtained Q-balls may have no limit on their charge.

**Fig. 5:**
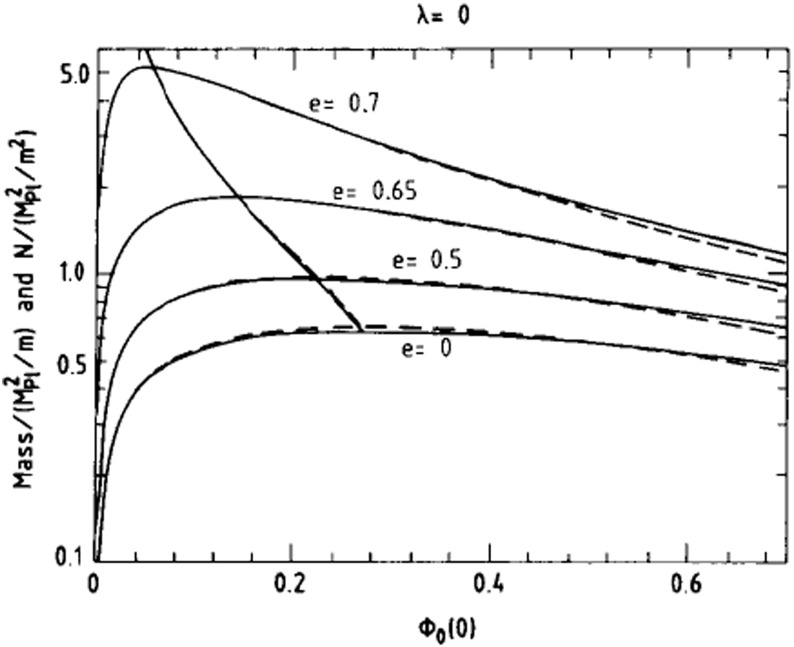
The mass (solid) and the number of particles (dashed) versus central scalar value for charged boson stars with four values of ${\tilde{e}}$ as defined in Sect. 3.3. The mostly-vertical lines crossing the four plots indicate the solution for each case with the maximum mass (solid) and maximum particle number (dashed). Reprinted with permission from Jetzer and van der Bij (1989); copyright by Elsevier

The mass and the number of particles are plotted as a function of $\phi _c$ for different values of ${\tilde{e}}$ in Fig. 5. Trivially, for ${\tilde{e}}=0$ the mini-boson stars of Sect. 2.4 are recovered. Excited solutions with nodes are qualitatively similar (Jetzer and van der Bij 1989). The stability of these objects has been studied in Jetzer (1989b), showing that the equilibrium configurations with a mass larger than the critical mass are dynamically unstable, similar to uncharged BSs.

Recent work with charged BSs includes the publication of Maple^1^ routines to study boson nebulae charge (Dariescu and Dariescu 2010; Murariu and Puscasu 2010; Murariu et al. 2008) and charged boson stars in the presence of a cosmological constant (Kumar et al. 2016). New regular solutions of charged scalar fields in a cavity are presented in Ponglertsakul et al. (2016), which are stable only when the radius of the mirror is sufficiently large.

Other work generalizes the Q-balls and Q-shells found with a certain potential, which leads to the signum-Gordon equation for the scalar field (Kleihaus et al. 2009, 2010). In particular, shell solutions can be found with a black hole in its interior, which has implications for black hole scalar hair (for a review of black hole uniqueness see Chruściel et al. 2012).

One can also consider Q-balls coupled to an electromagnetic field, a regime appropriate for particle physics. Within such a context, Eto et al. (2011) studies the chiral magnetic effect arising from a Q-ball. Other work in Brihaye et al. (2009) studies charged, spinning Q-balls.

Charged BSs in anti-de Sitter spacetimes have attracted some interest as noted at the end of Sect. 6.3.

### Oscillatons

As mentioned earlier, it is not possible to find time-independent, spacetime solutions for a real scalar field. However, there are non-singular, time-dependent near-equilibrium configurations of self-gravitating real scalar fields, which are known as *oscillatons* (Seidel and Suen 1991). These solutions are similar to boson stars, with the exception that the spacetime must also have a time dependence in order to avoid singularities.

In this case, the system is still described by the EKG Eqs. (27–32), with the the additional simplification that the scalar field is strictly real, $\phi = \bar{\phi }$. In order to find equilibrium configurations, one expands both metric components $\{A(r,t) \equiv a^2, C(r,t) \equiv (a/\alpha )^2\}$ and the scalar field $\phi (r,t)$ as a truncated Fourier series

65 $$\begin{aligned} \phi (r,t)= & {} \sum _{j=1}^{j_{\max }} \phi _{2j-1}(r) \cos \left( \left[ 2\,j-1\right] \, \omega t \right) , \end{aligned}$$

66 $$\begin{aligned} A(r,t)= & {} \sum _{j=0}^{j_{\max }} A_{2j}(r) \cos (2\, j\, \omega t), \quad C(r,t) = \sum _{j=0}^{j_{\max }} C_{2j}(r) \cos (2\, j\, \omega t), \end{aligned}$$

where $\omega$ is the fundamental frequency and $j_{\max }$ is the mode at which the Fourier series are truncated. As noted in Ureña-López et al. (2002), Alcubierre et al. (2003), the scalar field consists only of odd components while the metric terms consist only of even ones. Solutions are obtained by substituting the expansions of Eq. (65) into the spherically symmetric Eqs. (27 – 32). By matching terms of the same frequency, the system of equations reduce to a set of coupled ODEs. The boundary conditions are determined by requiring regularity at the origin and that the fields become asymptotically flat at large radius. These form an eigenvalue problem for the coefficients $\{ \phi _{2j-1}(r=0),A_{2j}(r=0),C_{2j}(r=0) \}$ corresponding to a given central value $\phi _1(r=0)$. As pointed out in Ureña-López et al. (2002), the frequency $\omega$ is determined by the coefficient $C_0(\infty )$ and is, therefore, called an *output value*. Although the equations are non-linear, the Fourier series converges rapidly, and so a small value of $j_{\max }$ usually suffices.

A careful analysis of the high frequency components of this construction reveals difficulties in avoiding infinite total energy while maintaining the asymptotically flat boundary condition (Page 2004). Therefore, the truncated solutions constructed above are not exactly time periodic. Indeed, very accurate numerical work has shown that the oscillatons radiate scalar field on extremely long time scales while their frequency increases (Fodor et al. 2010b; Grandclément et al. 2011). This work finds a mass loss rate of just one part in $10^{12}$ per oscillation period, much too small for most numerical simulations to observe. The solutions are, therefore, only near-equilibrium solutions and can be extremely long-lived.

**Fig. 6:**
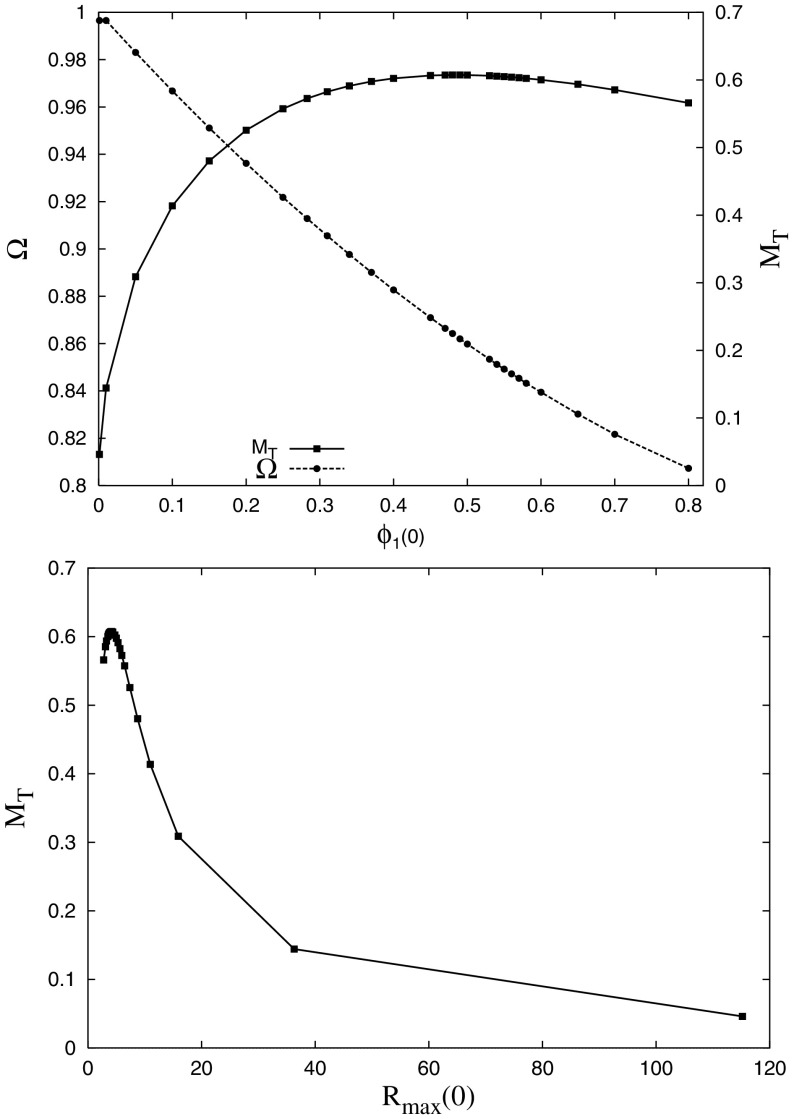
Top: Total mass (in units of $M^2_{\mathrm {Planck}}/m$) and fundamental frequency of an oscillaton as a function of the central value of the scalar field $\phi _1(r=0)$. The maximum mass is $M_{\max }=0.607\,M^2_{\mathrm {Planck}}/m$. Bottom: Plot of the total mass versus the radius at which $g_{rr}$ achieves its maximum. Reprinted with permission from Alcubierre et al. (2003); copyright by IOP

Although the geometry is oscillatory in nature, these oscillatons behave similarly to BSs. In particular, they similarly transition from long-lived solutions to a dynamically unstable branch separated at the maximum mass $M_{\max } = 0.607\,M^2_{\mathrm {Planck}}/m$. Figure 6 displays the total mass curve, which shows the mass as a function of central value. Compact solutions can be found in the Newtonian framework when the weak field limit is performed appropriately, reducing to the so-called Newtonian oscillations (Ureña-López et al. 2002). The dynamics produced by perturbations are also qualitatively similar, including gravitational cooling, migration to more dilute stars, and collapse to black holes (Alcubierre et al. 2003). More recently, these studies have been extended by considering the evolution in 3D of excited states (Balakrishna et al. 2008) and by including a quartic self-interaction potential (Valdez-Alvarado et al. 2011). In Kichenassamy (2008), a variational approach is used to construct oscillatons in a reduced system similar to that of the sine-Gordon breather solution. Such localized solutions have also been constructed in AdS (see Sect. 6.3), and numerical evolutions suggest that they are stable below some critical density (Fodor et al. 2015).

Closely related, are *oscillons* that exist in flatspace and that were first mentioned as “pulsons” in Bogolyubskiĭ and Makhan’kov (1977). And so just as a Q-ball can be thought of as a BS without gravity, an oscillon is an oscillaton in the absence of gravity. Extensive literature studies such solutions, many of which appear in Fodor et al. (2008). A series of papers establishes that oscillons similarly radiate on very long time scales (Fodor et al. 2008, 2009a, b, c). An interesting numerical approach to evolving oscillons adopts coordinates that blueshift and damp outgoing radiation of the massive scalar field (Honda 2000; Honda and Choptuik 2002). A detailed look at the long term dynamics of these solutions suggests the existence of a fractal boundary in parameter space between oscillatons that lead to expansion of a true-vacuum bubble and those that disperse (Honda 2010). Dymnikova et al. (2000) examines the collision of two of these bubbles in the context of a first order phase transition. The reheating phase of inflationary cosmology generally feature oscillons which may produce observable gravitational waves (Antusch et al. 2017; Antusch and Orani 2016).

### Rotating boson stars

Boson stars with rotation were not explored until the mid-1990s because of the lack of a strong astrophysical motivation and the technical problems with the regularization along the axis of symmetry. The first equilibrium solutions of rotating boson stars were obtained within Newtonian gravity (Silveira and Sousa 1995). In order to generate axisymmetric time-independent solutions with angular momentum, one is naturally lead to the ansatz

67 $$\begin{aligned} \phi ( \mathbf {r},t) = \phi _0(r,\theta ) e^{i ( \omega t + k \varphi )}, \end{aligned}$$

where $\phi _0(r,\theta )$ is a real scalar representing the profile of the star, $\omega$ is a real constant denoting the angular frequency of the field and *k* must be an integer so that the field $\phi$ is not multivalued in the azimuthal coordinate $\varphi$. This integer is commonly known as the rotational quantum number.

General relativistic rotating boson stars were later found (Schunck and Mielke 1996; Yoshida and Eriguchi 1997) with the same ansatz of Eq. (67). To obtain stationary axially symmetric solutions, two symmetries were imposed on the spacetime described by two commuting Killing vector fields $\xi = \partial _t$ and $\eta = \partial _{\varphi }$ in a system of adapted (cylindrical) coordinates $\{t,r,\theta ,\varphi \}$. In these coordinates, the metric is independent of *t* and $\varphi$ and can be expressed in isotropic coordinates in the Lewis–Papapetrou form

68 $$\begin{aligned} ds^2 = -f dt^2 + \frac{l}{f} \left[ g \left( dr^2 + r^2\, d\theta ^2\right) + r^2\, \sin ^2 \theta \left( d\varphi - \frac{\varOmega }{r} dt \right) ^2 \right] , \end{aligned}$$

where *f*, *l*, *g* and $\varOmega$ are metric functions depending only on *r* and $\theta$. This means that we have to solve five coupled PDEs, four for the metric and one for the Klein–Gordon equation; these equations determine an elliptic quadratic eigenvalue problem in two spatial dimensions. Near the axis, the scalar field behaves as

69 $$\begin{aligned} \lim _{r \rightarrow 0} \phi _0(r,\theta ) = r^k\, h_k(\theta ) + O(r^{r+2}), \end{aligned}$$

so that for $k>0$ the field vanishes near the axis. Note that $h_k$ is some arbitrary function different for different values of *k* but no sum over *k* is implied in Eq. (69). This implies that the rotating star solutions have toroidal level surfaces instead of spheroidal ones as in the spherically symmetric case $k=0$. In this case the metric coefficients are simplified, namely $g=1$, $\varOmega =0$ and $f=f(r)$, $l=l(r)$.

The entire family of solutions for $k=1$ and part of $k=2$ was computed using the self-consistent field method (Yoshida and Eriguchi 1997), obtaining a maximum mass $M_{\max }=1.31\,M^2_{\mathrm {Planck}}/m$. Both families were completely computed in Lai (2004) using faster multigrid methods, although there were significant discrepancies in the maximum mass, which indicates a problem with the regularity condition on the *z*-axis. The mass *M* and angular momentum *J* for stationary asymptotically flat spacetimes can be obtained from their respective Komar expressions. They can be read off from the asymptotic expansion of the metric functions *f* and $\varOmega$

70 $$\begin{aligned} f = 1 - \frac{2\,G M}{r} + \mathrm{O}\left( \frac{1}{r^2}\right) , \quad \varOmega = \frac{2\,J G}{r^2} + \mathrm{O}\left( \frac{1}{r^3}\right) . \end{aligned}$$

**Fig. 7:**
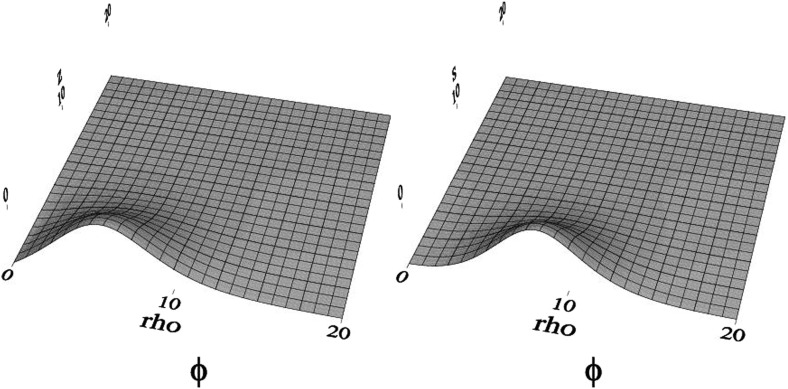
The scalar field in cylindrical coordinates $\phi (\rho ,z)$ for two rotating boson-star solutions: (left) $k=1$ and (right) $k=2$. The two solutions have roughly comparable amplitudes in scalar field. Note the toroidal shape. Reprinted with permission from Lai (2004)

Alternatively, using the Tolman expressions for the angular momentum and the Noether charge relation in Eq. 15, one obtains an important quantization relation for the angular momentum (Yoshida and Eriguchi 1997)

71 $$\begin{aligned} J = k\, N, \end{aligned}$$

for integer values of *k*. This remarkable quantization condition for this classical solution also plays a role in the work of Dias et al. (2011) discussed in Sect. 6.3. Also, Smolić (2015) discusses the quantization condition of rotating BSs in the context of symmetry. Figure 7 shows the scalar field for two different rotating BSs. Spinning BS solutions with a quartic self-interacting potential have been found too, as well as their Kerr BH limit (Herdeiro et al. 2016b).

Recently, their stability properties were found to be similar to nonrotating stars (Kleihaus et al. 2012). Rotating boson stars have been shown to develop a strong ergoregion instability when rapidly spinning on short characteristic timescales (i.e., 0.1 s–1 week for objects with mass $M=1-10^{6}\,M_{\odot }$ and angular momentum $J> 0.4\,G M^2$), indicating that very compact objects with large rotation are probably black holes (Cardoso et al. 2008). The presence of light rings (i.e., a region of space where photons are forced to travel in closed orbits) around rotating boson stars is studied in Grandclément (2016), while geodesics on the spacetime of these solutions are studied in Grandclément et al. (2014).

A recent review by Mielke (2016) focuses on rotating boson stars. Further discussion concerning the numerical methods and limitations of some of these approaches can also be found in the Ph.D. thesis by Lai (2004).

### Fermion–boson stars

The possibility of compact stellar objects made with a mixture of bosonic and fermionic matter was studied in Henriques et al. (1989, 1990a). In the simplest case, the bosonic component interacts with the fermionic component only via the gravitational field, although different couplings were suggested in Henriques et al. (1990a) and have been further explored in de Sousa et al. (1998), Pisano and Tomazelli (1996). Such a simple interaction is, at the very least, consistent with models of a bosonic dark matter coupling only gravitationally with visible matter, and the idea that such a bosonic component would become gravitationally bound within fermionic stars is arguably a natural expectation.

One can consider a perfect fluid as the fermionic component such that the stress–energy tensor takes the standard form

72 $$\begin{aligned} T^\mathrm{fluid}_{ab}= & {} (\mu + p) u_a\, u_b + p\, g_{ab} \end{aligned}$$

where $\mu$ is the energy density, *p* is the pressure of the fluid and $u_a$ its four-velocity. Such a fluid requires an equation of state to close the system of equations (see Font 2008 for more about fluids in relativity). In most work with fermion–boson stars, the fluid is described by a degenerate, relativistic Fermi gas, so that the pressure is given by the parametric equation of state of Chandrasekhar

73 $$\begin{aligned} \mu = K (\sinh t - t) \quad p = \frac{K}{3} \left[ \sinh t - 8\, \sinh \left( \frac{t}{2} \right) + 3\,t \right] , \end{aligned}$$

where $K=m_n^4/(32\,\pi ^2)$ and $m_n$ the mass of the fermion. The parameter *t* is given by

74 $$\begin{aligned} t(r) = 4 \log \left[ \frac{p_o}{m_n} + \left( 1 + \left( \frac{p_o}{m_n} \right) ^2 \right) ^{1/2}\right] , \end{aligned}$$

where $p_o$ is the maximum value of the momentum in the Fermi distribution at radius *r*.

The perfect fluid obeys relativistic versions of the Euler equations, which account for the conservation of the fluid energy and momentum, plus the conservation of the baryonic number (i.e., mass conservation). The complex scalar field representing the bosonic component is once again described by the Klein–Gordon equation. The spacetime is computed through the Einstein equations with a stress–energy tensor, which is a combination of the complex scalar field and the perfect fluid

75 $$\begin{aligned} T_{ab} = T^{\phi }_{ab} + T^{\mathrm {fluid}}_{ab}. \end{aligned}$$

After imposing the harmonic time dependence of Eq. (33) on the complex scalar field, assuming a static metric as in Eq. (34) and the static fluid $u_i=0$, one obtains the equations describing equilibrium fermion–boson configurations

$$\begin{aligned} \frac{da}{dr}= & {} \frac{a}{2}\left\{ \frac{1}{r}(1-a^{2})+4\pi \,G r\left[ \left( \frac{\omega ^{2}}{\alpha ^{2}}+m^{2}\right) a^{2}\phi ^{2}(r)+ \varPhi ^{2}(r)+2a^{2}\mu \right] \right\} \\ \frac{d\alpha }{dr}= & {} \frac{\alpha }{2}\left\{ \frac{1}{r}(a^{2}-1)+4\pi \,G r\left[ \left( \frac{\omega ^{2}}{\alpha ^{2}}-m^{2}\right) a^{2}\phi ^{2}(r)+ \varPhi ^{2}(r)+2a^{2}p\right] \right\} \\ \frac{d\phi }{dr}= & {} \varPhi (r) \\ \frac{d\varPhi }{dr}= & {} \left( m^{2}-\frac{\omega ^{2}}{\alpha ^{2}}\right) a^{2}\phi -\left[ 1+a^{2}-4\pi \,Ga^{2}r^{2}\left( m^{2}\phi ^{2}+ \mu -p\right) \right] \frac{\varPhi }{r} \\ \frac{dp}{dr}= & {} -(\mu +p)\frac{\alpha ^{'}}{\alpha }. \end{aligned}$$

These equations can be written in adimensional form by rescaling the variables and introducing the following quantities

76 $$\begin{aligned}&x\equiv mr, \quad \sigma (x)\equiv \sqrt{4\pi \,G} \phi (0,r), \quad \varOmega \equiv \omega /m^{2}, \nonumber \\&\bar{\mu }\equiv (4\pi \,G/m^{2})\mu , \quad \bar{p}\equiv (4\pi \,G/m^{2})p. \end{aligned}$$

By varying the central value of the fermion energy density $\mu (r=0)$ and the scalar field $\phi (r=0)$, one finds stars dominated by either bosons or fermions, with a continuous spectrum in between. It was shown that the stability arguments made with boson stars can be generalized to these mixed objects (Jetzer 1990).

**Fig. 8:**
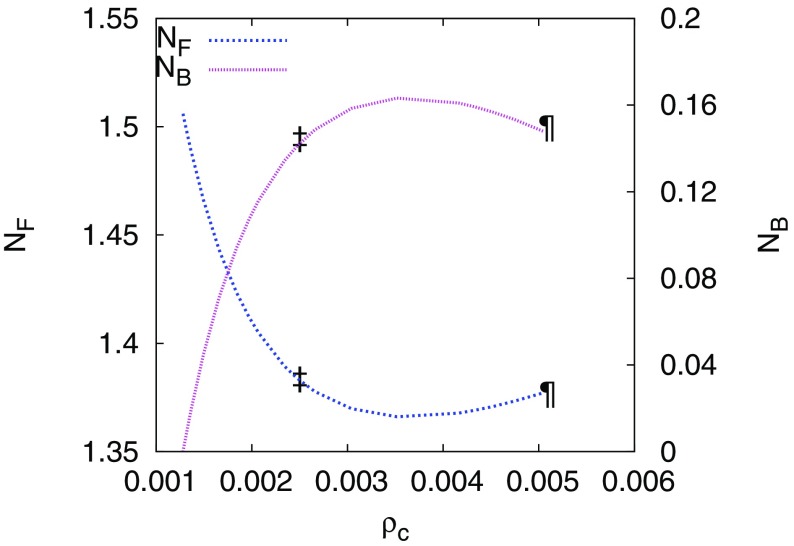
Initial data of a mixed fermion–boson star with fixed total mass $M_T= 1.4$. The numbers of fermions, $N_F$, and bosons, *N* (denoted $N_B$ in the figure, but just *N* in this text), in terms of the central density, $\rho _c$, are plotted. The position of the maximum of *N* (and correspondingly the minimum of $N_F$) represents the critical point, with a maximum value $N/N_F = 12$%, which separates the stable and the unstable solutions. The two configurations marked, one on each side of the maximum/minimum, correspond to $N/N_F \approx 10$%. Reprinted with permission from Valdez-Alvarado et al. (2013); copyright by APS

More recently, neutron stars with a bosonic component, sourced by dark matter accretion, have also been considered (Valdez-Alvarado et al. 2013; Brito et al. 2016). The fermionic matter for a cold star can be described easily by using simultaneously the polytropic and the ideal gas equation of state $P=K \rho ^{\varGamma } = (\varGamma - 1) \rho \epsilon$, where $\rho$ is the rest-mass density, $\epsilon$ its internal energy, *K* the polytropic constant, and $\varGamma$ the adiabatic index (i.e., the energy density can be written then as $\mu =\rho (1+\epsilon )$). For standard masses of the neutron star, stable configurations allow only about $N\approx 12\% N_F$, where $N_F$ is the number of fermions. Fig. 8 displays both *N* and $N_F$ for a fixed total mass but with different central densities, $\rho _c$. Similar studies have been performed by coupling fermion matter to oscillatons instead of boson stars (Brito et al. 2016).

The existence of slowly rotating fermion–boson stars was shown in de Sousa et al. (2001), although no solutions were found in previous attempts (Kobayashi et al. 1994). Also see Dzhunushaliev et al. (2011) for unstable solutions consisting of a real scalar field coupled to a perfect fluid with a polytropic equation of state.

### Multi-state boson stars

It turns out that excited BSs, as dark matter halo candidates, provide for flatter, and hence more realistic, galactic rotation curves than ground state BSs. The problem is that they are generally unstable to decay to their ground state. Combining excited states with the ground states in what are called *multi-state* BSs is one way around this.

Although bosons in the same state are indistinguishable, it is possible to construct non-trivial configurations with bosons in different excited states. A system of bosons in *P* different states that only interact with each other gravitationally can be described by the following Lagrangian density

77 $$\begin{aligned} \mathcal{L} = \frac{1}{16 \pi G} R - \sum _{n=1}^{P} \frac{1}{2} \left[ g^{ab} \partial _a \bar{\phi }^{(n)} \partial _b \phi ^{(n)} + V\left( \left| \phi ^{(n)} \right| ^2\right) \right] , \end{aligned}$$

where $\phi ^{(n)}$ is the particular complex scalar field representing the bosons in the *n*-state with $n-1$ nodes. The equations of motion are very similar to the standard ones described in Sect. 2.2, with two peculiarities: (i) there are *n* independent KG equations (i.e., one for each state) and (ii) the stress–energy tensor is now the sum of contributions from each mode. Equilibrium configurations for this system were found in Bernal et al. (2010).

In the simplest case of a multi-state boson, one has the ground state and the first excited state. Such configurations are stable if the number of particles in the ground state is larger than the number of particles in the excited state (Bernal et al. 2010; Alic 2009)

78 $$\begin{aligned} N^{(1)} \ge N^{(2)}. \end{aligned}$$

**Fig. 9:**
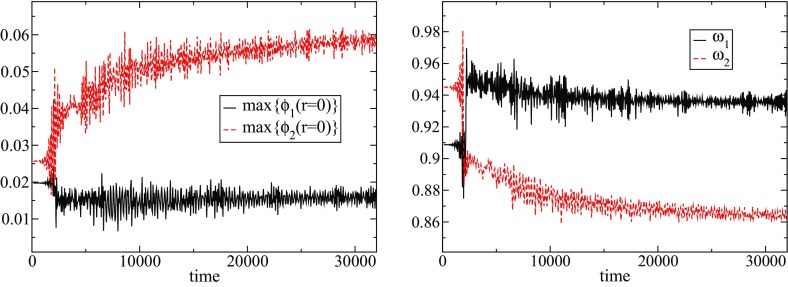
Left: The maximum of the central value of each of the two scalar fields constituting the multi-state BS for the fraction $\eta =3$, where $\eta \equiv N^{(2)}/N^{(1)}$ defines the relative “amount” of each state. Right: The frequencies associated with each of the two states of the multi-state BS. At $t=2000$, there is a flip in which the excited state (black solid) decays and the scalar field in the ground state (red dashed) becomes excited. Discussed in Sect. 3.7. Reprinted with permission from Bernal et al. (2010); copyright by APS

This result can be understood as the ground state deepens the gravitational potential of the excited state, and thereby stabilizing it. Unstable configurations migrate to a stable one via a flip-flop of the modes; the excited state decays, while the ground jumps to the first exited state, so that the condition (78) is satisfied. An example of this behavior can be observed in Fig. 9.

Similar results were found in the Newtonian limit (Ureña-López and Bernal 2010), however, with a slightly higher stability limit $N^{(1)} \ge 1.13\, N^{(2)}$. This work stresses that combining several excited states makes it possible to obtain flatter rotation curves than only with ground state, producing better models for galactic dark matter halos (see also discussion of boson stars as an explanation of dark matter in Sect. 5.4).

Hawley and Choptuik (2003) considers two scalar fields describing boson stars that are phase shifted in time with respect to each other, studying the dynamics numerically. In particular, one can consider multiple scalar fields *with* an explicit interaction (beyond just gravity) between them, say $V\left( |\phi ^{(1)}|\, |\phi ^{(2)}| \right)$. Brihaye et al. (2009), Brihaye and Hartmann (2009) construct such solutions, considering the individual particle-like configurations for each complex field as *interacting* with each other.

### Proca stars

Boson stars can be understood as condensates of massive spin 0 bosonic particles modeled by a scalar field. Recently, analogous self-gravitating solutions, made of massive spin 1 particles, have been found in the novel work of Brito et al. (2016). These configurations, modeled by a massive complex vector field $A_a$, are described by the Proca action for the matter sector

79 $$\begin{aligned} \mathcal{L_M} = - \frac{1}{4} F_{ab} {\bar{F}}^{ab} - \frac{1}{2} m^2 A_a {\bar{A}}^a, \end{aligned}$$

where *m* is the mass of the Proca field and $F_{ab}$ the field strength satisfying $F_{ab} = \nabla _a A_b - \nabla _b A_a$. The system of equations obtained by performing the variations on the action forms the Einstein–Proca system. The evolution equations for the Proca field are

80 $$\begin{aligned} \nabla _a F^{ab} = m^2 A^b, \end{aligned}$$

which implies that the Lorentz condition $\nabla _a A^a =0$ is not a gauge choice like in Maxwell equations, but instead a dynamical requirement. The Einstein equations include now the stress–energy tensor

81 $$\begin{aligned} T_{ab} = - F_{c(a} {{\bar{F}}_{b)}}^{~c} - \frac{1}{4} g_{ab} F_{cd} {\bar{F}}^{cd} + m^2 \left[ A_{(a} {\bar{A}}_{b)} - \frac{1}{2} g_{ab} A_{c} {\bar{A}}^c \right] . \end{aligned}$$

Like in the scalar case, there is a global *U*(1) invariance of the action under transformations $A_a \rightarrow e^{i\theta } A_a$, implying the existence of a conserved 4-current due to Noether’s theorem

82 $$\begin{aligned} J^a = \frac{i}{2} \left[ {\bar{F}}^{ab} A_b - F^{ab} {\bar{A}}_b \right] . \end{aligned}$$

**Fig. 10:**
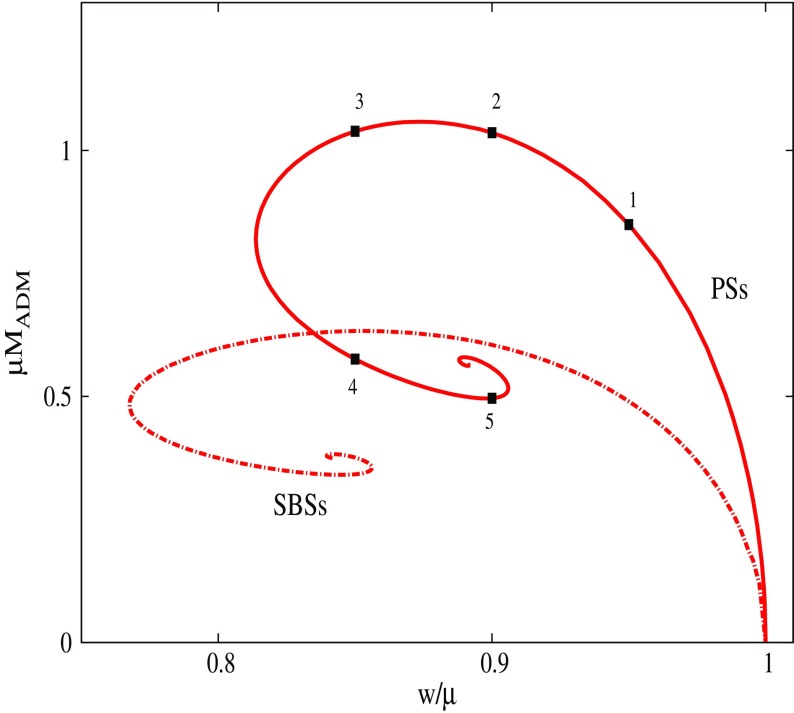
Comparison of Proca solutions with boson stars. The ADM mass of spherical Proca solutions (solid) and scalar BS solutions (dashed) are shown versus oscillation frequency. Here, the mass is expressed in terms of the field mass, $\mu$. Although the profiles are qualitatively similar, notice that the maximum mass of the Proca solutions is almost twice that of BSs. Reprinted with permission from Sanchis-Gual et al. (2017); copyright by APS

In addition to carrying a conserved Noether charge, Proca stars share many other features with boson stars. Both have a harmonic time dependence but solutions exist only for a limited range of frequencies. Both achieve a maximum ADM mass, which for Proca stars is $M_{\max }=1.058 M^2_{\mathrm {Planck}}/m$, larger, but of the same order, than those for (mini-)boson stars. Fig. 10 displays the masses of both BSs and PSs versus their (internal) oscillation frequencies. The maximum mass solution separates stable from unstable configurations. Different types of Proca stars are also possible, such as those with rotation (Brito et al. 2016), charge (Landea and García 2016), or in anti-de Sitter spacetime (Duarte and Brito 2016b). Numerical evolutions of these configurations have been performed in Sanchis-Gual et al. (2017).

### Kerr black holes with scalar hair and superradiance

Closely related to a BS, one can instead construct stable configurations of a complex scalar field around a rotating black hole (Hod 2012). Such solutions are akin to a BS with a black hole embedded at its center. As such, the scalar field serves as *scalar hair* (see a recent review about no-scalar-hair theorems by Herdeiro and Radu 2015b).

To find such solutions, one proceeds in much the same fashion as the construction of rotating solutions (Sect. 3.5). In particular, because rotation is required to achieve a stable configuration, one works in axisymmetry and assumes a harmonic ansatz for both the internal and azimuthal rotations

83 $$\begin{aligned} \phi ( \mathbf{r},t) = e^{-i \omega t} e^{i m \phi } \psi (r,\theta ). \end{aligned}$$

Here $\omega$ is the (complex) angular frequency and *m* must be an integer ($m=\pm \, 1, \pm \, 2, \dots$) for continuity in the azimuthal direction.

Instead of solving the full system of equations, a first approximation can be obtained by solving the linearized scalar field equations on a fixed spacetime (Herdeiro and Radu 2015a). Within such a linear approximation, one finds that non-rotating (Schwarzschild) BHs do not allow for bound states with strictly real $\omega$ (Herdeiro and Radu 2014b). However, *quasi-bound* states can exist with $\mathfrak {I}(\omega ) <0$ in which the scalar field decays, infalling into the BH.

For a Kerr black hole with angular momentum *J*, mass *M*, and horizon radius in the equatorial plane $r_H$, one can identify the angular velocity of the horizon as $\varOmega _H\equiv J/(2 M^2 r_H)$. For such rotating BHs, there is a critical frequency $\omega _c \equiv m \varOmega _H$ separating disparate behavior. For $\omega =\omega _c$, the frequency is strictly real allowing for regular bound states known as *scalar clouds*.

As $\omega$ increases above $\omega _c$, its imaginary part becomes negative, allowing again only for quasi-bound states with a time-decaying scalar field. In contrast, as $\omega$ decreases below $\omega _c$, its imaginary part becomes positive, indicating growth of the scalar field in Eq. (83). This growth of the massive field is called the *superradiant instability* (for a recent review of superradiance see Brito et al. 2015) and results in the extraction of energy, charge, and angular momentum from the black hole. For a rigorous treatment of this instability and a proof of boundedness see the work of Dafermos et al. (2014).

In Kühnel and Rampf (2014), an analog of a boson star (see Sect. 6.4 for physical analogs of BSs) is used to study superradiance. BSs have also been found as the zero radius limit of hairy black holes in AdS${}_4$ (see Sect. 6.3 for BSs in AdS), and these hairy BHs are proposed as the end state of the superradiant instability (Dias and Masachs 2017).

**Fig. 11:**
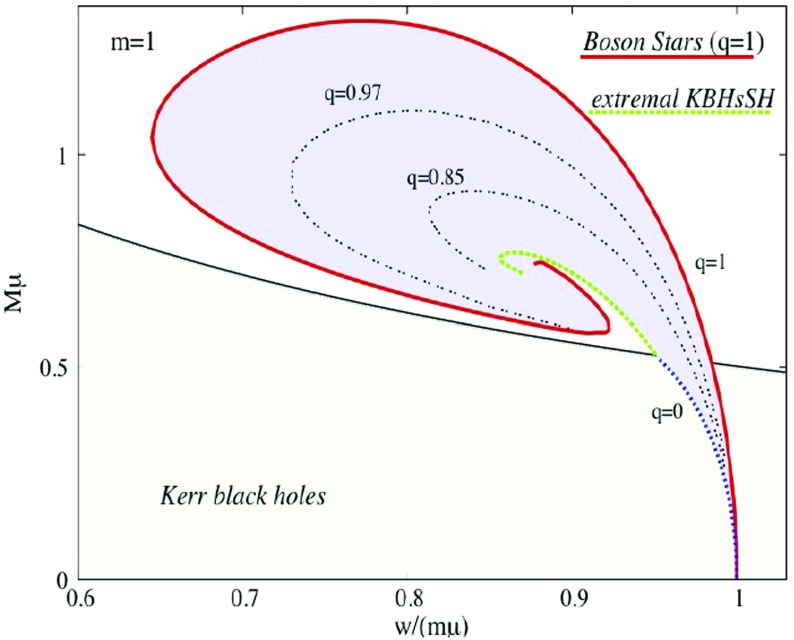
Domain of existence for hairy black holes. The ADM mass of the solutions versus the oscillation frequency of the scalar field frequency. Solutions for a range of values of *q* interpolating between Kerr ($q=0$) and BSs ($q=1$) all with azimuthal quantum number $m=1$. For $0<q<1$, solutions describe rotating BHs surrounded by a scalar cloud, constituting scalar hair for the BH. Reprinted with permission from Herdeiro and Radu (2015a); copyright by IOP

These solutions persist when solving the fully nonlinear system in which the harmonic ansatz of Eq. (83) implies that the stress–energy tensor is independent of $\{t,\phi \}$, and are generically known as *Kerr BHs with scalar hair* (Herdeiro and Radu 2014b). As reviewed by Herdeiro and Radu (2015a), solutions can be parametrized in such a way that connects pure Kerr BHs with pure BSs. In particular, defining $q \equiv kN/J$ where *N* is the number of bosonic particles as in Eq. (15) and where *k* is the integer “quantum” number associated with the angular momentum as in Eq. (71), Kerr BHs are described by the vanishing of the scalar field, $q=0$, and BSs are described by the vanishing of the horizon, $q=1$. Fig. 11 shows the space of solutions interpolating between these two limits.

More recent work has extended these solutions. For example, a self-interacting potential with a quartic term was considered in Herdeiro et al. (2015b, 2016b), producing a larger amplitude scalar field but not a more massive black hole than with the non-self-interacting potential. Coupling the scalar field to the electromagnetic field allows for charged clouds (Delgado et al. 2016). Kerr black holes with Proca hair (see Sec. 3.8 for a description of Proca stars) were constructed in Herdeiro et al. (2016a). Superradiant instabilities are likely to be weaker for hairy black holes than for Kerr black holes with the same global charge (Herdeiro and Radu 2014a). A recent review on the physical properties of Kerr black holes with scalar hair can be found in Herdeiro and Radu (2015a), and prospects for testing whether BHs have hair is reviewed in Cardoso and Gualtieri (2016).

Chodosh and Shlapentokh-Rothman (2015) study the scalar cloud solutions analytically and demonstrate existence. They also consider certain uniqueness and stability properties of solutions close to Kerr and review past analytic work in this area.

### Alternative theories of gravity

Instead of modifying the scalar field potential, one can consider alternative theories of gravity. Constraints on such theories are already significant given the great success of general relativity (Will 2014), and more strict bounds might be set with present and future astrophysical observations (Berti et al. 2015). However, the fast advance of electromagnetic observations and the rise of gravitational-wave astronomy promise much more in this area, in particular in the context of compact objects that probe strong-field gravity.

An ambitious effort is begun in Pani et al. (2011), which studies a very general gravitational Lagrangian (“extended scalar–tensor theories”) with both fluid stars and boson stars. The goal is for observations of compact stars to constrain such theories of gravity. General theoretical bounds on the mass to radius ratio of stable compact objects (i.e., both neutron and boson stars) can be set for extended gravity theories, in particular for scalar tensor theories (Burikham et al. 2016).

It has been found that scalar tensor theories allow for *spontaneous scalarization* in which the scalar component of the gravity theory transitions to a non-trivial configuration analogously to ferromagnetism with neutron stars (Damour and Esposito-Farèse 1996). Such scalarization is also found to occur in the context of boson-star evolution (Alcubierre et al. 2010) and scalarized hairy black holes (Kleihaus et al. 2015).

One motivation for alternative theories is to explain the apparent existence of dark matter without resorting to some unknown dark matter component. Perhaps the most well known of these is MOND (modified Newtonian dynamics) in which gravity is modified only at large distances (Milgrom 1983, 2011) (for a review see Famaey and McGaugh 2012). A nonminimal coupling of the scalar field to the Ricci curvature scalar results in configurations that better resemble dark energy stars than ordinary boson stars (Horvat and Marunović 2013; Marunović 2015) . Boson stars are studied within TeVeS (Tensor–Vector–Scalar), a relativistic generalization of MOND (Contaldi et al. 2008). In particular, their evolutions of boson stars develop caustic singularities, and the authors propose modifications of the theory to avoid such problems. Bosons star solutions also exist in bi-scalar extensions of Horndeski gravity (Brihaye et al. 2016), in the framework of teleparallel gravity (Horvat et al. 2015), and within conformal gravity and its scalar–tensor extensions (Brihaye and Verbin 2009, 2010). Charged boson stars with torsion-coupled field have been considered in Horvat et al. (2015).

Recently there has been renewed interest in Einstein–Gauss–Bonet theory, which appears naturally in the low energy effective action of quantum gravity models. This theory only differs from General Relativity for dimensions $D>4$, and so the easiest non-trivial case is to consider $D=5$. Boson star have been found in ($4+1$)-dimensional Gauss–Bonnet gravity (Hartmann et al. 2013). Rotating configurations were constructed in Brihaye and Riedel (2014), and its classical instability and existence of ergoregions studied in Brihaye and Hartmann (2016). Rotating boson stars in odd-dimensional asymptotically anti-de Sitter spacetimes in Einstein–Gauss–Bonnet gravity are studied in Henderson et al. (2015). A non-minimal coupling between a complex scalar field and the Gauss–Bonnet term was studied in Baibhav and Maity (2017). Coupling Einstein gravity to a complex self-interacting boson field as well as a phantom field allows for new type of configurations, namely boson stars harboring a wormhole at their core (Dzhunushaliev et al. 2014).

### Gauged boson stars

In 1988, Bartnik and McKinnon published quite unexpected results showing the existence of particle-like solutions within *SU*(2) Yang–Mills coupled to gravity (Bartnik and McKinnon 1988). These solutions, although unstable, were unexpected because no particle-like solutions are found in either the Yang–Mills or gravity sectors in isolation. Recall also that no particle-like solutions were found with gravity coupled to electromagnetism in early efforts to find Wheeler’s geon (however, see Sect. 6.3 for discussion of Dias et al. (2012), which finds geons within AdS).

Bartnik and McKinnon generalize from the Abelian *U*(1) gauge group to the non-Abelian *SU*(2) group and thereby find these unexpected particle-like solutions. One can consider, as does Schunck and Mielke (2003) (see Sect. IIp), these globally regular solutions (and their generalizations to *SU*(*n*) for $n>2$) as *gauged boson stars* even though these contain no scalar field. One can instead explicitly include a scalar field doublet coupled to the Yang–Mills gauge field (Brihaye et al. 2005) as perhaps a more direct generalization of the (*U*(1)) charged boson stars discussed in Sect. 3.3.

Dzhunushaliev et al. (2007) studies BSs formed from a gauge condensate of an *SU*(3) gauge field, and Brihaye and Verbin (2010) extends the Bartnik–McKinnon solutions to conformal gravity with a Higgs field.

## Dynamics of boson stars

In this section, the formation, stability and dynamical evolution of boson stars are discussed. One approach to the question of stability considers small perturbations around an equilibrium configuration, so that the system remains in the linearized regime. Growing modes indicate instability. However, a solution can be linearly stable and yet have a nonlinear instability. One example is Minkowski space, which, under small perturbations, relaxes back to flat, but, for sufficiently large perturbations, leads to black-hole formation, decidedly not Minkowski. To study nonlinear stability, other methods are needed. In particular, full numerical evolutions of the Einstein–Klein–Gordon (EKG) equations are quite useful for understanding the dynamics of boson stars.

### Gravitational stability

A linear stability analysis consists of studying the time evolution of infinitesimal perturbations about an equilibrium configuration, usually with the additional constraint that the total number of particles must be conserved. In the case of spherically symmetric, fermionic stars described by a perfect fluid, it is possible to find an eigenvalue equation for the perturbations that determines the normal modes and frequencies of the radial oscillations (see, for example, Font et al. 2002). Stability theorems also allow for a direct characterization of the stability branches of the equilibrium solutions (Friedman et al. 1988; Cook et al. 1994). Analogously, one can write a similar eigenvalue equation for boson stars and show the validity of similar stability theorems. In addition to these methods, the stability of boson stars has also been studied using mainly two other, independent methods: by applying catastrophe theory and by solving numerically the time dependent Einstein–Klein–Gordon equations. Recently, a method utilizing information theory shows promise in analyzing the stability of equilibrium configurations. All these methods agree with the results obtained in the linear stability analysis.

#### Linear stability analysis

Assume that a spherically symmetric boson star in an equilibrium configuration is perturbed only in the radial direction. The equations governing these small radial perturbations are obtained by linearizing the system of equations in the standard way; expand the metric and the scalar field functions to first order in the perturbation and neglect higher order terms in the equations (Gleiser 1988; Jetzer 1989a). Considering the collection of fields for the system $f_i$, one expands them in terms of the background solution ${}^0 f_i$ and perturbation as

84 $$\begin{aligned} f_i(r,t) = {}^0 f_i(r) + {}^1 f_i(r) e^{i \sigma t}, \end{aligned}$$

which assumes harmonic time dependence for the perturbation. Substitution of this expansion into the system of equations then provides a linearized system, which reduces to a set of coupled equations that determines the spectrum of modes ${}^1 f_i$ and eigenvalues $\sigma ^2$

85 $$\begin{aligned} L_{ij} {}^1 f_i = \sigma ^2\, M_{ij} \, {}^1 f_i, \end{aligned}$$

where $L_{ij}$ is a differential operator containing partial derivatives and $M_{ij}$ is a matrix depending on the background equilibrium fields ${}^0 f_i$. Solving this system, known as the pulsation equation, produces the spectrum of eigenmodes and their eigenvalues $\sigma$. Recently, several powerful techniques have been introduced to compute the quasinormal modes of compact objects in complicated configurations, such as in the presence of interacting fields (Macedo et al. 2016).

The stability of the star depends crucially on the sign of the smallest eigenvalue. Because of time reversal symmetry, only $\sigma ^2$ enters the equations (Lee and Pang 1989), and we label the smallest eigenvalue $\sigma _0^2$. If it is negative, the eigenmode grows exponentially with time and the star is unstable. On the other hand, for positive eigenvalues the configuration has no unstable modes and is therefore stable. The critical point at which the stability transitions from stable to unstable therefore occurs when the smallest eigenvalue vanishes, $\sigma _0 = 0$.

Equilibrium solutions of nonrotating BSs can be parametrized with a single variable, such as the central value of the scalar field $\phi _c$. We can therefore write the mass and particle number as $M=M(\phi _c)$ and $N=N(\phi _c)$, and stability theorems indicate that transitions between stable and unstable configurations occur only at the critical points in the parameter space such that

86 $$\begin{aligned} \frac{dM}{d\phi _c} = \frac{dN}{d\phi _c} = 0. \end{aligned}$$

These transitions in stability are completely analogous to those for neutron stars (Cook et al. 1994; Friedman et al. 1988; Harrison et al. 1965; Straumann 1984).

One can generalize this result for fermion–boson stars which contain a number of fermions, $N_F$, in addition to some number of bosons, *N* (see Sect. 3.6 for a discussion of fermion–boson stars). In particular, one looks for critical points in a higher dimensional parameter space by considering a vector of perturbations, $\mathbf {n}$ in a space spanned by the total mass at infinity, *M*, and the two particle numbers, *N* and $N_F$. Following Henriques et al. (1990b), the critical points are such that the directional derivatives vanish

87 $$\begin{aligned} \left. \frac{d M}{d \mathbf {n}}\right| _{b} = \left. \frac{d N}{d \mathbf {n}} \right| _{b} = \left. \frac{d N_F}{d \mathbf {n}} \right| _{b} = 0 \end{aligned}$$

where the subscript *b* means the value of the quantities at the critical point. The direction $\mathbf {n}$ at the stability boundary is tangential to the level curves of constant *M* and *N*; formally speaking, the direction $\mathbf {n}$ is orthogonal to the gradient of the functions at the boundary, $\mathbf {n} \perp \nabla (M , N, N_F )|_b$.

The condition expressed by Eq. 87 reduces to the stability condition of Eq. 86 when applied to single parameter solutions, but it allows for multi-parameter critical curves. Following the analysis of the fermion–boson star, the condition 87 implies that the equilibrium critical configurations manifest themselves at the extreme values of the number of particles when surveyed along a level curve of constant total mass (Valdez-Alvarado et al. 2013; Brito et al. 2016), namely

88 $$\begin{aligned} \left. \frac{\partial N}{\partial \rho _c}\right| _{M=\mathrm {constant}} = \left. \frac{\partial N_F}{\partial \rho _c} \right| _{M=\mathrm {constant}} = 0, \left. \frac{\partial N}{\partial \phi _c}\right| _{M=\mathrm {constant}} = \left. \frac{\partial N_F}{\partial \phi _c} \right| _{M=\mathrm {constant}} = 0,\nonumber \\ \end{aligned}$$

where $\rho _c$ is the central density of the fermionic component.

Linear perturbation analysis provides a more detailed picture such as the growth rates and the eigenmodes of the perturbations. For instance, Macedo et al. (2013a) studies the free oscillation spectra of different types of boson stars via perturbation theory.

Gleiser and Watkins (1989) carries out such an analysis for perturbations that conserve mass and charge. They find the first three perturbative modes and their growth rates, and they identify at which precise values of $\phi _c$ these modes become unstable. Starting from small values, they find that ground state BSs are stable up to the critical point of maximum mass. Further increases in the central value subsequently encounter additional unstable modes. This same type of analysis applied to excited state BSs showed that the same stability criterion applies for perturbations that conserve the total particle number (Jetzer 1989c). For more general perturbations that do not conserve particle number, excited states are generally unstable to decaying to the ground state.

A more involved analysis by Lee and Pang (1989) uses a Hamiltonian formalism to study BS stability. Considering first order perturbations that conserve mass and charge ($\delta N = 0$), their results agree with those of Gleiser and Watkins (1989); Jetzer (1989c). However, they extend their approach to consider more general perturbations which do *not* conserve the total number of particles (i.e., $\delta N \ne 0$). To do so, they must work with the second order quantities. They found complex eigenvalues for the excited states that indicate that *excited state boson stars are unstable*. More detail and discussion on the different stability analysis can be found in Jetzer (1992).

*Catastrophe theory* is part of the study of dynamical systems that began in the 1960s and studies large changes in systems resulting from small changes to certain important parameters (for a physics-oriented review see Stewart 1982). Its use in the context of boson stars is to evaluate stability, and to do so one constructs a series of solutions in terms of a limited and appropriate set of parameters. Under certain conditions, such a series generates a curve smooth everywhere except for certain points. Within a given smooth expanse between such singular points, the solutions share the same stability properties. In other words, bifurcations occur at the singular points so that solutions after the singularity gain an additional, unstable mode. Much of the recent work in this area confirms the previous conclusions from linear perturbation analysis (Tamaki and Sakai 2010, 2011a, b, c) and from earlier work with catastrophe theory (Kusmartsev et al. 1991). Another recent work using catastrophe theory finds that rotating stars share a similar stability picture as nonrotating solutions (Kleihaus et al. 2012). However, only fast spinning stars are subject to an ergoregion instability (Cardoso et al. 2008).

A recent and promising alternative method to determine the stability bounds of self-gravitating astrophysical objects, and in particular of boson stars, makes use of a new measure of shape complexity known as configurational entropy (Gleiser and Jiang 2015). Their results for the critical stability region agree with those of traditional perturbation methods with an accuracy of a few percent or better.

#### Non-linear stability of single boson stars

The dynamical evolution of spherically symmetric perturbations of boson stars has also been studied by solving numerically the Einstein–Klein–Gordon equations (Sect. 2.3), or its Newtonian limit (Sect. 3.2), the Schrödinger–Poisson system. The first such work was Seidel and Suen (1990) in which the stability of the ground state was studied by considering finite perturbations, which may change the total mass and the particle number (i.e., $\delta N \ne 0$ and $\delta M \ne 0$). The results corroborated the linear stability analysis in the sense that they found a stable and an unstable branch with a transition between them at a critical value, $\phi _{\mathrm {crit}}$, of the central scalar field corresponding to the maximal BS mass $M_{\max }=0.633\,M^2_{\mathrm {Planck}}/m$.

**Fig. 12:**
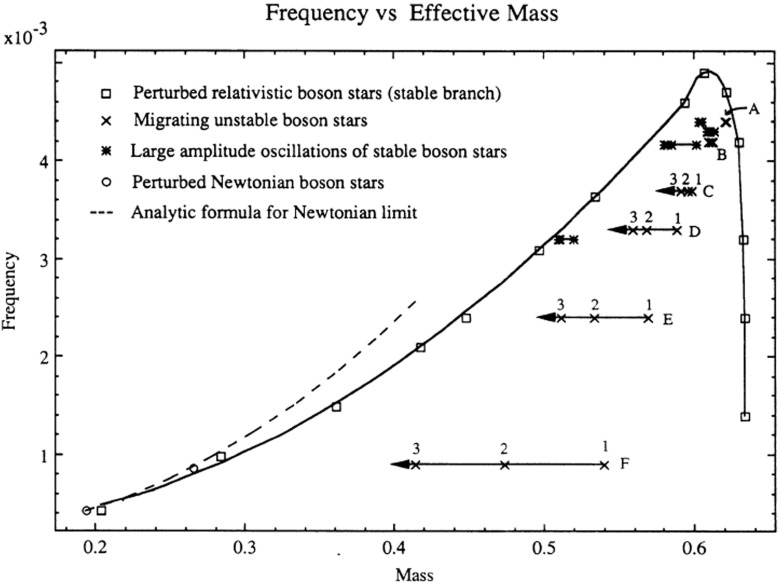
Oscillation frequencies of various boson stars are plotted against their mass. Also shown are the oscillation frequencies of unstable BSs obtained from the fully nonlinear evolution of the dynamical system. Unstable BSs are observed maintaining a constant frequency as they approach a stable star configuration. Reprinted with permission from Seidel and Suen (1990); copyright by APS

The perturbed configurations of the stable branch may oscillate and emit scalar radiation maintaining a characteristic frequency $\nu$, eventually settling into some other stable state with less mass than the original. This characteristic frequency can be approximated in the non-relativistic limit as (Seidel and Suen 1990)

89 $$\begin{aligned} \nu = \frac{\pi }{4 m R^2} - \frac{m G M}{2 \pi R}, \end{aligned}$$

where *R* is the effective radius of the star and *M* its total mass. Scalar radiation is the only damping mechanism available because spherical symmetry does not allow for gravitational radiation and because the Klein–Gordon equation has no viscous or dissipative terms. This process was named *gravitational cooling*, and it is extremely important in the context of formation of compact bosonic objects (Seidel and Suen 1994) (see below). The behavior of perturbed solutions can be represented on a plot of frequency versus effective mass as in Fig. 12. Perturbed stars will oscillate with a frequency below its corresponding solid line and they radiate scalar field to infinity. As they do so, they lose mass by oscillating at constant frequency, moving leftward on the plot until they settle on the stable branch of (unperturbed) solutions.

The perturbed unstable configurations will either collapse to a black hole or migrate to a stable configuration, depending on the nature of the initial perturbation. If the density of the star is increased, it will collapse to a black hole. On the other hand, if it is decreased, the star explodes, expanding quickly as it approaches the stable branch. Along with the expansion, energy in the form of scalar field is radiated away, leaving a very perturbed stable star, less massive than the original unstable one.

**Fig. 13:**
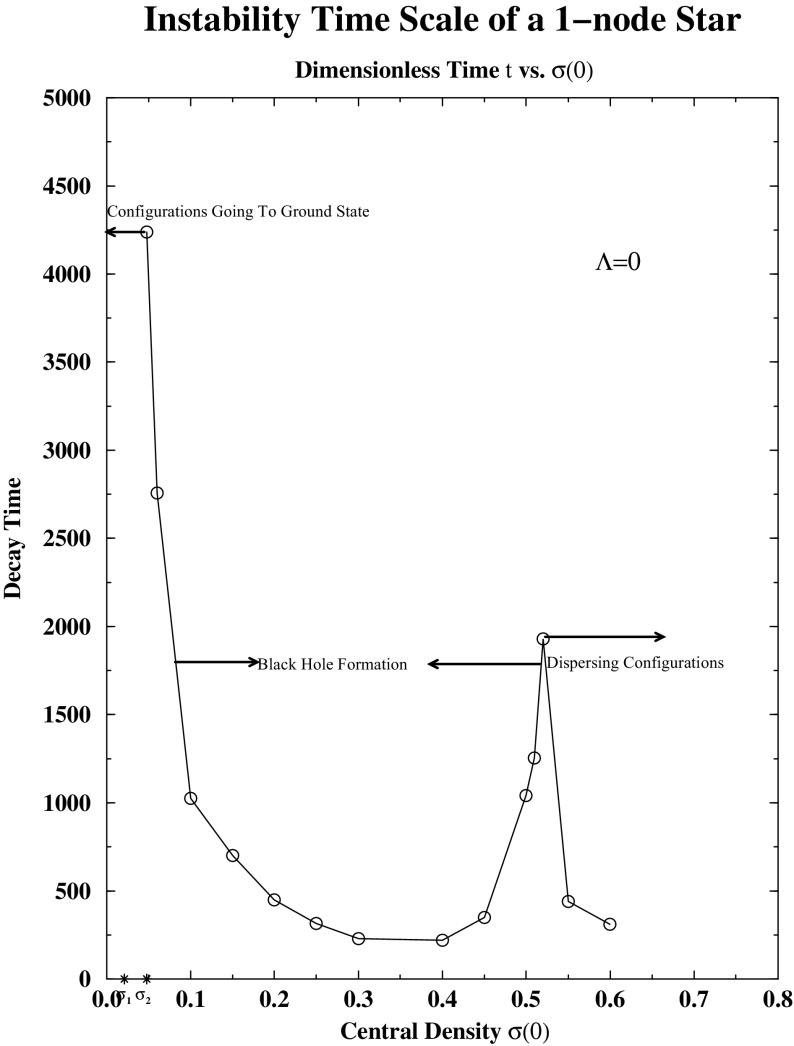
The instability time scale of an excited boson star (the first excitation) to one of three end states: (i) decay to the ground state, (ii) collapse to a black hole, or (iii) dispersal. Reprinted with permission from Balakrishna et al. (1998); copyright by APS

This analysis was extended to boson stars with self-interaction and to excited BSs in Balakrishna et al. (1998), showing that both branches of the excited states were intrinsically unstable under generic perturbations that do not preserve *M* and *N*. The low density excited stars, with masses close to the ground state configurations, will evolve to ground state boson stars when perturbed. The more massive configurations form a black hole if the binding energy $E_B = M - N m$ is negative, through a cascade of intermediate states. The kinetic energy of the stars increases as the configuration gets closer to $E_B=0$, so that for positive binding energies there is an excess of kinetic energy that tends to disperse the bosons to infinity. These results are summarized in Fig. 13, which shows the time scale of the excited star to decay to one of these states.

More recently, the stability of the ground state was revisited with 3D simulations using a Cartesian grid (Guzmán 2004). The Einstein equations were written in terms of the BSSN formulation (Shibata and Nakamura 1995; Baumgarte and Shapiro 1999), which is one of the most commonly used formulations in numerical relativity. Intrinsic numerical error from discretization served to perturb the ground state for both stable and unstable stars. It was found that unstable stars with negative binding energy would collapse and form a black hole, while ones with positive binding energy would suffer an excess of kinetic energy and disperse to infinity.

That these unstable stars would disperse, instead of simply expanding into some less compact stable solution, disagrees with the previous results of Seidel and Suen (1990), and was subsequently further analyzed in Guzmán (2009) in spherical symmetry with an explicit perturbation (i.e., a Gaussian shell of particles, which increases the mass of the star around 0.1%). The spherically symmetric results corroborated the previous 3D calculations, suggesting that the slightly perturbed configurations of the unstable branch have three possible endstates: (i) collapse to BH, (ii) migration to a less dense stable solution, or (iii) dispersal to infinity, dependent on the sign of the binding energy.

**Fig. 14:**
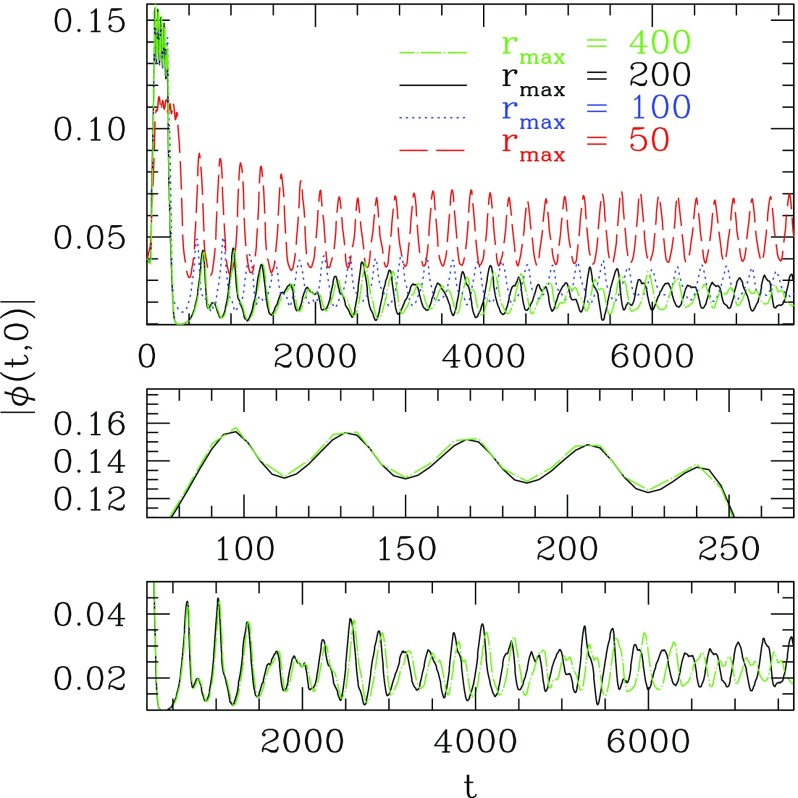
Very long evolutions of a perturbed, slightly sub-critical, boson star with differing outer boundaries. The central magnitude of the scalar field is shown. At early times ($t<250$ and the middle frame), the boson star demonstrates near-critical behavior with small-amplitude oscillations about an unstable solution. For late times ($t>250$), the solution appears converged for the largest two outer boundaries and suggests that sub-critical boson stars are *not* dispersing. Instead, they execute large amplitude oscillations about low-density boson stars. Reprinted with permission from Lai and Choptuik (2007)

Closely related is the work of Lai and Choptuik (2007) studying BS critical behavior (discussed in Sect. 6.1). They tune perturbations of boson stars so that dynamically the solution approaches some particular unstable solution for some finite time. They then study evolutions that ultimately do not collapse to BH, so-called sub-critical solutions, and find that they do not disperse to infinity, instead oscillating about some less compact, stable star. They show results with increasingly distant outer boundary that suggest that this behavior is not a finite-boundary-related effect (reproduced in Fig. 14). They use a different form of perturbation than Guzmán (2009), and, being only slightly subcritical, may be working in a regime with non-positive binding energy. However, it is interesting to consider that if indeed there are three distinct end-states, then one might expect critical behavior in the transition among the different pairings. Non-spherical perturbations of boson stars have been studied numerically in Balakrishna et al. (2006) with a 3D code to analyze the emitted gravitational waves.

The dynamics of non-standard boson stars have also been studied through numerical simulations in different scenarios. Boson stars in scalar–tensor theories of gravity were considered in Ruiz et al. (2012), focusing on the study of spontaneous and induced scalarization. Evolutions of fermion–boson stars have confirmed their stability properties and have found the normal modes of oscillations of neutron stars with a dark matter component (Valdez-Alvarado et al. 2013). Nonlinear evolutions of scalar clouds around black holes have been considered in Okawa (2015). Very recently, spherical Proca stars (see Sect. 3.8 for a discussion of such stars) have also been studied numerically (Sanchis-Gual et al. 2017), confirming that the evolutions of unstable solutions lead to outcomes analogous to those of boson stars (i.e., migration to the stable branch, total dispersion of the scalar field, or collapse to a black hole).

Much less is known about rotating BSs, which are more difficult to construct and to evolve because they are, at most, axisymmetric, not spherically symmetric. However, as mentioned in Sect. 3.5, they appear to have both stable and unstable branches (Kleihaus et al. 2012) and are subject to an ergoregion instability at high rotation rates (Cardoso et al. 2008). To our knowledge, no one has evolved rotating BS initial data. However, as discussed in the next section, simulations of mini BS binaries (Mundim 2010; Palenzuela et al. 2008) have found rotating boson stars as a result of merger.

**Fig. 15:**
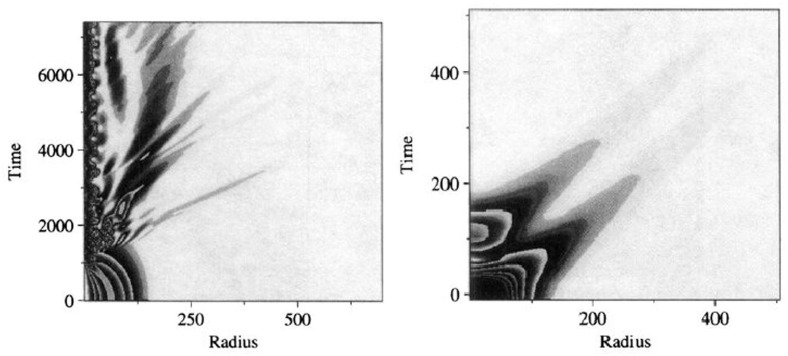
The evolution of $r^2 \rho$ (where $\rho$ is the energy density of the complex scalar field) with massive field (left) and massless (right). In the massive case, much of the scalar field collapses and a perturbed boson star is formed at the center, settling down by gravitational cooling. In the massless case, the scalar field bounces through the origin and then disperses without forming any compact object. Reprinted with permission from Seidel and Suen (1994); copyright by APS

The issue of formation of boson stars has been addressed in Seidel and Suen (1994) by performing numerical evolutions of the EKG system with different initial Gaussian distributions describing unbound states (i.e., the kinetic energy is larger than the potential energy). Quite independent of the initial condition, the scalar field collapses and settles down to a bound state by ejecting some of the scalar energy during each bounce. The ejected scalar field carries away excess and ever-decreasing amounts of kinetic energy, as the system becomes bounded. After a few free-fall times of the initial configuration, the scalar field has settled into a perturbed boson star on the stable branch. This process is the already mentioned *gravitational cooling*, and allows for the formation of compact soliton stars (boson stars for complex scalar fields and oscillatons for real scalar fields). Although these evolutions assumed spherical symmetry, which does not include important processes such as fragmentation or the formation of pancakes, they demonstrate the feasibility of the formation mechanism; clouds of scalar field will collapse under their own self-gravity while shedding excess kinetic energy. The results also confirm the importance of the mass term in the potential. By removing the massive term in the simulations, the field collapses, rebounds and completely disperses to infinity, and no compact object forms. The evolution of the scalar field with and without the massive term is displayed in Fig. 15.

### Dynamics of binary boson stars

The dynamics of binary boson stars is sufficiently complicated that it generally requires numerical solutions. The necessary lack of symmetry and the resolution requirement dictated by the harmonic time dependence of the scalar field combine so that significant computational resources must be expended for such a study. However, boson stars serve as simple proxies for compact objects without the difficulties (shocks and surfaces) associated with perfect fluid stars, and, as such, binary BS systems have been studied in the two-body problem of general relativity. When sufficiently distant from each other, the precise structure of the star should be irrelevant as suggested by Damour’s “effacement theorem” (Damour 1987).

First attempts at binary boson-star simulations assumed the Newtonian limit, since the SP system is simpler than the EKG one. Numerical evolutions of Newtonian binaries showed that in head-on collisions with small velocities, the stars merge forming a perturbed star (Choi 1998). With larger velocities, they demonstrate solitonic behavior by passing through each other, producing an interference pattern during the interaction but roughly retaining their original shapes afterwards (Choi 2002). Choi (1998) simulated coalescing binaries, although the lack of resolution in these 3D simulations did not allow for strong conclusions.

**Fig. 16:**
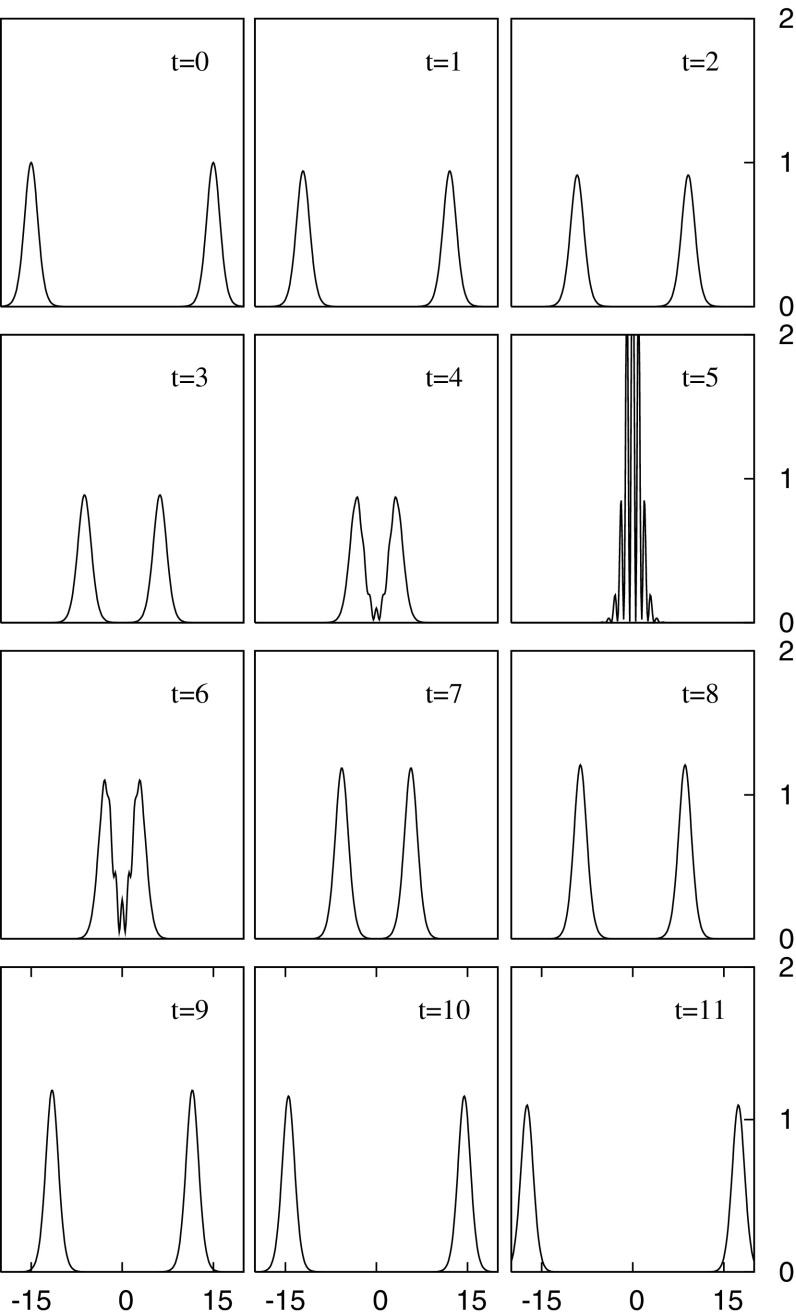
Collision of identical boson stars with large kinetic energy in the Newtonian limit. The total energy (i.e., the sum of kinetic, gravitational and self-interaction) is positive and the collision displays solitonic behavior. Contrast this with the gravity-dominated collision displayed in Fig. 17. Reprinted with permission from Bernal and Guzmán (2006a); copyright by APS

**Fig. 17:**
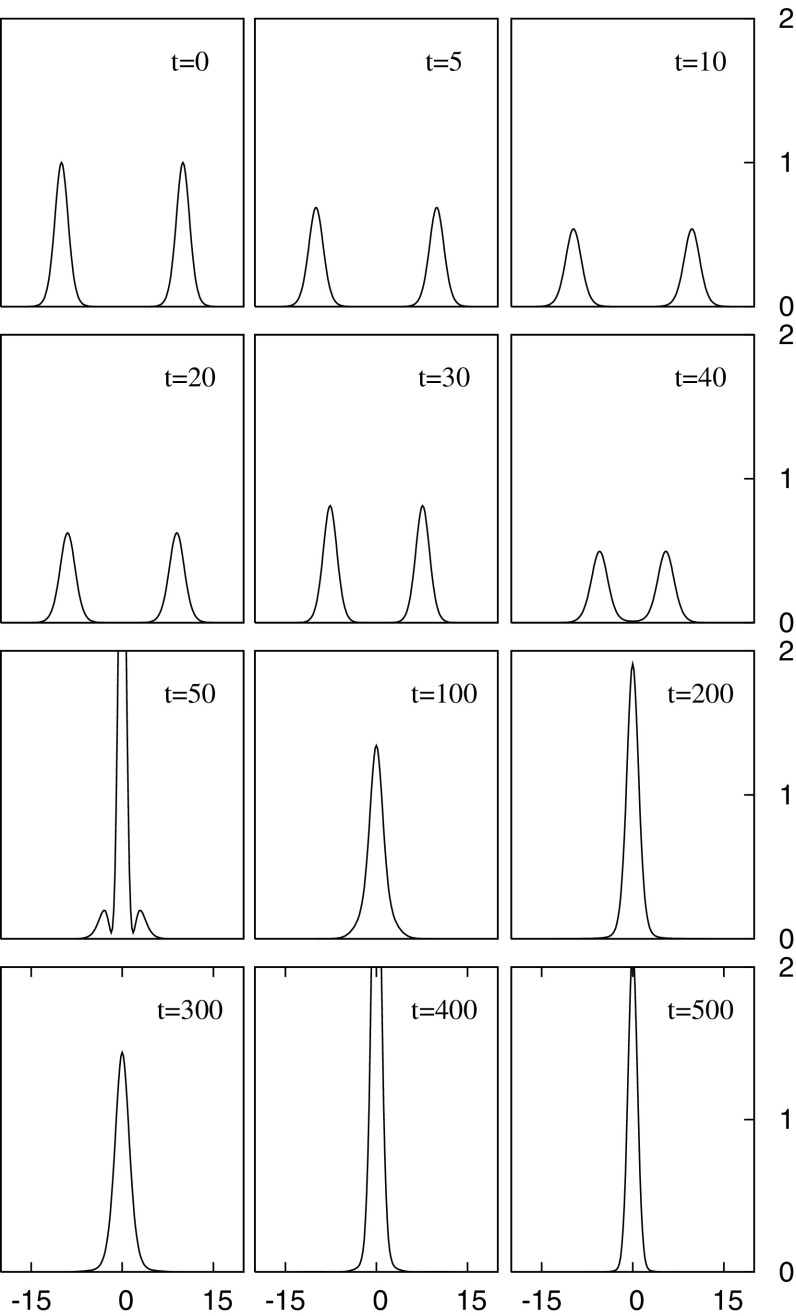
Collision of identical boson stars with small kinetic energy in the Newtonian limit. The total energy is dominated by the gravitational energy and is therefore negative. The collision leads to the formation of a single, gravitationally bound object, oscillating with large perturbations. This contrasts with the large kinetic energy case (and therefore positive total energy) displayed in Fig. 16. Reprinted with permission from Bernal and Guzmán (2006a); copyright by APS

The head-on case was revisited in Bernal and Guzmán (2006a) with a 2D axisymmetric code. In particular, these evolutions show that the final state will depend on the total energy of the system (e.g. the sum of kinetic, gravitational and self-interaction energies). If the total energy is positive, the stars exhibit solitonic behavior both for identical stars (see Fig. 16) and non-identical stars. When the total energy is negative, the gravitational force is the main driver of the dynamics of the system. This case produces a true collision, forming a single object with large perturbations, which slowly decays by gravitational cooling, as displayed in Fig. 17.

The first simulations of boson stars with full general relativity were reported in Balakrishna (1999), where the gravitational waves were computed for a head-on collision. The general behavior is similar to the one displayed for the Newtonian limit; the stars attract each other through gravitational interaction and then merge to produce a largely perturbed boson star. However, in this case the merger of the binary was promptly followed by collapse to a black hole, an outcome not possible when working within Newtonian gravity instead of general relativity. Unfortunately, very little detail was given on the dynamics.

Much more elucidating was work in axisymmetry (Lai 2004), in which head-on collisions of identical boson stars were studied in the context of critical collapse (discussed in Sect. 6.1) with general relativity. Stars with identical masses of $M = 0.47 \approx 0.75\,M_{\max }$ were chosen, and so it is not surprising that for small initial momenta the stars merged together to form an unstable single star (i.e., its mass was larger than the maximum allowed mass, $M_{\max }$). The unstable *hypermassive* star subsequently collapsed to a black hole. However, for large initial momentum the stars passed through each other, displaying a form of solitonic behavior since the individual identities were recovered after the interaction. The stars showed a particular interference pattern during the overlap, much like that displayed in Figs. 1 and 16.

Another study considered the very high speed, head-on collision of BSs (Choptuik and Pretorius 2010). Beginning with two identical boson stars boosted with Lorentz factors ranging as high as 4, the stars generally demonstrate solitonic behavior upon collision, as shown in the insets of Fig. 25. This work is further discussed in Sect. 6.2.

The interaction of non-identical boson stars was studied in Palenzuela et al. (2007) using a 3D Cartesian code to simulate head-on collisions of stars initially at rest. It was found that, for a given separation, the merger of two stars would produce an unstable star that collapses to a black hole if the initial individual mass were $M \ge 0.26 \approx 0.4\,M_{\max }$. For smaller masses, the resulting star would avoid gravitational collapse and its features would strongly depend on the initial configuration. The parameterization of the initial data was written as a superposition of the single boson-star solution $\phi _0(\mathbf {r})$, located at different positions $\mathbf {r_1}$ and $\mathbf {r_2}$

90 $$\begin{aligned} \phi = {}^{(1)}\phi _0(\mathbf {r_1}) e^{i \omega t} + {}^{(2)}\phi _0(\mathbf {r_2}) e^{i (\epsilon \omega t + \theta )}. \end{aligned}$$

Many different initial configurations are possible with this parameterization. The precise solution $\phi _0$ is unaffected by changing the direction of rotation (within the complex plane) via $\epsilon =\pm \, 1$ or by a phase shift $\theta$.

**Fig. 18:**
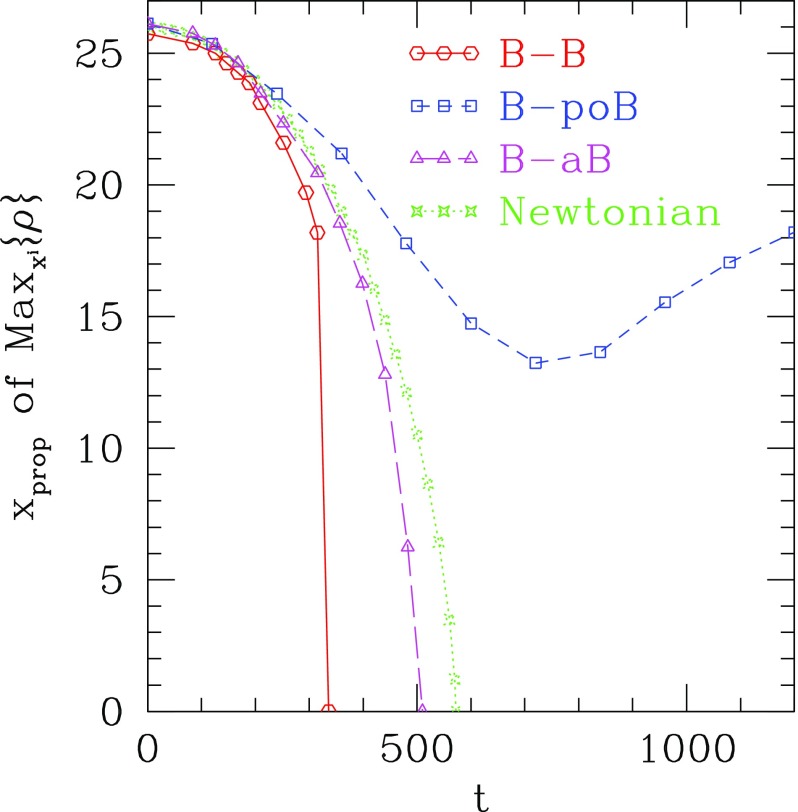
The position of the center of one BS in a head-on binary as a function of time for (i) [B-B] identical BSs, (ii) [B-poB] opposite phase pair, and (iii) [B-aB] a boson–anti-boson pair. A simple argument is made which qualitatively matches these numerical results, as discussed in Sect. 4.2. Also shown is the expected trajectory from a simple Newtonian two-body estimate. Reprinted with permission from Palenzuela et al. (2007); copyright by APS

When $\epsilon =-1\,$, the Noether charge changes sign and the compact object is then known as an *anti-boson star*. Three particular binary cases were studied in detail: (i) identical boson stars ($\epsilon =1$, $\theta =0$), (ii) the pair in phase opposition ($\epsilon =1$, $\theta =\pi$), and (iii) a boson–anti-boson pair ($\epsilon =-\,1$, $\theta =0$). The trajectories of the centers of the stars are displayed in Fig. 18, together with a simple estimate of the expected trajectory assuming Newtonian gravity. The figure makes clear that the merger depends strongly on the kind of pair considered, that is, on the interaction between the scalar fields.

A simple energy argument is made in Palenzuela et al. (2007) to understand the differing behavior. In the weak gravity limit when the stars are well separated, one can consider the local energy density between the two stars. In addition to the contribution due to each star separately, a remaining term $\varDelta$ results from the interaction of the two stars and it is precisely this term that will depend on the parameters $\epsilon$ and $\theta$. This term takes the simple form

91 $$\begin{aligned} \varDelta = \varDelta _0\, \cos [ (1-\epsilon ) \omega \, t - \theta ], \end{aligned}$$

where $\varDelta _0$ is a positive definite quantity. One then observes that the identical pair will have an increased energy density $\varDelta =+\,\varDelta _0$ resulting in a deeper (and more attractive) gravitational well between the stars. In contrast, the pair with opposite phases has a decreased energy density $\varDelta =-\,\varDelta _0$ between them, resulting in a gravitational well less attractive than the area surrounding it. This less attractive well results in an effective repulsion relative to the identical pairing. The boson–anti-boson pair has an interaction that is harmonic in time $\varDelta =\varDelta _0 \cos \left( 2\omega t\right)$ and therefore sometimes positive and sometimes negative. However, if the time scale of interaction is not particularly fast, then the interaction averages to zero. Note that the boson–anti-boson pair trajectory is the closest to the simple Newtonian estimate. The qualitative behavior agrees very well with the numerical results.

The orbital case was later studied in Palenzuela et al. (2008). This case is much more involved both from the computational point of view (i.e., there is less symmetry in the problem) and from the theoretical point of view, since for the final object to settle into a stationary, rotating boson star it must satisfy the additional quantization condition for the angular momentum of Eq. (71).

One simulation consisted of an identical pair each with individual mass $M=0.5$, with small orbital angular momentum such that $J \le N$. In this case, the binary merges forming a rotating bar that oscillates for some time before ultimately splitting apart. This can be considered as a scattered interaction, which could not settle down to a stable boson star unless all the angular momentum was radiated.

**Fig. 19:**
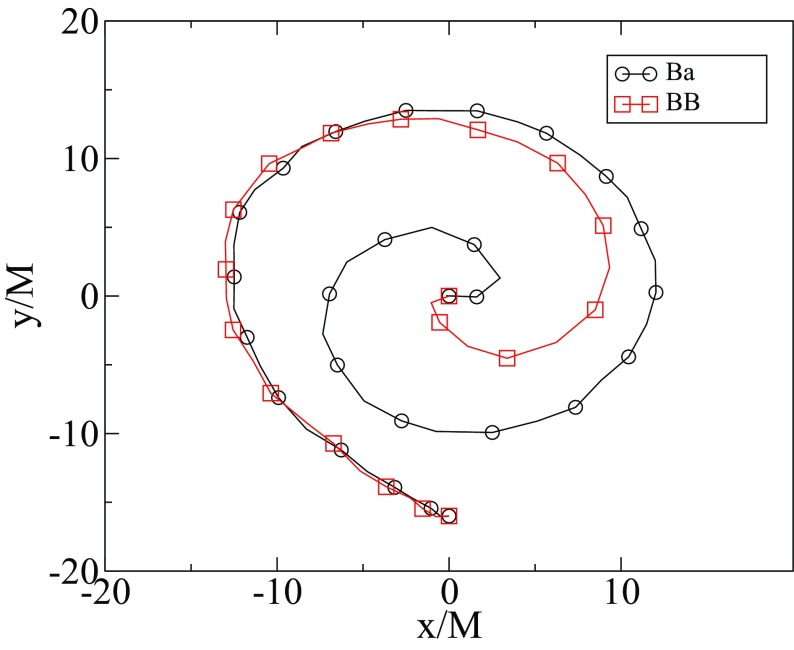
The position of the center of one BS within an orbiting binary as a function of time for the two cases: (i) [B-B] identical BSs and (ii) [B-poB] opposite phase pair. Notice that the orbits are essentially identical at early times (and large separations), but that they start to deviate from each other on closer approach. This is consistent with the internal structure of each member of the binary being irrelevant at large separations. Reprinted with permission from Palenzuela et al. (2008); copyright by APS

In the case of boson–anti-boson pair, the total Noether charge is already trivial, and the final object resembles the structure of a rotating dipole. The pair in opposition of phase was not considered because of the repulsive effect from the interaction. The cases with very small angular momentum $J \ll N$ or with $J \le N$ collapsed to a black hole soon after the merger. The trajectories for this latter case are displayed in Fig. 19, indicating that the internal structure of the star is irrelevant (as per the effacement theorem Damour 1987) until the scalar fields overlap.

Other simulations of orbiting, identical binaries have been performed within the conformally flat approximation instead of full GR, which neglects gravitational waves (Mundim 2010). Three different qualitative behaviors were found. For high angular momentum, the stars orbit for comparatively long times around each other. For intermediate values, the stars merged and formed a pulsating and rotating boson star. For low angular momentum, the merger produces a black hole. No evidence was found of the stars splitting apart after the merger.

Three dimensional simulations of solitonic core mergers colliding two or more boson stars in the Newtonian limit (Schrödinger–Poisson) are studied in the context of dark matter with different mass ratios, phases and orbital angular momentum (Schwabe et al. 2016). The final core mass does not depend strongly on the phase difference nor on the angular momentum. Cotner (2016) also studies collisions within the Schrödinger–Poisson system and discusses implications for dark matter. However, this work focuses on the head-on case and includes effects of different mass ratios, relative phases, self-couplings, and separation distances. Interestingly, analytic estimates are compared to the numerical simulations (Cotner 2016).

The dynamics of particularly compact boson stars are interesting to contrast with the dynamics of black holes because, at least in part, we now have observations of the gravitational waves from binary BH mergers (discussed more in Sect. 5.3). To this end, the study of the head-on collision of solitonic boson stars (which can be quite compact Cardoso et al. 2016) found the dynamics to be qualitatively similar to those observed previously with mini-boson stars (Palenzuela et al. 2007). However, the gravitational waves emitted displayed significant differences and, in some cases, closely resembled the signal from a binary black hole merger.

**Fig. 20:**
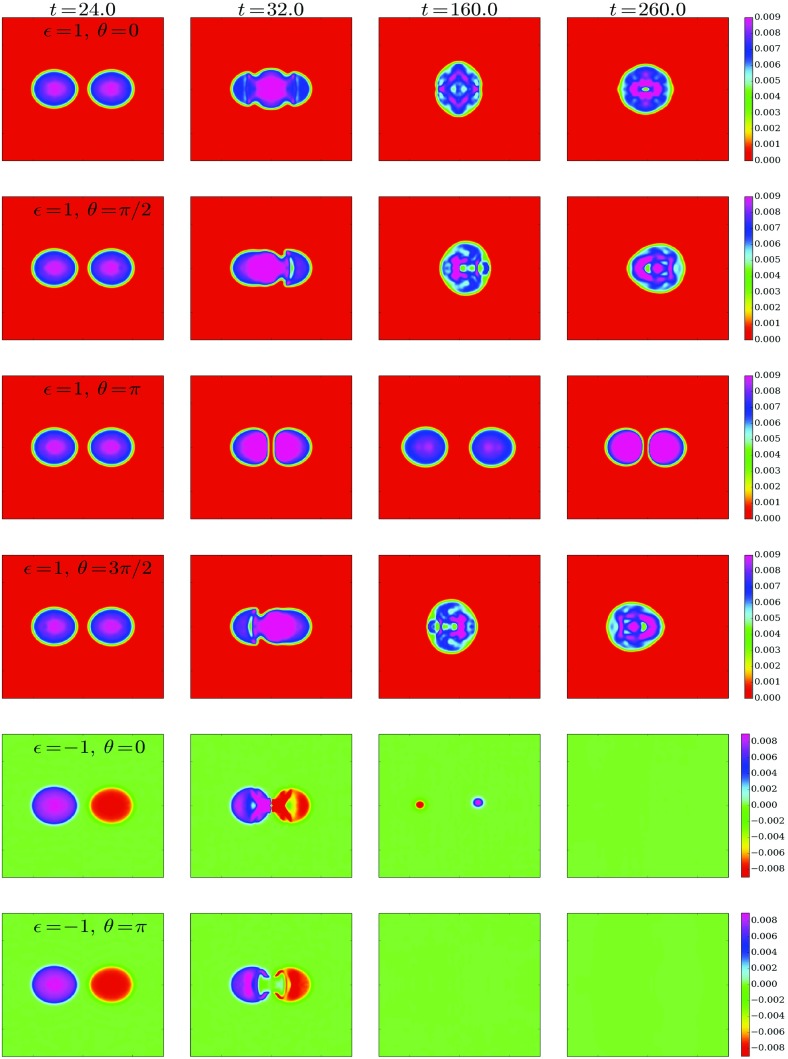
Snapshots in time of the Noether charge density in the $z=0$ plane for head-on binary collisions of compact solitonic boson stars. Each row corresponds to a different boson-boson and boson-anti-boson case studied with a phase shift $\theta$ as described by Eq. (90). The collision of the stars occurs approximately at $t=28$. The result of the boson–boson merger is a single boson star except in the case with $\theta =\pi$. The stars in the boson–anti-boson case annihilate each other during the merger. Reprinted with permission from Bezares et al. (2017); copyright by APS

**Fig. 21:**
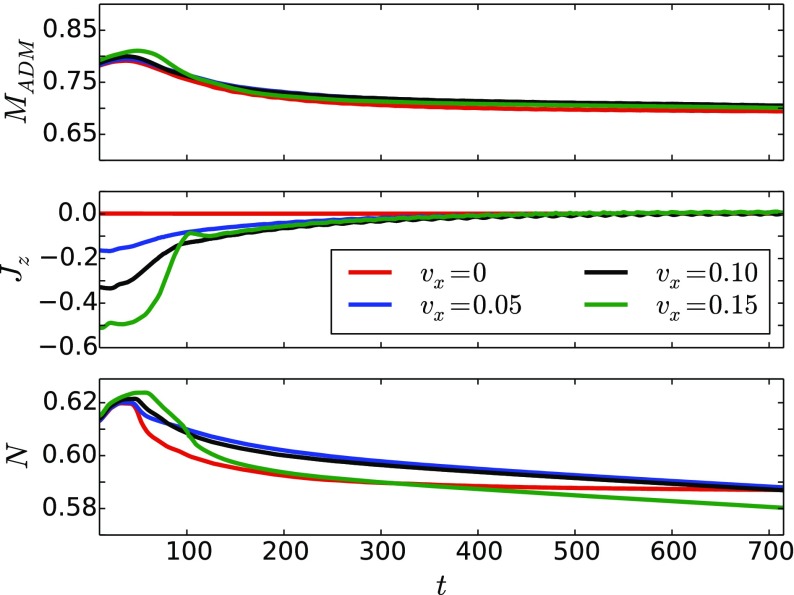
ADM mass (top panel), angular momentum $J_{z}$ (middle panel), and Noether charge (bottom panel) as functions of time for the orbital binary collisions of compact solitonic boson stars with different tangential boost velocities. During the coalescence, approximately 5% of the mass and Noether charge is radiated, as well as most of the angular momentum. Reprinted with permission from Bezares et al. (2017); copyright by APS

These studies have been extended to the orbital case in Bezares et al. (2017). Surprisingly, for stars not so massive as to collapse promptly, the merger does not lead to a rotating boson star but instead to a non-rotating perturbed BS (snapshots of some of these simulations are shown in Fig. 20). As apparent in Fig. 21, the system radiates most of its angular momentum via scalar radiation and gravitational waves soon after the merger.

## Boson stars in astronomy

Scalar fields are often employed by astronomers and cosmologists in their efforts to model the Universe. Most models of inflation adopt a scalar field as the *inflaton* field, the vacuum energy of which drives the exponential inflation of the Universe. Dark energy also motivates many scalar field models, such as *k-essence* and *phantom* energy models. It is therefore not surprising that boson stars, as compact configurations of scalar field, are called upon to provide consequences similar to those observed.

### As astrophysical stellar objects

We have already discussed a number of similarities between boson stars and models of neutron stars. Just as one can parameterize models of neutron stars by their central densities, one can consider a 1-parameter family of boson stars according to the central magnitude of the scalar field. The mass is then a function of this parameter, and one finds the existence of a local maximum across which solutions transition from stable to unstable, just as is the case for neutron stars. Similarly, models of neutron stars can be constructed with different equations of state, whereas boson stars are constructed with differing scalar field potentials.

One difference of consequence concerns the stellar surface. Neutron stars of course have a surface at which the fluid density is discontinuous, as discussed for example in Gundlach and Leveque (2011), Gundlach and Please (2009). In contrast, the scalar field that constitutes the boson star is smooth everywhere and lacks a particular surface. In its place, one generally defines a radius that encompasses some percentage (e.g. 99%) of the stellar mass. Such a difference could have observational consequences when matter accretes onto either type of star.

It is still an open question whether some of the stars already observed and interpreted as neutron stars could instead be astrophysical boson stars. In a similar fashion, it is not known whether many, if not all, of the stars we observe already have a bosonic component that has settled into the gravitational well of the star (see Sect. 3.6 for a discussion of fermion–boson stars). The bosonic contribution may arise from exotic matter, which could appear at high densities inside the neutron star or from some sort of dark matter accretion (Güver et al. 2014). This possibility has gained popularity over the last years and there have been several attempts to constrain the properties of weakly interacting dark matter particles (WIMPs) by examining signatures related to their accretion and/or annihilation inside stars (for instance, see Kouvaris and Tinyakov 2010 and works cited in the introduction).

Recently, it was suggested that, due to the stronger gravitational field of neutron stars compared to other stars such as white dwarfs and main sequence stars, WIMPs will accrete more efficiently, leading to two different possibilities. If the dark matter is its own antiparticle, it will self-annihilate and heat the neutron star. This temperature increase could be observable in old stars, especially if they are close to the galactic center (Kouvaris and Tinyakov 2010; de Lavallaz and Fairbairn 2010). If WIMPs do not self-annihilate, they will settle in the center of the star forming a fermion–boson star (as discussed in Sect. 3.6). The accretion of dark matter would then increase the star’s compactness until the star collapses (de Lavallaz and Fairbairn 2010) (see discussion of BSs as a source of dark matter in Sect. 5.4). Núñez et al. (2011) follows such work by considering the result of a collision between a BH and a boson star. In particular, they consider the problem as a perturbation of a black hole via scalar accretion and analyze the resulting gravitational-wave output.

Because of the similarities between boson stars and neutron stars, one finds that boson stars are often used in place of the other. This is especially so within numerical work because boson stars are easier to evolve than neutron star models. One can, for example compare the gravitational-wave signature of a boson-star merger with that of more conventional compact object binaries consisting of BHs and/or NSs. Differentiating BSs from other compact objects with gravitational-wave observations is discussed further in Sect. 5.3.

With the continued advancement in observation, both in the electromagnetic and gravitational spectra, perhaps soon we will have evidence for these questions. At the same time, further study of boson stars can help identify possible distinguishing observational effects in these bands. One example where knowledge is lacking is the interaction between boson stars with a magnetic field. Whereas a neutron star can source its own magnetic field and a neutral star can obtain an induced charge when moving with respect to a magnetic field, we are aware of no studies of the interaction of boson stars with a magnetic field.

### Compact alternatives to black holes

As a localized scalar field configuration, a boson star can be constructed as a non-interacting compact object, as long as one does not include any explicit coupling to any electromagnetic or other fields. In that respect, it resembles a BH, although it lacks a horizon. Can observations of purported BHs be fully explained by massive boson stars? See Psaltis (2008) for a review of such observations.

Neutron stars also lack horizons, but, in contrast to a boson star, have a hard surface. A hard surface is important because one would expect accretion onto such a surface to have observable consequences. Can a boson star avoid such consequences? Yuan et al. (2004) consider the the viability of $10\,M_{\odot }$ boson stars as BH candidates in X-ray binaries. They find that accreting gas collects not at the surface (which the star lacks), but instead at the center, which ultimately should lead to Type I X-ray bursts. Because these bursts are not observed, the case against boson stars as black hole mimickers is weakened (at least for BH candidates in X-ray binaries).

Guzmán and Rueda-Becerril (2009) considers a simplified model of accretion and searches for boson-star configurations that would mimic an accreting black hole. Although they find matches, they argue that light deflection about a boson star will differ from the BH they mimic because of the lack of a photon sphere. Further work studies the scalar field tails about boson stars and compares them to those of BHs (Lora-Clavijo et al. 2010). If indeed a boson star collapses to a BH, then one could hope to observe the QNM of the massive scalar field, as described in Hod (2011). Differences between accretion structures surrounding boson stars and black holes are analyzed in Meliani et al. (2015), showing that the accretion tori around boson stars have different characteristics than in the vicinity of a black hole. Further studies on the subject include disk (Meliani et al. 2016) and supersonic winds (Gracia-Linares and Guzman 2016) accreting onto boson stars.

Some of the strongest evidence for the existence of BHs is found at the center of most galaxies. Observational evidence strongly suggests supermassive objects (of the order of millions of solar mass) occupying a small region (of order an astronomical unit), which is easily explained by a supermassive BH (Boehle et al. 2012). While definitive evidence for a BH horizon from conventional electromagnetic telescopes is perhaps just on the “horizon” (Johannsen et al. 2016; Broderick et al. 2011), there are those who argue for the viability of supermassive boson stars at galactic centers (Torres et al. 2000). There could potentially be differences in the (electromagnetic) spectrum between a black hole and a boson star, but there is considerable freedom in adjusting the boson star potential to tweak the expected spectrum (Guzmán 2007). However, there are stringent constraints on BH alternatives to Sgr A* by the low luminosity in the near infrared (Broderick and Narayan 2006). In particular, the low luminosity implies a bound on the accretion rate assuming a hard surface radiating thermally and, therefore, the observational evidence favors a black hole because it lacks such a surface. In particular, although a BS lacks a surface, any material it accretes would accumulate and that material would have a surface that would radiate thermally.

At least two possible ways to test the nature of astrophysical black hole candidates are apparent; either with X-ray observations or with millimeter very long baseline interferometry (VLBI).

The analysis of X-ray reflection spectroscopy with data provided by the current X-ray missions can only provide weak constraints on boson stars (Cao et al. 2016), Proca stars (Shen et al. 2017), and hairy Kerr BHs (Ni et al. 2016). The quasi-periodic oscillations (QPOs) observed in the X-ray flux emitted by accreting compact objects also provide a powerful tool both to constrain deviations from Kerr and to search for exotic compact objects. Therefore, a future eXTP mission or LOFT-like mission could set very stringent constraints on black holes with bosonic hair and on (scalar or Proca) boson stars (Franchini et al. 2017).

VLBI, on the other hand, may be able to resolve Sgr A*, the closest supermassive black hole located at the center of our galaxy. The Event Horizon Telescope (EHT) promises to resolve angular scales of the order of the horizon scale, and so soon there will be accurate images of the closest surroundings of the supermassive compact object at the center of the Galaxy. These images will allow the study of so-called *BH shadows*, that is, the gravitational lensing and redshift effect due to the BH on the radiation from background sources.

**Fig. 22:**
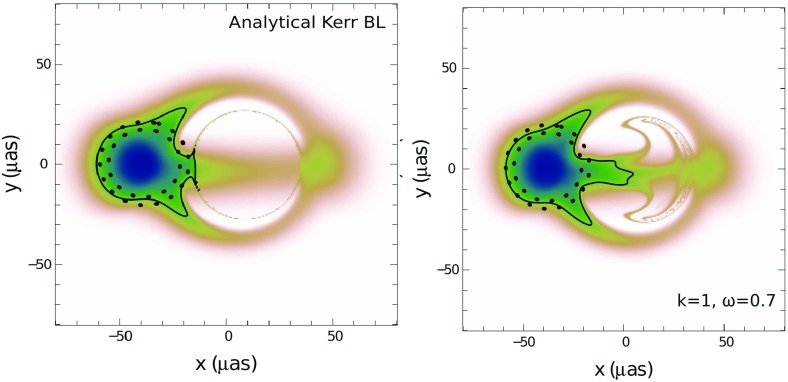
Computed images as might be expected from the EHT for: (left) a Kerr black hole and (right) a fast spinning boson star with accretion according to certain assumptions. The similarity in images indicates that ruling out a BS candidate in images of Sgr A* may prove difficult. Reprinted with permission from Vincent et al. (2016); copyright by IOP

Images of an accretion torus around Sgr A*, assuming this compact object is a boson star, are computed in Vincent et al. (2016). However, their results demonstrate that very relativistic rotating boson stars produce images extremely similar to Kerr black holes, making them difficult to distinguish from a black hole. Figure 22 displays images predicted from this work for both a BH and a BS which appear quite similar. The conclusion of Vincent et al. (2016) expresses a number of interesting caveats, and this study is also discussed as part of a more wide ranging paper about efforts to firmly establish Sgr A* as a BH (Eckart et al. 2017).

It has also been shown in Cunha et al. (2015) that hairy Kerr BHs can exhibit very distinct shadows from those of their vacuum counterparts when the light source is sufficiently far away from the BH. These differences remain, albeit less dramatically, when the BH is surrounded by an emitting torus of matter (Vincent et al. 2016).

Other studies have also studied the difference in appearance of a BS with that of the presumed BH in the center of our galaxy. Bin-Nun (2013) argues that, because BSs have an extended mass distribution that is transparent to electromagnetic radiation, the resulting strong gravitational lensing images of the S stars in the galactic center would yield much brighter images than a BH of similar mass. Horvat et al. (2013) studies BSs with a nonminimally coupled scalar field and makes a similar argument about bright images.

One can also consider differences between the motion of celestial bodies about BSs versus BHs. In particular, finding general geodesic motion of test particles in the space–time of boson stars generally requires numerical integration. Geodesics around non-compact boson star were studied in Diemer et al. (2013), finding additional bound orbits of massive test particles close to the center of the star that are not present in the Schwarzschild case and that could be used to make predictions about extreme-mass-ratio inspirals (EMRIs), such as the stars orbiting Sagittarius A*. One can also compute the mass parameters of compact objects from redshifts and blueshifts emitted by geodesic particles around them (Becerril et al. 2016). The motion of charged, massive test particles in the spacetime of charged boson stars was considered in Brihaye et al. (2014).

There are other possible BH mimickers, and a popular recent one is the *gravastar* (Mazur and Mottola 2001). Common among all these alternatives is the lack of an event horizon. Both gravastars and BSs undergo an ergoregion instability for high spin $\hbox {J}/(\hbox {GM}^2) >0.4$ (Cardoso et al. 2008). As mentioned above for BSs, gravitational waves may similarly be able to distinguish gravastars from BHs (Pani et al. 2009). In order to reach the high compactnesses needed to mimic a BH, one can adopt specialized potentials (Cardoso et al. 2016), but an alternative is to embed the BS within a global monopole as studied in Reid and Choptuik (2016) and Marunović and Murković (2014).

### As source of gravitational waves

The era of gravitational-wave (GW) astronomy began in 2015, precisely 100 years after Einstein’s development of GR. In particular, LIGO directly detected the gravitational waves from the inspiral, merger, and ringdown of a BH binary (Abbott 2016b). This observation has since been followed by a second BH binary and many more observations are expected from the facility (Abbott 2016a). The excitement about these first direct detections should also help ensure the completion of other gravitational wave observatories such as LISA (Armano 2017), KAGRA (Flaminio 2016), and next generation detectors (Abbott 2017).

Now that we have actual GW observations in hand, it behooves us to extract as much science as possible from this new window on the Universe. Much work has already appeared examining the implications of these initial detections (Yunes et al. 2016; Yagi and Stein 2016; Abbott 2016c). In this paper, of course, we are concerned with the implications for BSs: (i) could these extent observations actually represent the signal from a pair of boson stars instead of BHs? (ii) might we observe a signal from boson stars, and, if so, what templates will we need? or (iii) can we place tight bounds excluding the existence of boson stars?

A BS binary system is the most natural GW source. However, at early times, the precise structure of the stars is irrelevant and the signatures are largely the same whether the binary is composed of NSs, BHs, or BSs. However, during the late inspiral and merger, internal structure becomes important. In particular for boson stars, the relative phase determines the GW signature (Palenzuela et al. 2007, 2008; Cardoso et al. 2016).

Gravitational waves may be an ideal messenger for revealing dark matter (discussed in Sect. 5.4). If new dark sector particles can form exotic compact objects (ECOs) of astronomical size, then the first evidence for such objects—and their underlying microphysical description—may arise in gravitational-wave observations. The relationship between the macroscopic properties of ECOs, such as their GW signatures, with their microscopic properties, and hence new particles, was studied in Giudice et al. (2016). The GW efficiency of compact binaries generally is examined in Hanna et al. (2017).

**Fig. 23:**
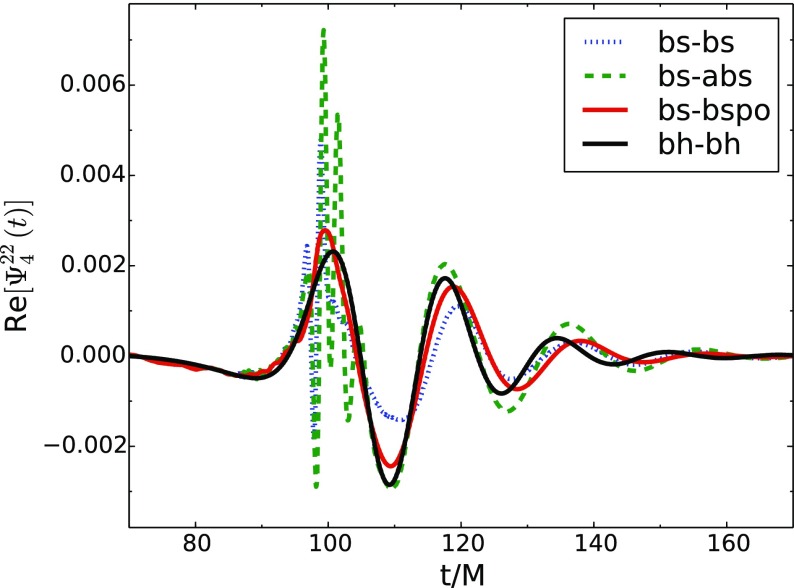
Gravitational waves, represented by the $l=m=2$ mode of the Newman-Penrose scalar, $\varPsi _4$, emitted during the head-on collision of two solitonic BSs. For all configurations, the final object is massive enough to promptly collapse to a BH. However, for the boson–boson and boson–anti-boson configurations the late inspiral signatures differ significantly from the corresponding binary black-hole signal. Reprinted with permission from Cardoso et al. (2016); copyright by the authors

Along the same lines, the tidal Love numbers for different ECOs, including different families of boson stars, are calculated in Cardoso et al. (2017). The tidal Love number, which encodes the deformability of a self-gravitating object within an external tidal field, depends significantly both on the object’s internal structure and on the dynamics of the gravitational field. Present and future gravitational-wave detectors can potentially measure this quantity in a binary inspiral of compact objects and impose constraints on boson stars. Direct numerical simulations in head-on collision already have shown similarities in the gravitational waves emitted by black holes and boson stars in some cases (Cardoso et al. 2016). Fig. 23 compares the expected GW signal of a BH binary with various BS binaries.

One can also examine supermassive BHs and ask whether they could instead be some form of BS. In particular, the observation of gravitational waves from such objects may be able to distinguish BHs from BSs (Berti and Cardoso 2006). Such a test would occur in the bandwidth for a space-based observatory such as the LISA mission (Danzmann 2017). Because BSs allow for orbits within what would otherwise be a black hole event horizon, geodesics will exhibit extreme pericenter precession resulting in potentially distinguishable gravitational radiation (Kesden et al. 2005). In any case, observations of supermassive objects at the centers of galaxies can be used to constrain the scalar field parameters of possible mimickers (Barranco and Bernal 2011a). In Macedo et al. (2013a), the authors construct mini-boson, boson and solitonic boson stars and analyze the gravitational and scalar response of boson star spacetimes to an inspiralling stellar-mass object.

### As origin of dark matter

Studies of stellar orbits within various galaxies produce *rotation curves*, which indicate galactic mass within the radius of the particular orbit. The discovery that these curves remain flat at large radius suggests the existence of a large *halo* of massive, yet dark, matter that holds the galaxy together despite its large rotation (see Feng 2010 for a review). However, what precise form of matter could fulfill the observational constraints is still very much unclear. Scalar fields are an often used tool in the cosmologist’s toolkit, but one cannot have a regular, static configuration of scalar field to serve as the halo (Pena and Sudarsky 1997) (see Dias et al. 2011 as discussed in Sect. 6.3 for a discussion of *rotating* boson stars with embedded, rotating BH solutions). Instead, some form of boson star represents a possible candidate for providing the necessary dark mass.

Compact binaries are the primary target of LIGO, but instead of neutron stars or black holes, Soni and Zhang (2017) studies the expected signal from binaries consisting of *SU*(*N*) glueball objects, one of the simplest models of dark matter. More discussion of the merger of two BSs and the production of GW can be found in Sect. 4.2. At the lower frequencies targeted by LISA, if galaxies generally possess some extended, supermassive configuration, then the inspiral of small compact body into this field will result in both dynamical friction and dark matter accretion, in addition to radiation-reaction (Macedo et al. 2013b). These dynamical effects may potentially be encoded on observable gravitational waves from the inspiral.

Boson stars can be matched onto the observational constraints for galactic dark matter halos (Lee 2010a; Sharma et al. 2008). However, multi-state boson stars that superpose various boson-star solutions (e.g., an unexcited solution with an excited solution) can perhaps find better fits to the constraints (Ureña-López and Bernal 2010). Boson stars at the galactic scale may not exhibit general relativistic effects and can be effectively considered as Bose–Einstein condensates (BEC) with angular momentum (Rindler-Daller and Shapiro 2012).

Representing dark matter as BSs also offers certain computational benefits, avoiding some of the costs of modeling the particles themselves with an *N*-body scheme. For example, Davidson and Schwetz (2016) studies structure formation of an axion dark matter model with ground state solutions of the appropriate Schrödinger-Poisson system along with quantum pressure term (see Eq. 58). Even if dark matter consists of clumps of weakly interacting massive particles (WIMPs) instead of BSs, Mendes and Yang (Mendes and Yang 2016) map clumps of such particles to perturbed boson stars and study their tidal deformability, bypassing the large computational cost of studying the dynamics of these WIMPs with an *N*-body code. Tidal deformability of BSs was also studied recently in the context of testing strong-field general relativity (Cardoso et al. 2017).

Instead of galactic scale BSs, one could instead argue for the accumulation of bosonic field in neutron stars. Such solutions contain the “normal” fermionic matter as well as a bosonic component (discussed above in Sect. 3.6). However, the accumulation of additional mass in a neutron star, already the expected last stage before complete collapse to black hole, might conceivably lead to the star’s collapse. If indeed collapse can be expected, then the existence of old neutron stars would place constraints on such a form of dark matter (Yz et al. 2012; Jamison 2013; Bramante et al. 2013). In the face of such arguments, Kouvaris and Tinyakov (2013), Bell et al. (2013) instead argue that a broad range of realistic models survive such constraints. Most recently, Brito et al. (2015) argue with perturbation and numerical methods that old stars are in fact stable to the accretion of light bosons by an efficient gravitational cooling mechanism (see also the Ph.D. thesis by Brito 2016).

Another dark matter model arising from a scalar field is *wave dark matter* (Bray and Goetz 2014; Bray and Parry 2013; Goetz 2015a, b). In particular, they examine Tully–Fisher relationships predicted by this wave dark matter model (Bray and Goetz 2014; Goetz 2015b). High resolution simulations of a non-relativstic Bose–Einstein condensate within this model reproduce the large scale structure of standard cold dark matter while differing inside galaxies (Schive et al. 2014).

Other studies solve the Gross–Pitaevskii equation for a Bose–Einstein condensate as a model of dark matter stars and study its stability properties (Li et al. 2012; Madarassy and Toth 2015; Marsh and Pop 2015).

The solitonic nature of boson stars (see Fig. 1) lends itself naturally to the wonderful observation of dark matter in the Bullet Cluster (Lee et al. 2008). Lee and Lim (2010b) attempts to determine a minimum galactic mass from such a match.

Interestingly, Barranco et al. (2011) foregoes boson stars and instead looks for quasi-stationary scalar solutions about a Schwarzschild black hole that could conceivably survive for cosmological times. Another approach is to use scalar fields for both the dark matter halo and the supermassive, central object. Amaro-Seoane et al. (2010) looks for such a match, but finds no suitable solutions. Quite a number of more exotic models viably fit within current constraints, including those using Q-balls (Doddato 2012).

Section 4.2 discusses the dynamics of boson stars including some references commenting on the implications of the dynamics for dark matter.

## Boson stars in mathematical relativity

Although the experimental foundation for the existence of boson stars is completely lacking, on the theoretical and mathematical front, boson stars are well studied. Recent work includes a mathematical approach in terms of large and small data (Frank and Lenzmann 2009a), followed up by studying singularity formation (Lenzmann and Lewin 2011) and uniqueness (Frank and Lenzmann 2009b; Lenzmann 2009) for a certain boson star equation. In Cho et al. (2009), they study radial solutions of the semi-relativistic Hartree type equations in terms of global well-posedness. Bičák et al. (2010) demonstrates stationarity of time periodic scalar field solutions.

Already discussed in Sect. 3.9 has been the *no hair conjecture* in the context of BSs holding a central BH within. Beyond just existence, however, boson stars are often employed mathematically to study dynamics. Here, we concentrate on a few of these topics that have attracted recent interest.

### Black hole critical behavior

If one considers some initial distribution of energy and watches it evolve, generally one arrives at one of three states. If the energy is sufficiently weak in terms of its gravity, the energy might end up *dispersing* to larger and large distances. However, if the energy is instead quite large, then perhaps it will concentrate until a *black hole* is formed. Or, if the form of the energy supports it, some of the energy will condense into a *stationary state*.

In his seminal work (Choptuik 1993), Choptuik considers a real, massless scalar field and numerically evolves various initial configurations, finding either dispersion or black-hole formation. By parameterizing these initial configurations, say by the amplitude of an initial pulse *p*, and by tuning this parameter, he was able to study the *threshold for black-hole formation* at which he found fascinating black-hole critical behavior. In particular, his numerical work suggested that continued tuning could produce as small a black hole as one wished. This behavior is analogous to a phase transition in which the black-hole mass serves as an order parameter. Similar to phase transitions, one can categorize two types of transition that distinguish between whether the black-hole mass varies continuously (Type II) or discontinuously (Type I). For Choptuik’s work with a massless field, the transition is therefore of Type II because the black-hole mass varies from zero continuously to infinitesimal values.

Subsequent work has since established that this critical behavior can be considered as occurring in the neighborhood of a *separatrix* between the *basins of attraction* of the two end states. For $p=p^*$, the system is precisely critical and remains on the (unstable) separatrix. Similarly other models find such threshold behavior occurring between a stationary state and black-hole formation. Critical behavior about stationary solutions necessarily involve black-hole formation “turning-on” at finite mass, and is therefore categorized as Type I critical behavior.

The critical surface, therefore, appears as a *co-dimension 1* surface, which evolutions increasingly approach as one tunes the parameter *p*. The distance from criticality $|p-p^*|$ serves as a measure of the extent to which a particular initial configuration has excited the unstable mode that drives solutions away from this surface. For Type II critical behavior, the mass of the resulting black-hole mass scales as a power law in this distance, whereas for Type I critical behavior, it is the survival time of the critical solution that scales as a power law. See Gundlach et al. (2007) for a recent review.

We have seen that boson stars represent stationary solutions of Einstein’s equations and, thus, one would correctly guess that they may occur within Type I black-hole critical behavior. To look for such behavior, Hawley and Choptuik (2000) begin their evolutions with boson-star solutions and then perturb them both dynamically and gravitationally. They, therefore, included in their evolutionary system a distinct, free, massless, real scalar field which couples to the boson star purely through its gravity.

**Fig. 24:**
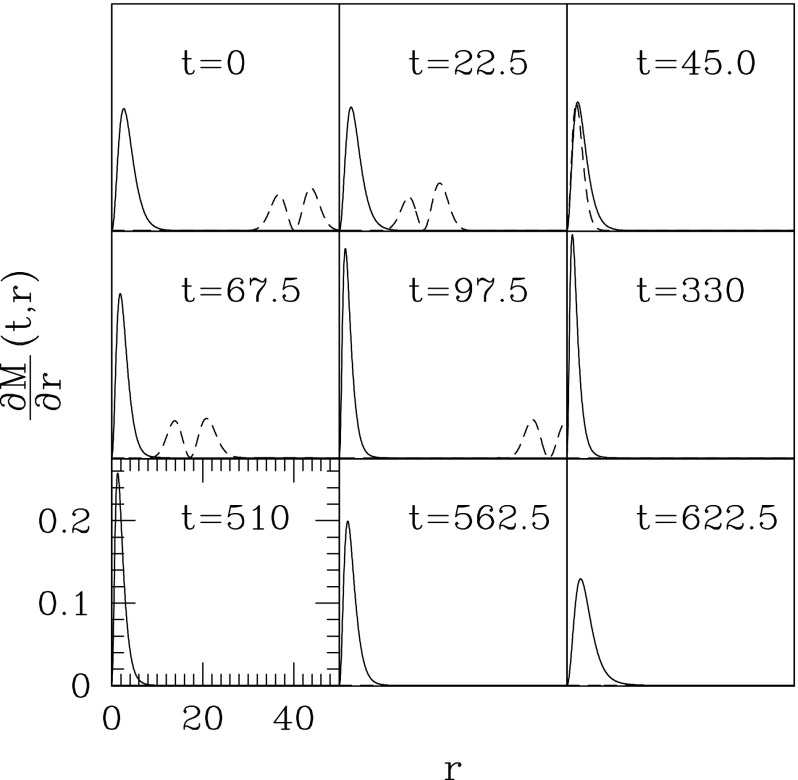
Evolution of a boson star (solid line) perturbed by a shell of scalar field (dashed line). Shown is the mass density $\partial M/\partial r$ for each contribution. By $t \approx 100$ the real scalar field pulse has departed the central region and perturbed the boson star into an unstable, compact configuration. Contrast the $t=0$ frame with that of $t=97.5$ and note the increase in compaction. This unstable BS survives until $t\approx 500$ only because the initial perturbation has been tuned to one part in $10^{15}$ and indicates Type I critical behavior. Reprinted with permission from Lai and Choptuik (2007)

The initial data, therefore, consisted of a boson star surrounded by a distant, surrounding shell of real scalar field parametrized by the amplitude of the shell. For small perturbations, the boson star oscillated about an unstable boson star before settling into a low mass, stable solution (see Fig. 24). For large perturbations, the real scalar field serves to compress the initial star and, after a period of oscillation about an unstable boson star, the complex field collapses to a black hole. By tuning the initial perturbation, they find a longer and longer lived unstable boson star, which serves as the critical solution (see Fig. 14). The survival time $\tau$ obeys a power law in terms of the distance from criticality $|p-p^*|$

92 $$\begin{aligned} \tau \propto \gamma \ln |p - p^*|, \end{aligned}$$

where $\gamma$ is a real constant that depends on the characteristic instability rate of the particular unstable boson star approached in the critical regime.

One can also consider these BSs in axisymmetry in which non-spherically symmetric modes could potentially become important. A first step in this direction studied spherically symmetric BSs within conformally flat gravity (which does not allow for gravitational waves) in axisymmetry (Rousseau 2003). Later, better resolution using adaptive mesh refinement within full general relativity was achieved by Lai (2004), Lai and Choptuik (2007), which upheld the results found within spherical symmetry. This work thus suggests that there are either no additional, unstable, axisymmetric modes or that such unstable modes are extremely slowly growing.

A very different type of critical behavior was also investigated by Lai (2004). By boosting identical boson stars toward each other and adjusting their initial momenta, he was able to tune to the threshold for black-hole formation. At the threshold, he found that the time till black-hole formation scaled consistent with Type I critical behavior and conjectured that the critical solution was itself an unstable boson star. This is one of the few fully nonlinear critical searches in less symmetry than spherical symmetry, and the first of Type I behavior in less symmetry. A related study colliding neutron stars instead of boson stars similarly finds Type I critical behavior (Jin and Suen 2007) and subsequently confirmed by Kellermann et al. (2010).

The gauged stars discussed in Sect. 3.11 also serve as critical solutions in spherical symmetry (Choptuik et al. 1996, 1999; Millward and Hirschmann 2003).

### Hoop conjecture

An interesting use of boson stars was made by Choptuik and Pretorius (2010). They sought to answer classically whether the ultra-relativistic collision of two particles results in black-hole formation. Such a question clearly has relevance to hopes of producing black holes at the LHC (see, for example, Landsberg 2006; Park 2012; Sirunyan 2017). Guidance on this question is provided by Thorne’s *Hoop Conjecture* (Thorne 1972) which suggests that, if one squeezes energy into some spherical space of dimension less than the Schwarzschild radius for that energy, then a black hole is formed.

**Fig. 25:**
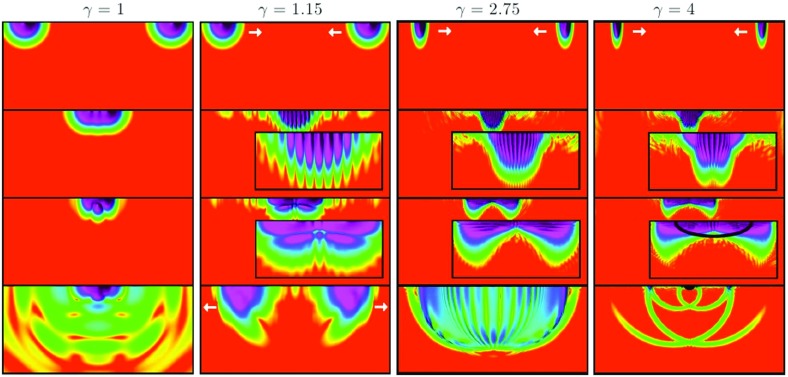
Evolutions of the head-on collisions of identical boson stars boosted toward each other with initial Lorentz factors $\gamma$ as indicated. Time flows downward within each column and the top edge displays the axis of symmetry. The color-scale indicates the value of $|\phi |$. In the middle frames one sees the interference pattern characteristic of high kinetic energy BS collisions (as mentioned in Fig. 1). In the last column on the right, the collision produces a BH with apparent horizon indicated by the black oval in the third frame. Reprinted with permission from Choptuik and Pretorius (2010); copyright by APS

They, therefore, numerically collide boson stars head-on at relativistic energies to study black-hole formation from just such dynamical “squeezing”. Here, the nature of boson stars is largely irrelevant as they serve as simple bundles of energy that can be accelerated (see Fig. 25). However, unlike using boosted black-hole solutions, the choice of boson stars avoids any type of bias or predisposition to formation of a black hole. In addition, a number of previous studies of boson star head-on collisions showed interesting interference effects at energies below the threshold for black-hole formation (Choi et al. 2009; Choi 2002; Lai 2004; Mundim 2010). Indeed, it has been proposed that such an interference pattern could be evidence for the bosonic nature of dark matter because of evidence that an ideal fluid fails to produce such a pattern (González and Guzmán 2011).

Choptuik and Pretorius (2010) find that indeed black-hole formation occurs at energies *below* that estimated by the Hoop Conjecture. This result is only a classical result consistent with the conjecture, but if it had not held, then there would have been no reason to expect a quantum theory to be consistent with it.

### Other dimensions and anti-de Sitter spacetime

Much work has been invested recently in considering physics in other dimensions. Motivation comes from various ideas including string theory (more dimensions) such as the AdS/CFT correspondence and holography (one fewer dimensions) (Maldacena 1998; McGreevy 2010; Polchinski 2010). Another source of motivation comes from the fact that higher dimensional black holes can have different properties than those in three spatial dimensions (Emparan and Reall 2008). Perhaps BSs will similarly display novel properties in other dimensions.

In lower dimensional AdS ($2+1$) spacetimes, early work in 1998 studied exact solutions of boson stars (Sakamoto and Shiraishi 1998a; Degura et al. 2001; Sakamoto and Shiraishi 1998). Higher dimensional scenarios were apparently first considered qualitatively a few years later in the context of brane world models (Stojkovic 2003). This discussion was followed with a detailed analysis of the 3, 4, and 5 dimensional AdS solutions (Astefanesei and Radu 2003).

More recently, Fodor et al. (2010b) considers oscillatons in higher dimensions and measures the scalar mass loss rate for dimensions 3, 4, and 5. They extend this work considering inflationary spacetimes (Fodor et al. 2010a). Brihaye et al. (2014) and Herdeiro et al. (2015a) construct higher dimensional black hole solutions (Myers–Perry BHs) with scalar hair, and, in so doing, they find higher dimensional, rotating BS solutions.

The axisymmetric rotating BSs discussed in Sect. 3.5 satisfy a coupled set of nonlinear, elliptic PDEs in two dimensions. One might therefore suspect that adding other dimensions will only make things more difficult. As it turns out, however, moving to four spatial dimensions provides for another angular momentum, *independent* of the one along the *z*-direction (for example). Each of these angular momenta are associated with their own orthogonal plane of rotation. And so if one chooses solutions with equal magnitudes for each of these momenta, the solutions depend on only a *single* radial coordinate. This choice results in the remarkable simplification that one need only solve ODEs to find rotating solutions (Kunz et al. 2006).

In Hartmann et al. (2010), they extend this idea by assuming an ansatz for two complex scalar fields with equal magnitudes of angular momentum in the two independent directions. Letting the complex doublet be denoted by $\varPhi$, the ansatz takes the form

93 $$\begin{aligned} \varPhi = \phi (r) e^{i\omega t} \left( \begin{array}{c} \sin \theta e^{i\varphi _1} \\ \cos \theta e^{i\varphi _2} \end{array} \right) \end{aligned}$$

in terms of the two angular coordinates $\varphi _1$ and $\varphi _2$. One observes that the BS (i) retains a profile $\phi (r)$, (ii) possesses harmonic time dependence, and (iii) maintains single-valuedness in the two angles (the ansatz assumes a rotational quantum number of one). They find solutions that are both globally regular and asymptotically flat but these solutions appear only stable with weak gravitational coupling (Hartmann et al. 2010). Solutions have since been constructed in AdS${}_3$ (Stotyn and Mann 2012; Stotyn et al. 2014a), in higher odd-dimensional AdS spacetimes (Stotyn et al. 2014b), and in Gauss–Bonnet gravity (Henderson et al. 2015) (see Sect. 3.10 for BS in alternative theories of gravity).

The work of Dias et al. (2011) makes ingenious use of this 5D ansatz to construct rotating black holes with only a single Killing vector. They set the potential of Hartmann et al. (2010) to zero so that the scalar fields are massless and they add a (negative) cosmological constant to work in anti-de Sitter (AdS). Some of their solutions represent a black hole embedded inside a rotating BS. They find solutions for rotating black holes in 5D AdS that correspond to a *bar mode* for rotating neutron stars in 3D (see also Shibata and Yoshino 2010 for a numerical evolution of a black hole in higher dimensions which demonstrates such bar formation; see Emparan and Reall 2008 for a review of black holes in higher dimensions).

One might expect such a non-symmetric black hole to settle into a more symmetric state via the emission of gravitational waves. However, AdS provides for an essentially reflecting boundary in which the black hole can be in equilibrium. The distortion of the higher dimensional black hole also has a correspondence with the discrete values of the angular momentum of the corresponding boson star. For higher values of the rotational quantum number, the black hole develops multiple “lobes” about its center. Very compact BSs constructed with this single Killing vector posses an ergoregion (Brihaye et al. 2015).

This construction can be extended to arbitrary odd-dimensional AdS spacetimes (Stotyn et al. 2012). Finding the solutions perturbatively, they explicitly show that these solutions approach (i) the boson star and (ii) the Myers–Perry black-hole solutions in AdS (Myers and Perry 1986) in different limits. Boson stars, along with neutron stars and black holes, in five dimensions are discussed in Brihaye and Delsate (2016), and see Emparan and Reall (2008) for a review of black holes in higher dimensions.

In AdS${}_4$ this ansatz cannot be used, and the construction of spinning boson stars requires the solution of the appropriate multidimensional PDEs as is done in Radu and Subagyo (2012).

Interest in the dynamics of AdS spacetimes increased significantly with the work of Bizoń and Rostworowski (2011) who studied the collapse of a scalar field in spherically symmetric, global AdS${}_4$. They argued that a non-zero initial amplitude for the scalar field would generically result in gravitational collapse to black hole via turbulent instability. In particular, fully nonlinear numerical evolutions of small amplitude configurations of scalar field generically resulted in a continued sharpening of the initial pulse as it reflected off the AdS boundary. This instability in the bulk is considered the mechanism that achieves thermal equilibration in the conformal theory on the boundary.

Many studies followed trying to answer the many questions arising from this work. Did this instability extend to any initial amplitude? Was the instability tied to the precise structure of AdS or instead simply to the fact that the spacetime was bounded?

One question in particular concerned the implications of this instability for localized solutions which might naturally be expected to extend their stability in asymptotically flat spacetimes. To that end, Buchel et al. (2013) studied boson stars in AdS, and found that indeed they are stable. In the course of understanding how the boson stars were stable, this work found a whole class of initial data that appear immune to the instability. Later work added to this class, namely breather solutions in AdS (Fodor et al. 2015). Linear perturbation analysis of spherically symmetric Proca stars in AdS suggests that these too will be stable (Duarte and Brito 2016b).

The same authors of (Dias et al. 2011) also report on the existence of geons in $3+1$ AdS “which can be viewed as gravitational analogs of boson stars” (Dias et al. 2012) (recall that boson stars themselves arose from Wheeler’s desire to construct local electrovacuum solutions). These bundles of gravitational energy are stable to first order due to the confining boundary condition adopted with AdS. The instability of these geons, black holes, and boson stars were studied in Dias et al. (2011) in the context of the turbulent instability reported in Bizoń and Rostworowski (2011), but later these authors argued for their nonlinear stability (Dias et al. 2012).

Basu et al. (2010) also studies black-hole solutions in 5D AdS. They find solutions for black holes with scalar hair that resemble a boson star with a BH in its center. The stability of charged boson stars with a massive scalar field in five-dimensional AdS was studied in Brihaye et al. (2013). Also in AdS${}_5$, Buchel studies boson stars in a type IIB supergravity approximation to string theory in which the *U*(1) symmetry of the complex field is gauged instead of global (Buchel 2015; Buchel and Buchel 2015). A range of solutions, including Q-balls and shell solutions, for different values of the cosmological constant have similarly been constructed (Hartmann et al. 2013; Hartmann and Riedel 2012, 2013).

Basu et al. (2010) also studies black hole solutions in 5D AdS. They find solutions for black holes with scalar hair that resemble a boson star with a BH in its center.

Earlier work with BSs in lower dimensional AdS was reported in Astefanesei and Radu (2003).

Boson stars in AdS with charge are constructed in Hu et al. (2012), and they are also used as the background for a study of *entanglement entropy* (Nogueira 2013) (for a review of holographic entanglement entropy see Rangamani and Takayanagi 2017). Charged boson stars with spin in AdS have also been studied (Kichakova et al. 2014). See Gentle et al. (2012) for a review of charged scalar solitons in AdS.

### Analog gravity and physical systems

The study of the correspondence between gravitating systems and analogous physical systems goes by the name of *analog gravity* (Barceló et al. 2011). One example of such an analog is the *acoustic* or *dumb* hole, analogous to a black hole, that requires information to flow in a particular direction. For such a system the analog of Hawking radiation is expected, and, remarkably, such radiation may have already been measured (Unruh 2014).

Analogs exist for BS as well. Recent work of Roger et al. (2016) finds an interesting optical analog of Newtonian BSs. So far this analog appears to be mostly associated with corresponding equations of motion as opposed to some deep physical correspondence that might reveal critical insight.

A more concrete analog is the formation of a Bose–Einstein condensation such as studied in Kühnel and Rampf (2014) in the context of superradiance (see Sect. 3.9). However, note that as mentioned in Sect. 1.1, ground state BSs can be considered as condensed states of bosons without invoking any analogy (Chavanis 2015; Chavanis and Matos 2017).

## Final remarks

Boson stars have a long history as candidates for all manner of phenomena, from fundamental particle, to galactic dark matter. A huge variety of solutions have been found and their dynamics studied. Mathematically, BS are fascinating soliton-like solutions. Astrophysically, they represent possible explanations of black hole candidates and dark matter, with observations constraining BS properties.

Remarkably, in just the last 5 years since the first version of this review, two incredibly significant experimental results have appeared, and a third may soon be on its way. The Higgs particle has been found by the LHC, the first scalar particle. While its instability makes it less than promising as the fundamental constituent of boson stars, perhaps its discovery heralds a new period of scalar discoveries.

Far from the quantum particle regime of the LHC, LIGO has directly detected gravitational waves completely consistent with the merger of a binary black-hole system as predicted by general relativity. Not only does this put an end to the nagging questions about whether LIGO can really detect such extremely weak signals, but, as said often in the wake of these detections, it opens a new window into some of the most energetic events in the Universe. Although it is impossible to predict what new phenomena will be observed, one can hope that gravitational waves will further illuminate the nature of compact objects.

In the electromagnetic spectrum, the EHT just completed its 10 day observation, with an image expected by early next year (2018). The images from EHT are anxiously awaited because of their potential for demonstrating explicitly the presence of a horizon in Sgr A*. Of course, nature often surprises and so perhaps these images may instead lend credence to BSs.

With all of this experimental and observational data, physicists need to provide unambiguous tests and explicit predictions. Much work on that front is ongoing, trying to tease out observational differences from alternative models of gravity or alternatives to the standard compact objects (BHs and NSs) (Berti et al. 2015, 2016; Choptuik et al. 2015). Black holes were once exotic and disbelieved, but now BHs are the commonly accepted standard while BSs are proposed as just one of many exotic compact objects.

Perhaps future work on boson stars will be experimental, if fundamental scalar fields are observed, or if evidence arises indicating the boson stars uniquely fit galactic dark matter. But regardless of any experimental results found by these remarkable experiments, there will always be regimes unexplored by experiments where boson stars will find a natural home.

## Footnotes

1
http://www.maplesoft.com.

## References

[CR1] Aad G (2012). Observation of a new particle in the search for the standard model Higgs boson with the ATLAS detector at the LHC. Phys Lett B.

[CR2] Abbott BP (2016). GW151226: observation of gravitational waves from a 22-solar-mass binary black hole coalescence. Phys Rev Lett.

[CR3] Abbott BP (2016). Observation of gravitational waves from a binary black hole merger. Phys Rev Lett.

[CR4] Abbott BP (2016). Tests of general relativity with GW150914. Phys Rev Lett.

[CR5] Abbott BP (2017). Exploring the sensitivity of next generation gravitational wave detectors. Class Quantum Gravity.

[CR6] Adam C, Grandi N, Klimas P, Sánchez-Guillén J, Wereszczyński A (2010). Compact boson stars in k field theories. Gen Relativ Gravity.

[CR7] Agnihotri P, Schaffner-Bielich J, Mishustin IN (2009). Boson stars with repulsive self-interactions. Phys Rev D.

[CR8] Akhoury R, Gauthier CS (2008) Galactic halos and black holes in non-canonical scalar field theories. ArXiv e-prints arXiv:0804.3437

[CR9] Alcubierre M (2008). Introduction to $$3+1$$ numerical relativity, International Series of Monographs on Physics.

[CR10] Alcubierre M, Becerril R, Guzmán FS, Matos T, Núñez D, Ureña-López LA (2003). Numerical studies of $$\phi ^{2}$$-oscillatons. Class Quantum Gravity.

[CR11] Alcubierre M, Degollado JC, Núñez D, Ruiz M, Salgado M (2010). Dynamic transition to spontaneous scalarization in boson stars. Phys Rev D.

[CR12] Alic D (2009) Theoretical issues in numerical relativity simulations. PhD thesis, Universitat de les Illes Balears, Palma. http://hdl.handle.net/10803/9438

[CR13] Amaro-Seoane P, Barranco J, Bernal A (2010). Constraining scalar fields with stellar kinematics and collisional dark matter. J Cosmol Astropart Phys.

[CR14] Antusch S, Orani S (2016). Impact of other scalar fields on oscillons after hilltop inflation. J Cosmol Astropart Phys.

[CR15] Antusch S, Cefala F, Orani S (2017). Gravitational waves from oscillons after inflation. Phys Rev Lett.

[CR16] Armano M (2017). Charge-induced force-noise on free-falling test masses: results from LISA pathfinder. Phys Rev Lett.

[CR17] Arnowitt R, Deser S, Misner CW, Witten L (1962). The dynamics of general relativity. Gravitation: an introduction to current research.

[CR18] Arodź H, Karkowski J, Świerczyński Z (2009). Spinning Q-balls in the complex signum-Gordon model. Phys Rev D.

[CR19] Astefanesei D, Radu E (2003). Boson stars with negative cosmological constant. Nucl Phys B.

[CR20] Baibhav V, Maity D (2017). Boson stars in higher-derivative gravity. Phys Rev D.

[CR21] Balakrishna J (1999) A numerical study of boson stars: Einstein equations with a matter source. PhD thesis, Washington University, St. Louis. arXiv:gr-qc/9906110

[CR22] Balakrishna J, Seidel E, Suen WM (1998). Dynamical evolution of boson stars. II. Excited states and self-interacting fields. Phys Rev D.

[CR23] Balakrishna J, Bondarescu R, Daues G, Guzmán FS, Seidel E (2006). Evolution of 3d boson stars with waveform extraction. Class Quantum Gravity.

[CR24] Balakrishna J, Bondarescu R, Daues G, Bondarescu M (2008). Numerical simulations of oscillating soliton stars: excited states in spherical symmetry and ground state evolutions in 3d. Phys Rev D.

[CR25] Bao W, Dong X (2011). Numerical methods for computing ground states and dynamics of nonlinear relativistic hartree equation for boson stars. J Comput Phys.

[CR26] Barceló C, Liberati S, Visser M (2011) Analogue gravity. Living Rev Relativ 14:lrr-2011-3. 10.12942/lrr-2011-3. http://www.livingreviews.org/lrr-2011-3. arXiv:gr-qc/0505065 10.12942/lrr-2011-3 PMC5255896 28179830

[CR27] Barranco J, Bernal A (2011a) Constraining scalar field properties with boson stars as black hole mimickers. In: Ureña-López LA, Morales-Técotl HA, Linares-Romero R, Santos-Rodríguez E, Estrada-Jiménez S (eds) VIII workshop of the gravitation and mathematical physics division of the Mexican Physical Society, American Institute of Physics, Melville, NY, AIP conference proceedings, vol 1396, pp 171–175. 10.1063/1.3647542. arXiv:1108.1208

[CR28] Barranco J, Bernal A (2011). Self-gravitating system made of axions. Phys Rev D.

[CR29] Barranco J, Bernal A, Degollado JC, Diez-Tejedor A, Megevand M, Alcubierre M, Núñez D, Sarbach O (2011). Are black holes a serious threat to scalar field dark matter models?. Phys Rev D.

[CR30] Bartnik R, McKinnon J (1988). Particlelike solutions of the Einstein–Yang–Mills equations. Phys Rev Lett.

[CR31] Basu P, Bhattacharya J, Bhattacharyya S, Loganayagam R, Minwalla S, Umesh V (2010). Small hairy black holes in global AdS spacetime. J High Energy Phys.

[CR32] Battye RA, Sutcliffe PM (2000). Q-ball dynamics. Nucl Phys B.

[CR33] Baumgarte TW, Shapiro SL (1999). Numerical integration of Einstein’s field equations. Phys Rev D.

[CR34] Baumgarte TW, Shapiro SL (2010). Numerical relativity: solving Einstein’s equations on the computer.

[CR35] Becerril R, Valdez-Alvarado S, Nucamendi U (2016). Obtaining mass parameters of compact objects from redshifts and blueshifts emitted by geodesic particles around them. Phys Rev D.

[CR36] Bell NF, Melatos A, Petraki K (2013). Realistic neutron star constraints on bosonic asymmetric dark matter. Phys Rev D.

[CR37] Bernal A, Guzmán FS (2006). Scalar field dark matter: head-on interaction between two structures. Phys Rev D.

[CR38] Bernal A, Guzmán FS (2006). Scalar field dark matter: nonspherical collapse and late-time behavior. Phys Rev D.

[CR39] Bernal A, Barranco J, Alic D, Palenzuela C (2010). Multistate boson stars. Phys Rev D.

[CR40] Berti E, Cardoso V (2006). Supermassive black holes or boson stars? Hair counting with gravitational wave detectors. Int J Mod Phys D.

[CR41] Berti E, Cardoso V, Crispino LCB, Gualtieri L, Herdeiro C, Sperhake U (2016) Numerical relativity and high energy physics: recent developments. Int J Mod Phys D 25:1641022. 10.1142/S0218271816410224, proceedings, 3rd Amazonian Symposium on Physics and 5th NRHEP Network Meeting is approaching: Celebrating 100 Years of General Relativity: Belem, Brazil. arXiv:1603:06146

[CR42] Berti E (2015). Testing general relativity with present and future astrophysical observations. Class Quantum Gravity.

[CR43] Bezares M, Palenzuela C, Bona C (2017). Final fate of compact boson star mergers. Phys Rev D.

[CR44] Bhatt JR, Sreekanth V (2009) Boson stars: chemical potential and quark condensates. ArXiv e-prints arXiv:0910.1972

[CR45] Bičák J, Scholtz M, Tod P (2010) On asymptotically flat solutions of Einstein’s equations periodic in time II. Spacetimes with scalar-field sources. Class Quantum Gravity 27:175011. 10.1088/0264-9381/27/17/175011. arXiv:1008.0248

[CR46] Bin-Nun AY (2013) Method for detecting a boson star at Sgr A* through gravitational lensing. ArXiv e-prints arXiv:1301.1396

[CR47] Bizoń P, Rostworowski A (2011). On weakly turbulent instability of anti-de Sitter space. Phys Rev Lett.

[CR48] Boehle A, Ghez A, Schoedel R, Yelda S, Meyer L (2012) New orbital analysis of stars at the Galactic center using speckle holography. In: AAS 219th meeting, American Astronomical Society, Washington, DC, Bull. Am. Astron. Soc., vol 44

[CR49] Bogolyubskiĭ IL, Makhan’kov VG (1977). Dynamics of spherically symmetrical pulsons of large amplitude. JETP Lett.

[CR50] Bona C, Palenzuela-Luque C, Bona-Casas C (2009). Elements of numerical relativity and relativistic hydrodynamics: from Einstein’s equations to astrophysical simulations. Lecture Notes in Physics.

[CR51] Brady PR, Choptuik MW, Gundlach C, Neilsen DW (2002). Black-hole threshold solutions in stiff fluid collapse. Class Quantum Gravity.

[CR52] Bramante J, Fukushima K, Kumar J (2013). Constraints on bosonic dark matter from observation of old neutron stars. Phys Rev D.

[CR53] Bray HL, Goetz AS (2014) Wave dark matter and the Tully–Fisher relation. ArXiv e-prints arXiv:1409.7347

[CR54] Bray HL, Parry AR (2013) Modeling wave dark matter in dwarf spheroidal galaxies. ArXiv e-prints arXiv:1301.0255

[CR55] Brihaye Y, Delsate T (2016) Boson stars, neutron stars and black holes in five dimensions. ArXiv e-prints arXiv:1607.07488

[CR56] Brihaye Y, Hartmann B (2009). Angularly excited and interacting boson stars and $$q$$ balls. Phys Rev D.

[CR57] Brihaye Y, Hartmann B (2016) Minimal boson stars in 5 dimensions: classical instability and existence of ergoregions. Class Quantum Gravity 33:065002. 10.1088/0264-9381/33/6/065002. arXiv:1509.04534

[CR58] Brihaye Y, Riedel J (2014). Rotating boson stars in five-dimensional Einstein–Gauss–Bonnet gravity. Phys Rev D.

[CR59] Brihaye Y, Verbin Y (2009). Spherical structures in conformal gravity and its scalar–tensor extension. Phys Rev D.

[CR60] Brihaye Y, Verbin Y (2010). Spherical non-Abelian solutions in conformal gravity. Phys Rev D.

[CR61] Brihaye Y, Hartmann B, Radu E (2005). Boson stars in SU(2) Yang–Mills-scalar field theories. Phys Lett B.

[CR62] Brihaye Y, Caebergs T, Delsate T (2009a) Charged-spinning-gravitating Q-balls. ArXiv e-prints arXiv:0907.0913

[CR63] Brihaye Y, Caebergs T, Hartmann B, Minkov M (2009). Symmetry breaking in (gravitating) scalar field models describing interacting boson stars and Q-balls. Phys Rev D.

[CR64] Brihaye Y, Hartmann B, Tojiev S (2013). Stability of charged solitons and formation of boson stars in five-dimensional anti-de Sitter spacetime. Class Quantum Gravity.

[CR65] Brihaye Y, Diemer V, Hartmann B (2014). Charged Q-balls and boson stars and dynamics of charged test particles. Phys Rev D.

[CR66] Brihaye Y, Herdeiro C, Radu E (2014). Myers–Perry black holes with scalar hair and a mass gap. Phys Lett B.

[CR67] Brihaye Y, Hartmann B, Riedel J (2015). Self-interacting boson stars with a single Killing vector field in anti-de Sitter space-time. Phys Rev D.

[CR68] Brihaye Y, Cisterna A, Erices C (2016). Boson stars in biscalar extensions of Horndeski gravity. Phys Rev D.

[CR69] Brito R (2016) Fundamental fields around compact objects: massive spin-2 fields, superradiant instabilities and stars with dark matter cores. PhD thesis, Lisboa University. arXiv:1607.05146

[CR70] Brito R, Cardoso V, Okawa H (2015). Accretion of dark matter by stars. Phys Rev Lett.

[CR71] Brito R, Cardoso V, Pani P (2015). Superradiance, Lecture Notes in Physics.

[CR72] Brito R, Cardoso V, Herdeiro CAR, Radu E (2016). Proca stars: gravitating Bose–Einstein condensates of massive spin 1 particles. Phys Lett B.

[CR73] Brito R, Cardoso V, Macedo CFB, Okawa H, Palenzuela C (2016). Interaction between bosonic dark matter and stars. Phys Rev D.

[CR74] Broderick AE, Narayan R (2006). On the nature of the compact dark mass at the Galactic center. Astrophys J Lett.

[CR75] Broderick AE, Loeb A, Reid MJ (2011). Localizing Sagittarius A* and M87 on microarcsecond scales with millimeter very long baseline interferometry. Astrophys J.

[CR76] Buchel A (2015) AdS boson stars in string theory. ArXiv e-prints arXiv:1510.08415

[CR77] Buchel A, Buchel M (2015) On stability of nonthermal states in strongly coupled gauge theories. ArXiv e-prints arXiv:1509.00774

[CR78] Buchel A, Liebling SL, Lehner L (2013). Boson stars in AdS spacetime. Phys Rev D.

[CR79] Burikham P, Harko T, Lake MJ (2016). Mass bounds for compact spherically symmetric objects in generalized gravity theories. Phys Rev D.

[CR80] Cao Z, Cardenas-Avendano A, Zhou M, Bambi C, Herdeiro CAR, Radu E (2016). Iron k$$\alpha $$ line of boson stars. J Cosmol Astropart Phys.

[CR81] Cardoso V, Gualtieri L (2016). Testing the black hole ‘no-hair’ hypothesis. Class Quantum Gravity.

[CR82] Cardoso V, Pani P, Cadoni M, Cavaglià M (2008). Ergoregion instability of ultracompact astrophysical objects. Phys Rev D.

[CR83] Cardoso V, Hopper S, Macedo CFB, Palenzuela C, Pani P (2016). Gravitational-wave signatures of exotic compact objects and of quantum corrections at the horizon scale. Phys Rev D.

[CR84] Cardoso V, Franzin E, Maselli A, Pani P, Raposo G (2017). Testing strong-field gravity with tidal Love numbers. Phys Rev D.

[CR85] Chatrchyan S (2012). Observation of a new boson at a mass of 125 GeV with the CMS experiment at the LHC. Phys Lett B.

[CR86] Chavanis PH (2011). Mass-radius relation of Newtonian self-gravitating Bose–Einstein condensates with short-range interactions. I. Analytical results. Phys Rev D.

[CR87] Chavanis PH (2012). Growth of perturbations in an expanding universe with Bose–Einstein condensate dark matter. Astron Astrophys.

[CR88] Chavanis PH, Calmet X (2015). Self-gravitating Bose–Einstein condensates. Quantum aspects of black holes, Fundamental Theories of Physics.

[CR89] Chavanis PH, Harko T (2012). Bose–Einstein condensate general relativistic stars. Phys Rev D.

[CR90] Chavanis PH, Matos T (2017). Covariant theory of Bose-Einstein condensates in curved spacetimes with electromagnetic interactions: the hydrodynamic approach. Eur Phys J Plus.

[CR91] Cho Y, Ozawa T, Sasaki H, Shim Y (2009). Remarks on the semirelativistic Hartree equations. Discrete Contin Dyn Syst A.

[CR92] Chodosh O, Shlapentokh-Rothman Y (2015) Time-periodic Einstein–Klein–Gordon bifurcations of Kerr. ArXiv e-prints arXiv:1510.08025

[CR93] Choi D, Lai CW, Choptuik MW, Hirschmann EW, Liebling SL, Pretorius F (2009) Dynamics of axisymmetric (head-on) boson star collisions. http://laplace.physics.ubc.ca/Group/Papers/choi-etal-prd-05/choi-etal-prd-05.pdf, unpublished

[CR94] Choi DI (1998) Numerical studies of nonlinear Schrödinger and Klein–Gordon systems: techniques and applications. PhD thesis, The University of Texas, Austin. http://laplace.physics.ubc.ca/Members/matt/Doc/Theses/

[CR95] Choi DI (2002). Collision of gravitationally bound Bose–Einstein condensates. Phys Rev A.

[CR96] Choptuik MW (1993). Universality and scaling in gravitational collapse of a massless scalar field. Phys Rev Lett.

[CR97] Choptuik MW, Pretorius F (2010). Ultrarelativistic particle collisions. Phys Rev Lett.

[CR98] Choptuik MW, Chmaj T, Bizoń P (1996). Critical behavior in gravitational collapse of a Yang–Mills field. Phys Rev Lett.

[CR99] Choptuik MW, Hirschmann EW, Marsa RL (1999). New critical behavior in Einstein–Yang–Mills collapse. Phys Rev D.

[CR100] Choptuik MW, Lehner L, Pretorius F, Ashtekar A, Berger BK, Isenberg J, MacCallum M (2015). Probing strong-field gravity through numerical simulations. General relativity and gravitation: a centennial perspective.

[CR101] Chruściel PT, Costa JL, Heusler M (2012) Stationary black holes: uniqueness and beyond. Living Rev Relativ 15:lrr-2012-7. 10.12942/lrr-2012-7, http://www.livingreviews.org/lrr-2012-7. arXiv:1205.6112 10.12942/lrr-2012-7 PMC5255892 28179837

[CR102] Coleman SR (1985). Q-balls. Nucl Phys B.

[CR103] Colpi M, Shapiro SL, Wasserman I (1986). Boson stars: gravitational equilibria of self-interacting scalar fields. Phys Rev Lett.

[CR104] Contaldi CR, Wiseman T, Withers B (2008). TeVeS gets caught on caustics. Phys Rev D.

[CR105] Cook GB (2000) Initial data for numerical relativity. Living Rev Relativ 3:lrr-2000-5. 10.12942/lrr-2000-5, http://www.livingreviews.org/lrr-2000-5, arXiv:gr-qc/0007085 10.12942/lrr-2000-5 PMC5660886 29142501

[CR106] Cook GB, Shapiro SL, Teukolsky SA (1994). Rapidly rotating neutron stars in general relativity: realistic equations of state. Astrophys J.

[CR107] Cotner E (2016). Collisional interactions between self-interacting nonrelativistic boson stars: effective potential analysis and numerical simulations. Phys Rev D.

[CR108] Cunha PVP, Herdeiro CAR, Radu E, Runarsson HF (2015). Shadows of Kerr black holes with scalar hair. Phys Rev Lett.

[CR109] Dafermos M, Rodnianski I, Shlapentokh-Rothman Y (2014) A scattering theory for the wave equation on Kerr black hole exteriors. ArXiv e-prints arXiv:1412.8379

[CR110] Damour T, Hawking SW, Israel W (1987). The problem of motion in Newtonian and Einsteinian gravity. Three hundred years of gravitation.

[CR111] Damour T, Esposito-Farèse G (1996). Tensor–scalar gravity and binary-pulsar experiments. Phys Rev D.

[CR112] Danzmann Kea (2017) LISA: laser interferometer space antenna. a proposal in response to the ESA call for L3 mission concepts. In: Technical report, Max Planck Institute for Gravitational Physics (Albert Einstein Institute), Potsdam. https://www.elisascience.org/files/publications/LISA_L3_20170120.pdf

[CR113] Dariescu C, Dariescu MA (2010). Boson nebulae charge. Chin Phys Lett.

[CR114] Davidson S, Schwetz T (2016). Rotating drops of axion dark matter. Phys Rev D.

[CR115] Degura Y, Sakamoto K, Shiraishi K (2001). Black holes with scalar hair in ($$2+1$$)-dimensions. Gravit Cosmol.

[CR116] de Lavallaz A, Fairbairn M (2010). Neutron stars as dark matter probes. Phys Rev D.

[CR117] Delgado JFM, Herdeiro CAR, Radu E, Runarsson H (2016). Kerr-Newman black holes with scalar hair. Phys Lett B.

[CR118] de Sousa CMG, Tomazelli JL, Silveira V (1998). Model for stars of interacting bosons and fermions. Phys Rev D.

[CR119] de Sousa CMG, Silveira V, Fang LZ (2001). Slowly rotating boson–fermion star. Int J Mod Phys D.

[CR120] Derrick GH (1964). Comments on nonlinear wave equations as models for elementary particles. J Math Phys.

[CR121] Dias ÓJC, Masachs R (2017). Hairy black holes and the endpoint of AdS$$_4$$ charged superradiance. J High Energy Phys.

[CR122] Dias ÓJC, Horowitz GT, Santos JE (2011). Black holes with only one Killing field. J High Energy Phys.

[CR123] Dias ÓJC, Horowitz GT, Marolf D, Santos JE (2012). On the nonlinear stability of asymptotically anti-de Sitter solutions. Class Quantum Gravity.

[CR124] Dias ÓJC, Horowitz GT, Santos JE (2012). Gravitational turbulent instability of anti-de Sitter space. Class Quantum Gravity.

[CR125] Dias ÓJC, Santos JE, Way B (2016). Numerical methods for finding stationary gravitational solutions. Class Quantum Gravity.

[CR126] Diemer V, Eilers K, Hartmann B, Schaffer I, Toma C (2013). Geodesic motion in the space-time of a noncompact boson star. Phys Rev D.

[CR127] Diez-Tejedor A, Gonzalez-Morales AX (2013). No-go theorem for static scalar field dark matter halos with no noether charges. Phys Rev D.

[CR128] Doddato F, McDonald J (2012). New Q-ball solutions in gauge-mediation, Affleck–Dine baryogenesis and gravitino dark matter. J Cosmol Astropart Phys.

[CR129] Duarte M, Brito R (2016). Asymptotically anti-de Sitter Proca stars. Phys Rev D.

[CR130] Dymnikova I, Koziel L, Khlopov M, Rubin S (2000). Quasilumps from first order phase transitions. Grav Cosmol.

[CR131] Dzhunushaliev V, Myrzakulov K, Myrzakulov R (2007). Boson stars from a gauge condensate. Mod Phys Lett A.

[CR132] Dzhunushaliev V, Folomeev V, Myrzakulov R, Singleton D (2008). Non-singular solutions to Einstein–Klein–Gordon equations with a phantom scalar field. J High Energy Phys.

[CR133] Dzhunushaliev V, Folomeev V, Singleton D (2011). Chameleon stars. Phys Rev D.

[CR134] Dzhunushaliev V, Folomeev V, Hoffmann C, Kleihaus B, Kunz J (2014). Boson stars with nontrivial topology. Phys Rev D.

[CR135] Eby J, Kouvaris C, Nielsen NG, Wijewardhana LCR (2016). Boson stars from self-interacting dark matter. J High Energy Phys.

[CR136] Eckart A, Hüttemann A, Kiefer C, Britzen S, Zajaček M, Lämmerzahl C, Stöckler M, Valencia-S M, Karas V, García-Marín M (2017) The Milky Way’s supermassive black hole: how good a case is it? A challenge for astrophysics & philosophy of science. Found Phys 47:553–624. 10.1007/s10701-017-0079-2. arXiv:1703.09118

[CR137] Emparan R, Reall HS (2008) Black holes in higher dimensions. Living Rev Relativ 11:lrr-2008-6. 10.12942/lrr-2008-6, http://www.livingreviews.org/lrr-2008-6. arXiv:0801.3471 10.12942/lrr-2008-6 PMC5253845 28163607

[CR138] Eto M, Hashimoto K, Iida H, Miwa A (2011). Chiral magnetic effect from Q-balls. Phys Rev D.

[CR139] Famaey B, McGaugh SS (2012) Modified Newtonian Dynamics (MOND): observational phenomenology and relativistic extensions. Living Rev Relativ 15:lrr-2012-10. 10.12942/lrr-2012-10, http://www.livingreviews.org/lrr-2012-10. arXiv:1112.3960 10.12942/lrr-2012-10 PMC5255531 28163623

[CR140] Fan Yz, Yang Rz, Chang J (2012) Constraining asymmetric bosonic non-interacting dark matter with neutron stars. ArXiv e-prints arXiv:1204.2564

[CR141] Faraoni V (2012). Correspondence between a scalar field and an effective perfect fluid. Phys Rev D.

[CR142] Feng JL (2010). Dark matter candidates from particle physics and methods of detection. Ann Rev Astron Astrophys.

[CR143] Flaminio R (2016). The cryogenic challenge: status of the KAGRA project. J Phys Conf Ser.

[CR144] Fodor G, Forgács P, Horváth Z, Lukacs A (2008). Small amplitude quasi-breathers and oscillons. Phys Rev D.

[CR145] Fodor G, Forgács P, Horváth Z, Mezei M (2009). Computation of the radiation amplitude of oscillons. Phys Rev D.

[CR146] Fodor G, Forgács P, Horváth Z, Mezei M (2009b) Oscillons in dilaton–scalar theories. J High Energy Phys 2009(08):106. 10.1088/1126-6708/2009/08/106. arXiv:0906.4160

[CR147] Fodor G, Forgács P, Horváth Z, Mezei M (2009c) Radiation of scalar oscillons in 2 and 3 dimensions. Phys Lett B 674:319–324. 10.1016/j.physletb.2009.03.054. arXiv:0903.0953

[CR148] Fodor G, Forgács P, Mezei M (2010). Boson stars and oscillatons in an inflationary universe. Phys Rev D.

[CR149] Fodor G, Forgács P, Mezei M (2010). Mass loss and longevity of gravitationally bound oscillating scalar lumps (oscillatons) in D-dimensions. Phys Rev D.

[CR150] Fodor G, Forgács P, Grandclément P (2015). Self-gravitating scalar breathers with negative cosmological constant. Phys Rev D.

[CR151] Font JA (2008) Numerical hydrodynamics and magnetohydrodynamics in general relativity. Living Rev Relativ 11:lrr-2008-7. 10.12942/lrr-2008-7. http://www.livingreviews.org/lrr-2008-7 10.12942/lrr-2008-7 PMC5256108 28179823

[CR152] Font JA, Goodale T, Iyer S, Miller M, Rezzolla L, Seidel E, Stergioulas N, Suen WM, Tobias M (2002). Three-dimensional numerical general relativistic hydrodynamics. II. Long-term dynamics of single relativistic stars. Phys Rev D.

[CR153] Franchini N, Pani P, Maselli A, Gualtieri L, Herdeiro CAR, Radu E, Ferrari V (2017). Constraining black holes with light boson hair and boson stars using epicyclic frequencies and quasiperiodic oscillations. Phys Rev D.

[CR154] Frank RL, Lenzmann E (2009a) On ground states for the $$l^2$$-critical boson star equation. ArXiv e-prints arXiv:0910.2721

[CR155] Frank RL, Lenzmann E (2009b) Uniqueness of ground states for the $$l^2$$-critical boson star equation. ArXiv e-prints arXiv:0905.3105

[CR156] Friedberg R, Lee TD, Pang Y (1987). Mini-soliton stars. Phys Rev D.

[CR157] Friedberg R, Lee TD, Pang Y (1987). Scalar soliton stars and black holes. Phys Rev D.

[CR158] Friedman JL, Ipser JR, Sorkin RD (1988). Turning-point method for axisymmetric stability of rotating relativistic stars. Astrophys J.

[CR159] Gentle SA, Rangamani M, Withers B (2012). A soliton menagerie in AdS. J High Energy Phys.

[CR160] Giudice GF, McCullough M, Urbano A (2016). Hunting for dark particles with gravitational waves. J Cosmol Astropart Phys.

[CR161] Gleiser M (1988). Stability of boson stars. Phys Rev D.

[CR162] Gleiser M, Jiang N (2015). Stability bounds on compact astrophysical objects from information-entropic measure. Phys Rev D.

[CR163] Gleiser M, Watkins R (1989). Gravitational stability of scalar matter. Nucl Phys B.

[CR164] Goetz AS (2015a) The Einstein–Klein–Gordon equations, wave dark matter, and the Tully–Fisher relation. PhD thesis, Duke University. arXiv:1507.02626

[CR165] Goetz AS (2015b) Tully–Fisher scalings and boundary conditions for wave dark matter. ArXiv e-prints arXiv:1502.04976

[CR166] González JA, Guzmán FS (2011). Interference pattern in the collision of structures in the Bose–Einstein condensate dark matter model: comparison with fluids. Phys Rev D.

[CR167] Gourgoulhon E (2012). $$3+1$$ formalism in general relativity: bases of numerical relativity. Lecture Notes in Physics.

[CR168] Gracia-Linares M, Guzman FS (2016). Accretion of supersonic winds on boson stars. Phys Rev D.

[CR169] Grandclément P (2016) Light rings and light points of boson stars. ArXiv e-prints arXiv:1612.07507

[CR170] Grandclément P, Fodor G, Forgács P (2011). Numerical simulation of oscillatons: extracting the radiating tail. Phys Rev D.

[CR171] Grandclément P, Somé C, Gourgoulhon E (2014). Models of rotating boson stars and geodesics around them: new type of orbits. Phys Rev D.

[CR172] Guenther RL (1995) A numerical study of the time dependent Schrödinger equation coupled with Newtonian gravity. PhD thesis, The University of Texas, Austin. http://laplace.physics.ubc.ca/Members/matt/Doc/Theses/

[CR173] Gundlach C, Leveque RJ (2011). Universality in the run-up of shock waves to the surface of a star. J Fluid Mech.

[CR174] Gundlach C, Martín-García JM (2007) Critical phenomena in gravitational collapse. Living Rev Relativ 10:lrr-2007-5. 10.12942/lrr-2007-5, http://www.livingreviews.org/lrr-2007-5. arXiv:0711.4620 10.12942/lrr-2007-5 PMC5256106 28179820

[CR175] Gundlach C, Please C (2009). Generic behaviour of nonlinear sound waves near the surface of a star: smooth solutions. Phys Rev D.

[CR176] Güver T, Emre Erkoca A, Hall Reno M, Sarcevic I (2014). On the capture of dark matter by neutron stars. J Cosmol Astropart Phys.

[CR177] Guzmán FS (2004). Evolving spherical boson stars on a 3D Cartesian grid. Phys Rev D.

[CR178] Guzmán FS (2007). Scalar fields: at the threshold of astrophysics. J Phys Conf Ser.

[CR179] Guzmán FS (2009) The three dynamical fates of boson stars. Rev Mex Fis 55:321–326. http://www.scielo.org.mx/scielo.php?pid=S0035-001X2009000400011&nrm=iso&script=sci_arttext

[CR180] Guzmán FS, Rueda-Becerril JM (2009). Spherical boson stars as black hole mimickers. Phys Rev D.

[CR181] Guzmán FS, Ureña-López LA (2006). Gravitational cooling of self-gravitating Bose condensates. Astrophys J.

[CR182] Hanna C, Johnson MC, Lehner L (2017) Estimating gravitational radiation from super-emitting compact binary systems. Phys Rev D 124042. 10.1103/PhysRevD.95.124042. arXiv:1611.03506

[CR183] Harrison BK, Thorne KS, Wakano M, Wheeler JA (1965). Gravitation theory and gravitational collapse.

[CR184] Hartmann B, Riedel J (2012). Glueball condensates as holographic duals of supersymmetric Q-balls and boson stars. Phys Rev D.

[CR185] Hartmann B, Riedel J (2013). Supersymmetric Q-balls and boson stars in ($$\text{d}+1$$) dimensions. Phys Rev D.

[CR186] Hartmann B, Kleihaus B, Kunz J, List M (2010). Rotating boson stars in five dimensions. Phys Rev D.

[CR187] Hartmann B, Kleihaus B, Kunz J, Schaffer I (2012). Compact boson stars. Phys Lett B.

[CR188] Hartmann B, Kleihaus B, Kunz J, Schaffer I (2013). Compact (A)dS boson stars and shells. Phys Rev D.

[CR189] Hartmann B, Riedel J, Suciu R (2013). Gauss–Bonnet boson stars. Phys Lett B.

[CR190] Hawley SH, Choptuik MW (2000). Boson stars driven to the brink of black hole formation. Phys Rev D.

[CR191] Hawley SH, Choptuik MW (2003). Numerical evidence for ‘multiscalar stars’. Phys Rev D.

[CR192] Henderson LJ, Mann RB, Stotyn S (2015). Gauss–Bonnet boson stars with a single Killing vector. Phys Rev D.

[CR193] Henriques AB, Liddle AR, Moorhouse RG (1989). Combined boson–fermion stars. Phys Lett B.

[CR194] Henriques AB, Liddle AR, Moorhouse RG (1990). Combined boson–fermion stars: configurations and stability. Nucl Phys B.

[CR195] Henriques AB, Liddle AR, Moorhouse RG (1990). Stability of boson–fermion stars. Phys Lett B.

[CR196] Herdeiro C, Radu E (2014). Ergosurfaces for Kerr black holes with scalar hair. Phys Rev D.

[CR197] Herdeiro C, Radu E (2015). Construction and physical properties of Kerr black holes with scalar hair. Class Quantum Grav.

[CR198] Herdeiro C, Kunz J, Radu E, Subagyo B (2015). Myers–Perry black holes with scalar hair and a mass gap: unequal spins. Phys Lett B.

[CR199] Herdeiro C, Radu E, Runarsson H (2016). Kerr black holes with Proca hair. Class Quantum Grav.

[CR200] Herdeiro CAR, Radu E (2014). Kerr black holes with scalar hair. Phys Rev Lett.

[CR201] Herdeiro CAR, Radu E (2015b) Asymptotically flat black holes with scalar hair: a review. Int J Mod Phys D 24:1542014. 10.1142/S0218271815420146, proceedings, 7th Black Holes Workshop 2014: Aveiro, Portugal, December 18–19, 2014. arXiv:1504.08209

[CR202] Herdeiro CAR, Radu E, Rúnarsson H (2015). Kerr black holes with self-interacting scalar hair: hairier but not heavier. Phys Rev D.

[CR203] Herdeiro CAR, Radu E, Rúnarsson HF (2016b) Spinning boson stars and Kerr black holes with scalar hair: the effect of self-interactions. Int J Mod Phys D 25:1641014. 10.1142/S0218271816410145, proceedings, 3rd Amazonian Symposium on Physics and 5th NRHEP Network Meeting is approaching: Celebrating 100 Years of General Relativity: Belem, Brazil. arXiv:1604.06202

[CR204] Hod S (2011). Quasinormal resonances of a massive scalar field in a near-extremal Kerr black hole spacetime. Phys Rev D.

[CR205] Hod S (2012) Stationary scalar clouds around rotating black holes. Phys Rev D 86:104026. 10.1103/PhysRevD.86.104026. [Erratum: Phys. Rev. D 86 (2012) 129902]. arXiv:1211.3202

[CR206] Honda EP (2000) Resonant dynamics within the nonlinear Klein–Gordon equation: much ado about oscillons. PhD thesis, The University of Texas, Austin. http://laplace.physics.ubc.ca/Members/matt/Doc/Theses/. arXiv:hep-ph/0009104

[CR207] Honda EP (2010). Fractal boundary basins in spherically symmetric $$\phi ^4$$ theory. Phys Rev D.

[CR208] Honda EP, Choptuik MW (2002). Fine structure of oscillons in the spherically symmetric $$\phi ^4$$ Klein–Gordon model. Phys Rev D.

[CR209] Horvat D, Marunović A (2013). Dark energy-like stars from nonminimally coupled scalar field. Class Quantum Gravity.

[CR210] Horvat D, Ilijić S, Kirin A, Narančić Z (2013). Formation of photon spheres in boson stars with a nonminimally coupled field. Class Quantum Gravity.

[CR211] Horvat D, Ilijic S, Kirin A, Narancic Z (2015). Note on the charged boson stars with torsion-coupled field. Phys Rev D.

[CR212] Horvat D, Ilijić S, Kirin A, Narančić Z (2015). Nonminimally coupled scalar field in teleparallel gravity: boson stars. Class Quantum Gravity.

[CR213] Hu S, Liu JT, Pando Zayas LA (2012) Charged boson stars in AdS and a zero temperature phase transition. ArXiv e-prints arXiv:1209.2378

[CR214] Jamison AO (2013). Effects of gravitational confinement on bosonic asymmetric dark matter in stars. Phys Rev D.

[CR215] Jetzer P (1989). Dynamical instability of bosonic stellar configurations. Nucl Phys B.

[CR216] Jetzer P (1989). Stability of charged boson stars. Phys Lett B.

[CR217] Jetzer P (1989). Stability of excited bosonic stellar configurations. Phys Lett B.

[CR218] Jetzer P (1990). Stability of combined boson–fermion stars. Phys Lett B.

[CR219] Jetzer P (1992). Boson stars. Phys Rep.

[CR220] Jetzer P, van der Bij JJ (1989). Charged boson stars. Phys Lett B.

[CR221] Jin KJ, Suen WM (2007). Critical phenomena in head-on collisions of neutron stars. Phys Rev Lett.

[CR222] Johannsen T, Wang C, Broderick AE, Doeleman SS, Fish VL, Loeb A, Psaltis D (2016). Testing general relativity with accretion-flow imaging of Sgr A*. Phys Rev Lett.

[CR223] Kan N, Shiraishi K (2016). Analytical approximation for Newtonian boson stars in four and five dimensions–a poor person’s approach to rotating boson stars. Phys Rev D.

[CR224] Kasuya S, Kawasaki M (2000). Q-ball formation through the Affleck–Dine mechanism. Phys Rev D.

[CR225] Kaup DJ (1968). Klein–Gordon geon. Phys Rev.

[CR226] Kellermann T, Rezzolla L, Radice D (2010). Critical phenomena in neutron stars: II head-on collisions. Class Quantum Gravity.

[CR227] Kesden M, Gair JR, Kamionkowski M (2005). Gravitational-wave signature of an inspiral into a supermassive horizonless object. Phys Rev D.

[CR228] Khachatryan V, Sirunyan AM, Tumasyan A, Adam W, Bergauer T, Dragicevic M, Erö J, Friedl M, Frühwirth R, Ghete VM (2015). Precise determination of the mass of the higgs boson and tests of compatibility of its couplings with the standard model predictions using proton collisions at 7 and 8. Eur Phys J C.

[CR229] Kichakova O, Kunz J, Radu E (2014). Spinning gauged boson stars in anti-de Sitter spacetime. Phys Lett B.

[CR230] Kichenassamy S (2008). Soliton stars in the breather limit. Class Quantum Gravity.

[CR231] Kiessling MKH (2009). Monotonicity of quantum ground state energies: Bosonic atoms and stars. J Stat Phys.

[CR232] Kleihaus B, Kunz J, List M (2005). Rotating boson stars and Q-balls. Phys Rev D.

[CR233] Kleihaus B, Kunz J, List M, Schaffer I (2008). Rotating boson stars and Q-balls. II. Negative parity and ergoregions. Phys Rev D.

[CR234] Kleihaus B, Kunz J, Lämmerzahl C, List M (2009). Charged boson stars and black holes. Phys Lett B.

[CR235] Kleihaus B, Kunz J, Lämmerzahl C, List M (2010). Boson shells harboring charged black holes. Phys Rev D.

[CR236] Kleihaus B, Kunz J, Schneider S (2012). Stable phases of boson stars. Phys Rev D.

[CR237] Kleihaus B, Kunz J, Yazadjiev S (2015). Scalarized hairy black holes. Phys Lett B.

[CR238] Kobayashi Y, Kasai M, Futamase T (1994). Does a boson star rotate?. Phys Rev D.

[CR239] Kouvaris C, Tinyakov P (2013). (not)-Constraining heavy asymmetric bosonic dark matter. Phys Rev D.

[CR240] Kouvaris C, Tinyakov PG (2010). Can neutron stars constrain dark matter?. Phys Rev D.

[CR241] Kühnel F, Rampf C (2014). Astrophysical Bose–Einstein condensates and superradiance. Phys Rev D.

[CR242] Kumar S, Kulshreshtha U, Kulshreshtha DS (2015). Boson stars in a theory of complex scalar field coupled to gravity. Gen Relativ Gravit.

[CR243] Kumar S, Kulshreshtha U, Kulshreshtha DS (2016). Charged compact boson stars and shells in the presence of a cosmological constant. Phys Rev D.

[CR244] Kunz J, Navarro-Lerida F, Viebahn J (2006). Charged rotating black holes in odd dimensions. Phys Lett B.

[CR245] Kusenko A, Steinhardt PJ (2001). $$q$$-Ball candidates for self-interacting dark matter. Phys Rev Lett.

[CR246] Kusmartsev FV, Mielke EW, Schunck FE (1991). Gravitational stability of boson stars. Phys Rev D.

[CR247] Lai CW (2004) A numerical study of boson stars. PhD thesis, The University of British Columbia, Vancouver. http://laplace.physics.ubc.ca/Members/matt/Doc/Theses/, arXiv:gr-qc/0410040

[CR248] Lai CW, Choptuik MW (2007) Final fate of subcritical evolutions of boson stars. ArXiv e-prints arXiv:0709.0324

[CR249] Landea IS, García F (2016). Charged Proca stars. Phys Rev D.

[CR250] Landsberg GL (2006). Black holes at future colliders and beyond. J Phys G Nucl Part Phys.

[CR251] Latifah S, Sulaksono A, Mart T (2014). Bosons star at finite temperature. Phys Rev D.

[CR252] Lee JW (2010). Is dark matter a BEC or scalar field?. J Kor Phys Soc.

[CR253] Lee JW (2010). Minimum mass of galaxies from BEC or scalar field dark matter. J Cosmol Astropart Phys.

[CR254] Lee JW, Lim S, Choi D (2008) BEC dark matter can explain collisions of galaxy clusters. ArXiv e-prints arXiv:0805.3827

[CR255] Lee TD (1987). Soliton stars and the critical masses of black holes. Phys Rev D.

[CR256] Lee TD, Pang Y (1989). Stability of mini-boson stars. Nucl Phys B.

[CR257] Lee TD, Pang Y (1992). Nontopological solitons. Phys Rep.

[CR258] Lenzmann E (2009). Uniqueness of ground states for pseudorelativistic hartree equations. Anal PDE.

[CR259] Lenzmann E, Lewin M (2011). On singularity formation for the $$l^2$$-critical boson star equation. Nonlinearity.

[CR260] Li XY, Harko T, Cheng KS (2012). Condensate dark matter stars. J Cosmol Astropart Phys.

[CR261] Liddle AR, Madsen MS (1992). The structure and formation of boson stars. Int J Mod Phys D.

[CR262] Lora-Clavijo FD, Cruz-Osorio A, Guzmán FS (2010). Evolution of a massless test scalar field on boson star space-times. Phys Rev D.

[CR263] Lue A, Weinberg EJ (2000). Gravitational properties of monopole spacetimes near the black hole threshold. Phys Rev D.

[CR264] Lynn BW (1989). Q-stars. Nucl Phys.

[CR265] Macedo CFB, Pani P, Cardoso V, Crispino LCB (2013). Astrophysical signatures of boson stars: quasinormal modes and inspiral resonances. Phys Rev D.

[CR266] Macedo CFB, Pani P, Cardoso V, Crispino LCB (2013). Into the lair: gravitational-wave signatures of dark matter. Astrophys J.

[CR267] Macedo CFB, Cardoso V, Crispino LCB, Pani P (2016). Quasinormal modes of relativistic stars and interacting fields. Phys Rev D.

[CR268] Madarassy EJM, Toth VT (2015). Evolution and dynamical properties of Bose–Einstein condensate dark matter stars. Phys Rev D.

[CR269] Maldacena JM (1998). The large-$$n$$ limit of superconformal field theories and supergravity. Adv Theor Math Phys.

[CR270] Marsh DJE, Pop AR (2015). Axion dark matter, solitons and the cusp-core problem. Mon Not R Astron Soc.

[CR271] Marunović A (2015) Boson stars with nonminimal coupling. ArXiv e-prints arXiv:1512.05718

[CR272] Marunović A, Murković M (2014). A novel black hole mimicker: a boson star and a global monopole nonminimally coupled to gravity. Class Quantum Gravity.

[CR273] Mazur PO, Mottola E (2001) Gravitational condensate stars: an alternative to black holes. ArXiv e-prints arXiv:gr-qc/0109035

[CR274] McGreevy J (2010). Holographic duality with a view toward many-body physics. Adv High Energy Phys.

[CR275] Meliani Z, Vincent FH, Grandclément P, Gourgoulhon E, Monceau-Baroux R, Straub O (2015). Circular geodesics and thick tori around rotating boson stars. Class Quantum Gravity.

[CR276] Meliani Z, Grandclément P, Casse F, Vincent FH, Straub O, Dauvergne F (2016). GR-AMRVAC code applications: accretion onto compact objects, boson stars versus black holes. Class Quantum Gravity.

[CR277] Mendes RFP, Yang H (2016) Tidal deformability of dark matter clumps. ArXiv e-prints arXiv:1606.03035

[CR278] Michelangeli A, Schlein B (2012). Dynamical collapse of boson stars. Commun Math Phys.

[CR279] Mielke EW (2016). Rotating boson stars. Fundam Theor Phys.

[CR280] Mielke EW, Scherzer R (1981). Geon-type solutions of the nonlinear Heisenberg–Klein–Gordon equation. Phys Rev D.

[CR281] Mielke EW, Schunck FE, Piran T, Ruffini R (1999). Boson stars: early history and recent prospects, gravitation and relativistic field theories. The Eighth Marcel Grossmann Meeting on recent developments in theoretical and experimental general relativity.

[CR282] Mielke EW, Schunck FE, Gurzadyan VG, Jantzen RT, Ruffini R (2002). Boson and axion stars. The Ninth Marcel Grossmann Meeting: on recent developments in theoretical and experimental general relativity, gravitation, and relativistic field theories.

[CR283] Milgrom M (1983). A modification of the Newtonian dynamics: implications for galaxies. Astrophys J.

[CR284] Milgrom M (2011). MOND—particularly as modified inertia. Acta Phys Pol B.

[CR285] Millward RS, Hirschmann EW (2003). Critical behavior of gravitating sphalerons. Phys Rev D.

[CR286] Mukherjee A, Shah S, Bose S (2015). Observational constraints on spinning, relativistic Bose–Einstein condensate stars. Phys Rev D.

[CR287] Mundim BC (2010) A numerical study of boson star binaries. PhD thesis, The University of British Columbia, Vancouver. http://laplace.physics.ubc.ca/Members/matt/Doc/Theses/. arXiv:1003.0239

[CR288] Murariu G, Puscasu G (2010). Solutions for Maxwell-equations’ system in a static conformal space-time. Rom J Phys.

[CR289] Murariu G, Dariescu C, Dariescu MA (2008). Maple routines for bosons on curved manifolds. Rom J Phys.

[CR290] Myers RC, Perry MJ (1986). Black holes in higher dimensional space-times. Ann Phys (NY).

[CR291] Ni Y, Zhou M, Cardenas-Avendano A, Bambi C, Herdeiro CAR, Radu E (2016). Iron k$$\alpha $$ line of Kerr black holes with scalar hair. J Cosmol Astropart Phys.

[CR292] Nogueira F (2013). Extremal surfaces in asymptotically AdS charged boson stars backgrounds. Phys Rev D.

[CR293] Núñez D, Degollado JC, Moreno C (2011). Gravitational waves from scalar field accretion. Phys Rev D.

[CR294] Okawa H (2015). Nonlinear evolutions of bosonic clouds around black holes. Class Quantum Gravity.

[CR295] Page DN (2004). Classical and quantum decay of oscillations: oscillating self-gravitating real scalar field solitons. Phys Rev D.

[CR296] Palenzuela C, Olabarrieta I, Lehner L, Liebling SL (2007). Head-on collisions of boson stars. Phys Rev D.

[CR297] Palenzuela C, Lehner L, Liebling SL (2008). Orbital dynamics of binary boson star systems. Phys Rev D.

[CR298] Pani P, Berti E, Cardoso V, Chen Y, Norte R (2009). Gravitational wave signatures of the absence of an event horizon: nonradial oscillations of a thin-shell gravastar. Phys Rev D.

[CR299] Pani P, Berti E, Cardoso V, Read J (2011). Compact stars in alternative theories of gravity: Einstein–Dilaton–Gauss–Bonnet gravity. Phys Rev D.

[CR300] Park SC (2012). Black holes and the LHC: a review. Prog Part Nucl Phys.

[CR301] Pena I, Sudarsky D (1997). Do collapsed boson stars result in new types of black holes?. Class Quantum Gravity.

[CR302] Petryk RJW (2006) Maxwell–Klein–Gordon fields in black hole spacetimes. PhD thesis, The University of British Columbia, Vancouver. http://laplace.physics.ubc.ca/Members/matt/Doc/Theses/

[CR303] Pisano F, Tomazelli JL (1996). Stars of WIMPs. Mod Phys Lett A.

[CR304] Polchinski J (2010) Introduction to gauge/gravity duality. In: Proceedings, theoretical advanced study institute in elementary particle physics (TASI 2010). String theory and its applications: from meV to the Planck scale: Boulder, Colorado, USA, June 1–25, 2010, pp 3–46. 10.1142/9789814350525_0001. arXiv:1010.6134

[CR305] Ponglertsakul S, Winstanley E, Dolan SR (2016). Stability of gravitating charged-scalar solitons in a cavity. Phys Rev D.

[CR306] Power EA, Wheeler JA (1957). Thermal geons. Rev Mod Phys.

[CR307] Psaltis D (2008) Probes and tests of strong-field gravity with observations in the electromagnetic spectrum. Living Rev Relativ 11:lrr-2008-9. 10.12942/lrr-2008-9, http://www.livingreviews.org/lrr-2008-9, arXiv:0806.1531 10.12942/lrr-2008-9 PMC5253923 28163608

[CR308] Pugliese D, Quevedo H, Rueda HJA, Ruffini R (2013). Charged boson stars. Phys Rev D.

[CR309] Radu E, Subagyo B (2012). Spinning scalar solitons in anti-de Sitter spacetime. Phys Lett B.

[CR310] Rangamani M, Takayanagi T (2017). Holographic entanglement entropy, Lecture Notes in Physics.

[CR311] Reid GD, Choptuik MW (2016). Nonminimally coupled topological-defect boson stars: static solutions. Phys Rev D.

[CR312] Rindler-Daller T, Shapiro PR (2012). Angular momentum and vortex formation in Bose–Einstein-condensed cold dark matter haloes. Mon Not R Astron Soc.

[CR313] Roger T, Maitland C, Wilson K, Westerberg N, Vocke D, Wright EM, Faccio D (2016) Optical analogues of the Newton–Schrödinger equation and boson star evolution. ArXiv e-prints arXiv:1611.00924 10.1038/ncomms13492 PMC5114577 27841261

[CR314] Rosen G (1966). Existence of particlelike solutions to nonlinear field theories. J Math Phys.

[CR315] Rousseau B (2003) Axisymmetric boson stars in the conformally flat approximation. Master’s thesis, The University of British Columbia, Vancouver. http://laplace.physics.ubc.ca/Members/matt/Doc/Theses/

[CR316] Ruffini R, Bonazzola S (1969). Systems of self-gravitating particles in general relativity and the concept of an equation of state. Phys Rev.

[CR317] Ruiz M, Degollado JC, Alcubierre M, Núñez D, Salgado M (2012). Induced scalarization in boson stars and scalar gravitational radiation. Phys Rev D.

[CR318] Ryder LH (1996). Quantum field theory.

[CR319] Sakai N, Tamaki T (2012). What happens to Q-balls if $$q$$ is so large?. Phys Rev D.

[CR320] Sakamoto K, Shiraishi K (1998). Boson stars with large selfinteraction in ($$2+1$$)-dimensions: an exact solution. J High Energy Phys.

[CR321] Sakamoto K, Shiraishi K (1998). Exact solutions for boson fermion stars in ($$2+1$$)-dimensions. Phys Rev D.

[CR322] Sanchis-Gual N, Herdeiro C, Radu E, Degollado JC, Font JA (2017). Numerical evolutions of spherical Proca stars. Phys Rev D.

[CR323] Schive HY, Chiueh T, Broadhurst T (2014). Cosmic structure as the quantum interference of a coherent dark wave. Nat Phys.

[CR324] Schunck FE, Mielke EW, Hehl FW, Puntigam RA, Ruder H (1996). Rotating boson stars, numerics, visualization. Relativity and scientific computing: computer algebra.

[CR325] Schunck FE, Mielke EW (2003). General relativistic boson stars. Class Quantum Gravity.

[CR326] Schunck FE, Torres DF (2000). Boson stars with generic self-interactions. Int J Mod Phys D.

[CR327] Schwabe B, Niemeyer JC, Engels JF (2016) Simulations of solitonic core mergers in ultralight axion dark matter cosmologies. Phys Rev D 94:043513. 10.1103/PhysRevD.94.043513. arXiv:1606.05151

[CR328] Seidel E, Suen WM (1990). Dynamical evolution of boson stars: perturbing the ground state. Phys Rev D.

[CR329] Seidel E, Suen WM (1991). Oscillating soliton stars. Phys Rev Lett.

[CR330] Seidel E, Suen WM (1994). Formation of solitonic stars through gravitational cooling. Phys Rev Lett.

[CR331] Sharma R, Karmakar S, Mukherjee S (2008) Boson star and dark matter. ArXiv e-prints arXiv:0812.3470

[CR332] Shen T, Zhou M, Bambi C, Herdeiro CAR, Radu E (2017). Iron K$$\alpha $$ line of Proca stars. J Cosmol Astropart Phys.

[CR333] Shibata M, Nakamura T (1995). Evolution of three-dimensional gravitational waves: harmonic slicing case. Phys Rev D.

[CR334] Shibata M, Yoshino H (2010). Bar-mode instability of rapidly spinning black hole in higher dimensions: numerical simulation in general relativity. Phys Rev D.

[CR335] Silveira V, de Sousa CMG (1995). Boson star rotation: a Newtonian approximation. Phys Rev D.

[CR336] Sirunyan AM et al (2017) Search for black holes in high-multiplicity final states in proton–proton collisions at sqrt(s) = 13 TeV. ArXiv e-prints arXiv:1705.01403

[CR337] Smolić I (2015). Symmetry inheritance of scalar fields. Class Quantum Gravity.

[CR338] Soni A, Zhang Y (2017). Gravitational waves from SU(N) glueball dark matter. Phys Lett B.

[CR339] Stewart I (1982). Catastrophe theory in physics. Rep Prog Phys.

[CR340] Stojkovic D (2003). Nontopological solitons in brane world models. Phys Rev D.

[CR341] Stotyn S, Mann RB (2012). Another mass gap in the BTZ geometry?. J Phys A.

[CR342] Stotyn S, Park M, McGrath P, Mann RB (2012). Black holes and boson stars with one Killing field in arbitrary odd dimensions. Phys Rev D.

[CR343] Stotyn S, Chanona M, Mann RB (2014). Numerical boson stars with a single Killing vector. II. The d $$=$$ 3 case. Phys Rev D.

[CR344] Stotyn S, Leonard CD, Oltean M, Henderson LJ, Mann RB (2014). Numerical boson stars with a single Killing vector I. The $$d\ge 5$$ case. Phys Rev D.

[CR345] Straumann N (1984). General relativity and relativistic astrophysics.

[CR346] Straumann N, Ehlers J, Schäfer G (1992). Fermion and boson stars. Relativistic gravity research with emphasis on experiments and observations. Lecture Notes in Physics.

[CR347] Tamaki T, Sakai N (2010). Unified picture of Q-balls and boson stars via catastrophe theory. Phys Rev D.

[CR348] Tamaki T, Sakai N (2011). Gravitating Q-balls in the Affleck–Dine mechanism. Phys Rev D.

[CR349] Tamaki T, Sakai N (2011). How does gravity save or kill Q-balls?. Phys Rev D.

[CR350] Tamaki T, Sakai N (2011). What are universal features of gravitating Q-balls?. Phys Rev D.

[CR351] Thorne KS, Klauder JR (1972). Nonspherical gravitational collapse: a short review. Magic without magic: John Archibald Wheeler. A collection of essays in honor of his sixtieth birthday.

[CR352] Torres DF, Capozziello S, Lambiase G (2000). Supermassive boson star at the Galactic center?. Phys Rev D.

[CR353] Unruh WG (2014) Has Hawking radiation been measured? Found Phys 44:532–545. 10.1007/s10701-014-9778-0, proceedings, Horizons of Quantum Physics: Taipei, Taiwan, October 14-18, 2012. ArXiv e-prints arXiv:1401.6612

[CR354] Ureña-López LA, Bernal A (2010). Bosonic gas as a galactic dark matter halo. Phys Rev D.

[CR355] Ureña-López LA, Matos T, Becerril R (2002). Inside oscillatons. Class Quantum Gravity.

[CR356] Valdez-Alvarado S, Becerril R, Ureña-López LA (2011) $$\phi ^{4}$$ oscillatons. ArXiv e-prints arXiv:1107.3135

[CR357] Valdez-Alvarado S, Palenzuela C, Alic D, Ureña-López LA (2013). Dynamical evolution of fermion–boson stars. Phys Rev D.

[CR358] Vilenkin A, Shellard EPS (1994). Cosmic strings and other topological defects. Cambridge Monographs on Mathematical Physics.

[CR359] Vincent FH, Gourgoulhon E, Herdeiro C, Radu E (2016). Astrophysical imaging of Kerr black holes with scalar hair. Phys Rev D.

[CR360] Vincent FH, Meliani Z, Grandclément P, Gourgoulhon E, Straub O (2016). Imaging a boson star at the Galactic center. Class Quantum Gravity.

[CR361] Wald RM (1984). General relativity.

[CR362] Wheeler JA (1955). Geons. Phys Rev.

[CR363] Will CM (2014) The confrontation between general relativity and experiment. Living Rev Relativ 17:lrr-2014-4. 10.12942/lrr-2014-4. http://www.livingreviews.org/lrr-2014-4. arXiv:1403.7377 10.12942/lrr-2014-4 PMC5255900 28179848

[CR364] Yagi K, Stein LC (2016). Black hole based tests of general relativity. Class Quantum Gravity.

[CR365] Yoshida S, Eriguchi Y (1997). Rotating boson stars in general relativity. Phys Rev D.

[CR366] Yuan YF, Narayan R, Rees MJ (2004). Constraining alternate models of black holes: type I X-ray bursts on accreting fermion–fermion and boson–fermion stars. Astrophys J.

[CR367] Yunes N, Yagi K, Pretorius F (2016). Theoretical physics implications of the binary black-hole mergers GW150914 and GW151226. Phys Rev D.

